# The Oxygenate-Mediated Conversion of CO_x_ to Hydrocarbons—On the Role of Zeolites in Tandem Catalysis

**DOI:** 10.1021/acs.chemrev.3c00058

**Published:** 2023-09-28

**Authors:** Jingxiu Xie, Unni Olsbye

**Affiliations:** † SMN Centre for Materials Science and Nanotechnology, Department of Chemistry, University of Oslo, Sem Sælands vei 26, 0315 Oslo, Norway.; § Green Chemical Reaction Engineering, Engineering and Technology Institute Groningen, University of Groningen, Nijenborgh 4, 9747 AG Groningen, The Netherlands

## Abstract

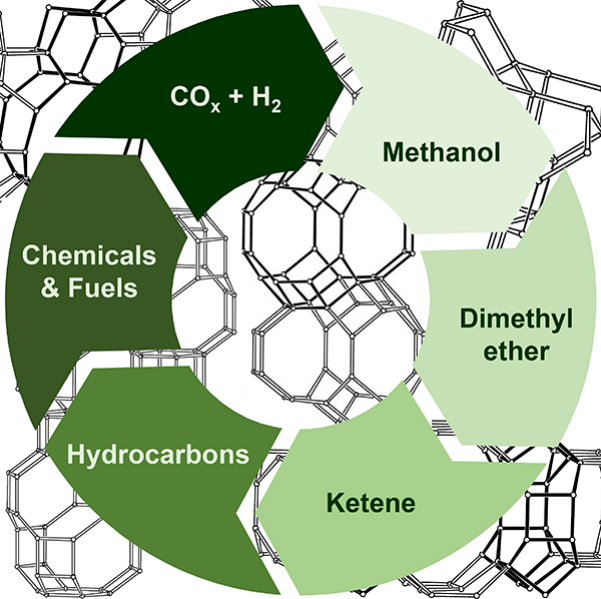

Decentralized chemical plants close to circular carbon sources will play an important role in shaping the postfossil society. This scenario calls for carbon technologies which valorize CO_2_ and CO with renewable H_2_ and utilize process intensification approaches. The single-reactor tandem reaction approach to convert CO_x_ to hydrocarbons via oxygenate intermediates offers clear benefits in terms of improved thermodynamics and energy efficiency. Simultaneously, challenges and complexity in terms of catalyst material and mechanism, reactor, and process gaps have to be addressed. While the separate processes, namely methanol synthesis and methanol to hydrocarbons, are commercialized and extensively discussed, this review focuses on the zeolite/zeotype function in the oxygenate-mediated conversion of CO_x_ to hydrocarbons. Use of shape-selective zeolite/zeotype catalysts enables the selective production of fuel components as well as key intermediates for the chemical industry, such as BTX, gasoline, light olefins, and C_3+_ alkanes. In contrast to the separate processes which use methanol as a platform, this review examines the potential of methanol, dimethyl ether, and ketene as possible oxygenate intermediates in separate chapters. We explore the connection between literature on the individual reactions for converting oxygenates and the tandem reaction, so as to identify transferable knowledge from the individual processes which could drive progress in the intensification of the tandem process. This encompasses a multiscale approach, from molecule (mechanism, oxygenate molecule), to catalyst, to reactor configuration, and finally to process level. Finally, we present our perspectives on related emerging technologies, outstanding challenges, and potential directions for future research.

## 1 Introduction

While the majority of current C,H-containing fuels and commodities are produced from fossil sources (coal, oil, natural gas), global warming and ocean acidification concerns have triggered a step change in studies of recycled CO and CO_2_ as carbon sources for the postfossil chemical industry. A recent IEAGHG Technical Report estimated the annual demand for methanol (an intermediate for production of, e.g., chemicals, polyolefins, and heavy duty truck fuel) and middle distillate hydrocarbons (for heavy duty trucks and aviation fuel production) of 825 and 903 megatons (Mt) per year, in the postfossil era.^1^ Aiming for a cyclic economy with Carbon Capture and Utilization (CCU), the CO_2_ abatement potential of current processes was roughly estimated to 1537 and 3349 Mt/year, respectively, for the two product groups, presuming use of “green” hydrogen. While these numbers are far from the current, global CO_2_ emission numbers (36.8 Mt in 2022),^2^ the potential for CCU is substantial.

Today, fossil sources are first transformed to synthesis gas (syngas, CO/H_2_) and/or CO_2_/H_2_ mixtures, which are subsequently reacted to valuable products. The conversion of CO/CO_2_/H_2_ to lower olefins, aromatics, and fuels occurs predominantly via two pathways, either via Fischer–Tropsch Synthesis (FTS) or via oxygenate intermediates (i.e., methanol, dimethyl ether, ketene).^3,4^

In the FTS process, typical FTS metals (Fe, Co, or Ru) are utilized with promoters to overcome the Anderson–Schulz–Flory distribution in order to attain the desired hydrocarbon distributions.^5,6^ The combination of sodium and sulfur was proven to suppress the formation of methane,^7,8^ while manganese oxide and alkali elements such potassium and sodium were effective in promoting chain growth in the hydrocarbon products.^5,6,9−12^ Alternatively, zeolites were used in combination with the FTS metals, in order to crack/isomerize longer hydrocarbons to the desired hydrocarbon distribution.^13,14^

The alternative, industrial pathway via oxygenate intermediates is a two-step process, in which synthesis gas is converted to methanol in a first reactor, and methanol is subsequently converted to lower olefins or to gasoline in a second reactor.^15,16^ Also here, zeolites are key to obtaining high selectivity to desired product groups. As an example, 91% of C_2_–C_4_ olefins selectivity was reported at full methanol conversion using a zeotype catalyst with CHA topology.^17^ In comparison, the highest C_2_–C_4_ olefins yield reported for the Fischer–Tropsch to Olefins (FTO) process (without combining the FTO catalysts with a zeolite) is 58%.^6^ An overview of zeolite structures discussed in this Review is provided in Table 1. The correlation between the product-limiting pore size of some central zeolite structures and the main effluent product groups obtained in the methanol to hydrocarbons (MTH) process are provided in Table 2.

**Table 1:** An Overview of Zeolite/Zeotype Topologies Used for Processes and Subprocesses Described Herein^a^

Three-letter structure code	Material common name(s)	Dimensionality and type of structure	Effluent product restricting pore(s) (diameter in Å)
AEI	SAPO-18	3D cavity-window	8-ring (3.8 × 3.8)
	MAPO-18		
	SSZ-39		
AEL	SAPO-11	1D straight channel	10-ring (4.0 × 6.5)
AFI	SAPO-5	1D straight channel	12-ring (7.3 × 7.3)
	MAPO-5		
	SSZ-24		
ATS	MAPO-36	1D straight channel	12-ring (6.5 × 7.5)
	SSZ-55		
BEA	Beta	3D disordered channel	12-ring
CHA	SAPO-34	3D cavity-window	8-ring (3.8 × 3.8)
	MAPO-34		
	SSZ-13		
	SAPO-47		
DDR	Sigma-1	2D cavity-window	8-ring (3.6 × 4.4)
	ZSM-58		
ERI	SAPO-17	3D cavity-window	8-ring (3.6 × 5.1)
ETL	EU-12	2D sinusoidal channel	8-ring (n.r.)
FAU	Zeolite X	3D cavity-window	12-ring (7.4 × 7.4)
	Zeolite Y		
FER	Ferrierite	2D channel	10-ring (4.2 × 5.4)
	ZSM-35		8-ring (3.5 × 4.8)
GON	GUS-1	1D channel	12-ring (5.4 × 6.8)
			8-ring (4.3 × 1.3)
IRN	ITQ-49	1D cavity-window	8-ring (n.r.)
LEV	MAPO-35	2D cavity-window	8-ring (3.6 × 4.8)
	RUB-1		
LTA	Linde Type A	3D cavity-window	8-ring (4.1 × 4.1)
	SAPO-42		
MEL	ZSM-11	3D straight channel	10-ring (5.3 × 5.4)
	Silicalite-2		
MFI	ZSM-5	3D straight/zigzag channel	10-ring (5.1 × 5.5)
	Silicalite-1		10-ring (5.3 × 5.6)
MOR	Mordenite	1D channel-pocket	12-ring (6.5 × 7.0)
			8-ring (2.6 × 5.7)
MTT	ZSM-23	1D straight channel	10-ring (4.5 × 5.2)
	EU-13		
MTW	ZSM-12	1D straight channel	12-ring (5.6 × 6.0)
MWW	MCM-22	2D channel	10-ring (4.0 × 5.5)
			10-ring (4.1 × 5.1)
RTH	RUB-13	2D cavity-window	8-ring (3.8 × 4.1)
	SSZ-50		8-ring (2.5 × 5.6)
RHO	DNL-6	3D cavity-window	8-ring (3.6 × 3.6)
SFH	SSZ-53	1D straight channel	14-ring (6.4 × 8.7)
SZR	SUZ-4	3D straight channel	10-ring (4.1 × 5.2)
			8-ring (3.2 × 4.8)
			8-ring (3.0 × 4.8)
TON	Theta-1	1D straight channel	10-ring (4.6 × 5.7)
	ZSM-22		
TUN	TNU-9	3D channel	10-ring (5.6 × 5.5)
			10-ring (5.4 × 5.5)
			10-ring (5.1 × 5.5)

a Structure data from the Database of Zeolite Structures by the International Zeolite Association.^45^ Abbreviations: n.r. Not reported.

**Table 2:** Largest and Main Product Groups Eluted from a Few Central Zeolites/Zeotypes during MTH Operation^a^

Three-letter structure code	Largest product group eluted from catalyst during MTH operation	Main product group during MTH operation
CHA	Linear aliphatics	C_2_–C_4_ olefins
TON	Monobranched aliphatics	C_5+_ aliphatics
MFI	Tetramethyl benzene	Propene, BTX, Gasoline^b^
BEA	Hexamethyl benzene	Aromatics, C_3+_ aliphatics

a MFI and CHA catalysts are in commercial use. Data from Teketel et al.^46^

b High temperature, moderate pressure (∼450 °C, 1 atm) favor olefins production. Moderate temperature, high pressure (∼350 °C, 20 atm) favor paraffins/aromatics production.

Moving from the hydrocarbon upgrading processes, significant progress has been achieved in recent years regarding C_1_ catalysis using bifunctional solid catalysts.^3,4,18−20^ Of particular interest is the direct pathway from CO/CO_2_/H_2_ feeds via an oxygenate (methanol/dimethyl ether/ketene) to form selected hydrocarbon product mixtures in a single reactor. The direct pathway requires bifunctional catalysts, i.e., a function to convert syngas or CO_2_/H_2_ to oxygenates and a function to convert oxygenates to hydrocarbons. It is an example of tandem catalysis, which refers to the presence of bi-/multi-catalytic functions operating under different mechanisms in a single reactor for chemical transformations.^21,22^ It offers opportunities to overcome the thermodynamic limitation and to intensify chemical processes through reducing the number of separation steps and workup of products. However, numerous challenges remain to be resolved, for instance, compatibility of reaction conditions, catalyst activation, and deactivation. Tandem catalysis has a longer heritage in homogeneous and heterogeneous catalytic systems in liquid batch phase, while heterogeneous catalytic systems in gaseous continuous phase are relatively limited.^21−25^ A classic example of bifunctional solid catalysts operating in continuous gaseous flow conditions is the hydrocracking/hydroisomerization of alkanes, in which the metal sites (e.g., Pt) and acid sites (e.g., zeolites) are responsible for (de)hydrogenation and cracking/isomerization reactions, respectively.^26^

**Figure 1:**
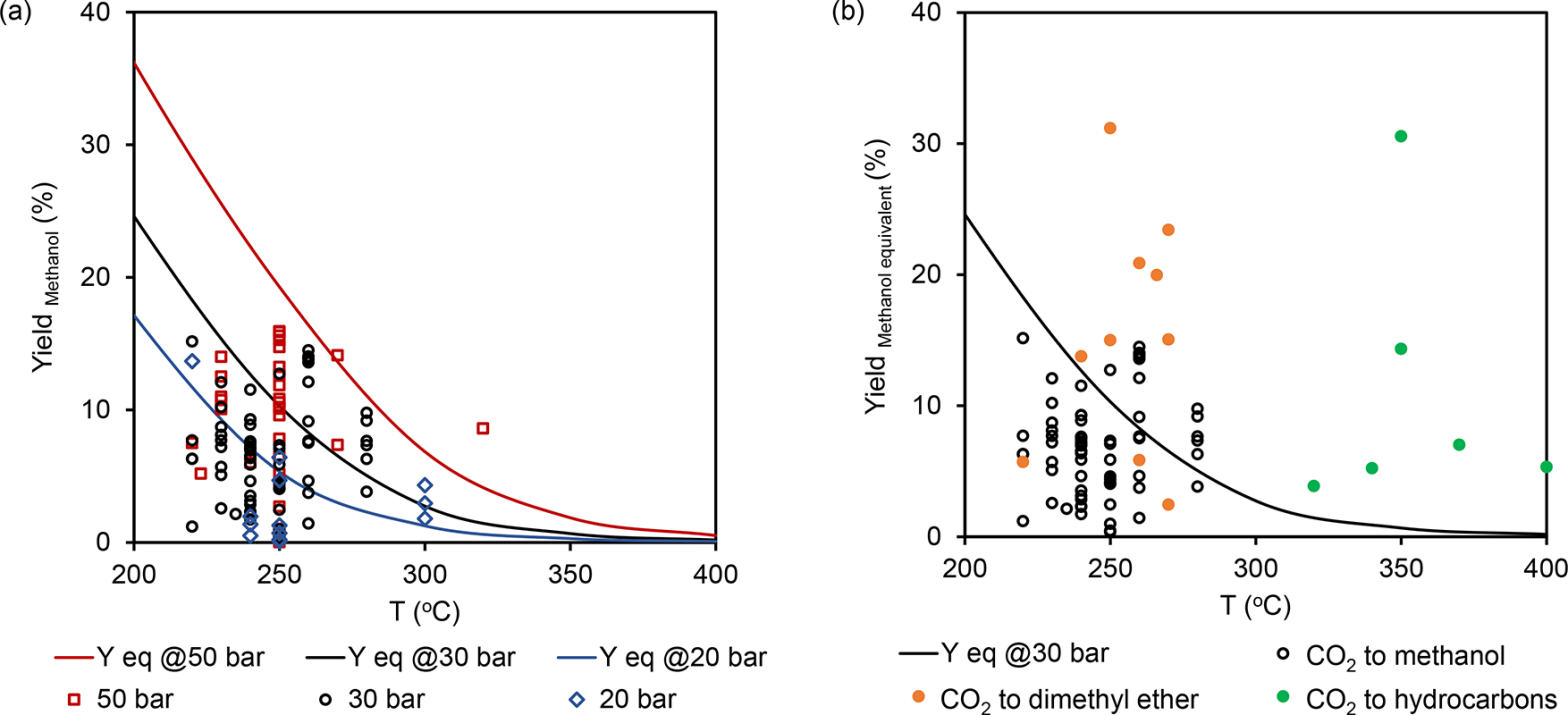
Thermodynamic considerations for CO_2_ hydrogenation to methanol, dimethyl ether, and hydrocarbons. (a) CO_2_ hydrogenation to methanol at 200 to 400 °C, 20 to 50 bar, and H_2_/CO_2_ = 3. Methanol yields were calculated from the data set in a recent review by Jiang et al.^28^ (b) CO_2_ hydrogenation to methanol, dimethyl ether, or hydrocarbons at 200 to 400 °C, 30 bar, and H_2_/CO_2_ = 3. Equivalent methanol yields from methanol, dimethyl ether, or hydrocarbon products were calculated from the data sets in recent reviews by Jiang et al., Saravanan et al., and Zhou et al. respectively.^3,28,29^

Returning to the title process, the addition of zeolites/zeotypes such as SAPO-34 to the CO_x_ hydrogenation catalysts creates a tandem catalytic system and shifts the thermodynamic equilibrium by applying Le Chatelier’s principle, as reflected in Figure 1. Using CO_2_ hydrogenation as a case study, Figure 1a shows that the yield of methanol is limited by thermodynamics over a range of reaction temperatures and pressures. The equilibrium yields of methanol are modeled using Aspen Plus according to the RGibbs block (PB-RM property method). Two equilibrium reactions are considered (CO_2_ to methanol or CO), and the products in the outlet stream are limited to CO_2_, H_2_, CO, methanol, and H_2_O. Experimental trends observed in Figure 1 are in accordance with thermodynamic predictions, but some data are seemingly above the equilibrium limit. The reason may be slightly different reaction conditions than those reported (e.g., higher P, lower T, different partial pressures). Thermodynamics suggest that higher methanol yields could be obtained at lower temperatures and higher pressures; thus, the conversion of CO_2_ to methanol is typically performed at 225 to 275 °C and 30 to 50 bar. The addition of zeolites/zeotypes to the CO_2_ hydrogenation catalysts converts the synthesized methanol directly to dimethyl ether or hydrocarbon products, therefore maintaining a relatively low methanol partial pressure in the reactor. The relatively low methanol partial pressure shifts the product selectivity from CO toward dimethyl ether or hydrocarbons, resulting in the theoretical yield of methanol (based on CO selectivity) to be higher than the thermodynamic equilibrium, as illustrated in Figure 1b. However, the accumulation of water with every step of reaction is thermodynamically the major disadvantage of the combined processes. Interestingly, zeolites in the reactor could also act as solid adsorbents to reduce steam partial pressure and further intensify the direct processes.^27^

Although there is a clear motivation for the combination of zeolites/zeotypes and CO_x_ hydrogenation catalytic functions to utilize the tandem catalysis concept, there is an additional complexity to the “usual” operations of the zeolites/zeotypes in individual reactions. For instance, the influence of a relatively high steam partial pressure and the effect of reactants and products from a previous reaction on the kinetics of the next reaction may be significant. Considering again the case of CO_2_ hydrogenation to methanol and its subsequent conversion to lower olefins in a single pass, CO_2_ hydrogenation to methanol has a typical reaction window of 225 to 275 °C and 20 to 50 bar, but the methanol to olefins process (MTO) has a typical reaction window above 350 °C and 1 bar. Besides reaction temperatures and pressures, the reactant partial pressures are also different. MTO is commonly carried out with a feed consisting of methanol and an inert gas. However, in the direct conversion of CO_2_ to lower olefins, the zeolites/zeotypes would be exposed to a gas mixture of CO_2_, H_2_, CO, H_2_O, and oxygenates.

**Figure 2:**
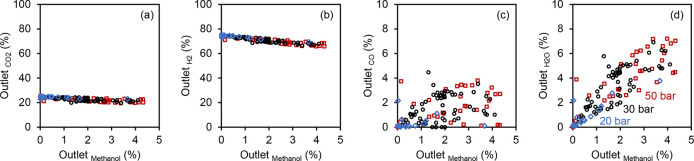
Partial pressures (in terms of % outlet pressure) of (a) CO_2_, (b) H_2_, (c) CO, and (d) H_2_O as a function of methanol partial pressure at the outlet of a CO_2_ hydrogenation reactor operating at 200 to 400 °C, 20 to 50 bar, and H_2_/CO_2_ = 3. They were calculated from the data set in a recent review by Jiang et al.^28^

The partial pressure of the reactants and products after CO_2_ hydrogenation is presented in terms of % outlet pressure in Figure 2, and both absolute partial pressures and relative partial pressures to methanol could further complicate the catalytic function of zeolites/zeotypes in the MTO reaction. From Figure 2, the methanol partial pressure increased with reaction pressure as governed by thermodynamics and accounted for up to 5% of the outlet stream. This is lower than what is typically used for methanol to hydrocarbons (MTH), and a much diluted methanol feed going to the zeolite/zeotype is in principle good for catalyst stability but compromises productivity rates. The partial pressure in the outlet stream followed the order of H_2_ > CO_2_ > H_2_O > CO or methanol. According to Figure 2a, the amount of CO_2_ in the outlet stream decreased from 25 to 20% as the methanol content increased from 0 to 5%. A feed ratio of H_2_/CO_2_ = 3 is required to produce equimolar amounts of methanol and water, so CO_2_ and H_2_ made up 25 and 75% of the feed stream, respectively. CO_2_ conversion is limited by equilibrium conversion and depends on kinetics, so a higher reaction pressure leads to higher conversion, and up to 27% conversion at 50 bar is reported. This results in a range of 20 to 25% CO_2_ in the outlet stream and corresponds to a CO_2_ to methanol ratio between 5 and 1000. The same reasoning applies for H_2_ in the outlet stream (Figure 2b) as H_2_ in the outlet stream decreased from 75 to 65% with an increase in methanol. The ratio of H_2_ to methanol was more drastic and ranged from 15 to 4000. On the other hand, the difference in the CO and methanol contents in the outlet stream was smaller, as revealed in Figure 2c. The correlation between CO and methanol was in general positive, with ratios of CO to methanol less than 3 and preferably less than 1. This is understandable since CO and methanol are products of competing reactions. These ratios of CO to methanol are lower than what is typically used for methanol and dimethyl ether (DME) carbonylation (vide infra, Section 4). Last but not least, Figure 2d shows the positive correlation between H_2_O and methanol, and the ratio of H_2_O to methanol has to be at least 1. Since the competing reverse water gas shift (RWGS) reaction also produces water, the ratios of H_2_O to methanol were between 1 and 4. The competitive sorption/reaction of steam and methanol over Brønsted acid sites could potentially affect the overall reaction rates, as well as, for example, relative rates of hydrogen transfer/alkene methylation. Therefore, it is worthwhile to dive deeper into the bifunctional catalysts for CO_x_ hydrogenation from the viewpoint of the zeolites/zeotypes catalytic function.

Patent literature reveals early industrial efforts in combining the conversion of syngas to oxygenates with oxygenate conversion to hydrocarbons. While the first efforts (from 1981) focused on a two-reactors-in-series approach, the first single-reactor tandem process was patented in 2001. An overview of patent literature is provided in Table 3. Challenges of this tandem process include the identification of suitable reaction conditions for both the operation of catalytic functions and the inhibition of side reactions. Early investigation on bifunctional catalysts for the direct conversion of synthesis gas via oxygenates concluded with paraffins being the main products,^30−32^ and the first success stories on light olefins formation were reported in 2016 independently by the groups of Bao and Wang.^33,34^ Bao and co-workers developed a bifunctional catalyst consisting of Zn-Cr oxide and mesoporous SAPO-34, and proposed that the new process Ox-Zeo operated via ketene as the key intermediate.^33^ Wang and co-workers reported bifunctional catalysts consisting of Zr-Zn oxides and SAPO-34 as active components for CO activation and C–C coupling steps, respectively, with methanol and dimethyl ether being proposed to be the reaction intermediates.^34^ Although different oxygenate intermediates were suggested in the two studies, SAPO-34 was used in both cases to convert the oxygenates to lower olefins, understandably due to its commercial success in the methanol to olefins (MTO) process.^35−37^

**Table 3:** An Overview of Patent Literature Describing Oxygenate-Mediated Processes for the Formation of Hydrocarbons from CO_x_ (x = 1, 2) and H_2_^a^

								Hydrocarbon selectivity (%C)				
Year	Company	Process (Feed)	Methanol synthesis catalyst	MTH cat.	T (°C)	P (bar)	X (% CO_x_)	CH_4_	C_2–4_^=^	C_2–4_^0^	C_5+_	ref.
1981	Metallgesellschaft	2-stage	Cu/Zn/V	H-ZSM-5	T_1_: 250	P_1_: 58	40	n.r.	n.r.	n.r.	n.r.	(47)
		CO_x_, H_2_			T_2_: 375	P_2_: 58						
2001	UOP/Norsk Hydro	3-stage	Cu/Zn/Cr	H-SAPO-34	T_1_: 150–450	P_1_: 1–10^3^	n.r.	<1	96	1	2	(48)
		CH_4_		H-SAPO-17	T_2_: 200–700	P_2_: 1–5						
2001	KAIST	H_2_/CO_2_ = 3	Cu/Zn/Zr	H-SAPO-5	350	28	32	4	89 (43% C_4_)		7	(49)
2001	KAIST	H_2_/CO_2_ = 3	Cu/Zn/Zr	Cu-SAPO-5	350	28	28	4	85 (49% C_4_)		12	(49)
2001	KAIST	H_2_/CO_2_ = 3	Cu/Zn/Zr	H-SAPO-34	400	28	35	4	96 (52% C_3_)		1	(49)
2001	KAIST	H_2_/CO_2_ = 3	Cu/Zn/Zr	Cu-SAPO-34	400	28	40	2	97 (55% C_3_)		1	(49)
2007	Japan Gas Synthesize	H_2_/CO = 2	Pd/Ca/Si	β-zeolite	375	41	74	7	0	84	9	(50)
2007	Japan Gas Synthesize	H_2_/CO = 2	Pd/Ca/Si	β-zeolite	375	51	87	8	0	87	5	(50)
2007	Japan Gas Synthesize	H_2_/CO = 2	Pd/Ca/Si	Y-zeolite	375	51	88	10	0	84	6	(50)
2011	Chevron	H_2_/CO = 2	Zn/Cr	H-ZSM-5	350	41	30	2	3	26	69	(51)
2011	Chevron	H_2_/CO = 2	Zn/Cr	Ga-ZSM-5	350	41	30	1	5	17	77	(51)
2013	Chevron	H_2_/CO/CO_2_ = 67/25/8	Zn/Cr	H-ZSM-5	380	81	23	1	1	26	72	(52)
2014	Sinopec	H_2_/CO/CO_2_ = 50/42/8	Cu/Al/K/C/Mn/Si	H-SAPO-34	290	10	91	n.r.	37	n.r.	n.r.	(53)
2016	Pioneer Energy	H_2_/CO = 2	Cu/Zn	Zn-ZSM-5	225–300	20	87	4	n.r.	84	9	(54)
2016	Pioneer Energy	H_2_/CO = 2	Cu/Zn/Al	Zn-ZSM-5	200–420	20	67	13	n.r.	84	3	(54)
2016	Pioneer Energy	2-stage	Cu/Zn/Al	Zn-ZSM-5	T_1_: 250	P_1_: 20	61	8	0	54	39	(54)
		H_2_/CO = 2			T_2_: 400	P_2_: 1						
2016	Pioneer Energy	2-stage	Cu/Zn/Al	Zn-ZSM-5	T_1_: 175–275	P_1_: 20	65	6	2	33	59	(54)
		H_2_/CO = 1			T_2_: 450	P_2_: 1						
2018	Dow	H_2_/CO = 3	Cu/Zn/Al	H-SAPO-34	330	20	61	4	<3	93	0	(55)
2018	Dow	H_2_/CO = 3	Cu/Zn/Al	H-SAPO-34	340	20	69	2	0	98	0	(55)
2018	Dow	H_2_/CO = 3	Cu/Zn/Al	H-SAPO-34	350	20	66	2	0	97	0	(55)
2018	Dow	H_2_/CO = 3	Cu/Zn/Al	H-SAPO-34	360	20	65	2	0	97	0	(55)
2018	Dow	H_2_/CO = 3	Cu/Zn/Al	H-SAPO-34	370	20	59	2	0	97	0	(55)
2018	Dow	H_2_/CO = 3	Cu/Zn/Al	H-SAPO-34	380	20	51	2	0	97	0	(55)
2018	Dow	H_2_/CO = 3	Cu/Zn/Al	H-SAPO-34	400	20	40	6	0	94	0	(55)
2018	Dow	H_2_/CO = 3	Cu/Zn/Al	H-SAPO-34	410	20	34	8	0	91	0	(55)
2018	Dow	H_2_/CO = 3	Cu/Zn/Al	H-SAPO-34	430	20	19	22	0	77	0	(55)
2018	Dow	H_2_/CO = 3	Cu/Zn/Al	H-SAPO-34	380	5	3	34	<2	64	0	(55)
2018	Dow	H_2_/CO = 3	Cu/Zn/Al	H-SAPO-34	380	35	74	4	0	96	0	(55)
2018	Dow	H_2_/CO = 3	Cu/Zn/Al	H-SAPO-34	380	50	81	5	<1	94	0	(55)
2018	Dow	H_2_/CO = 2	Cu/Zn/Al	H-SAPO-34	380	50	82	4	0	96	0	(55)
2018	Dow	H_2_/CO = 4	Cu/Zn/Al	H-SAPO-34	380	50	80	6	0	94	0	(55)
2018	Dow	H_2_/CO = 5	Cu/Zn/Al	H-SAPO-34	380	50	80	7	<1	92	0	(55)
2018	Dow	H_2_/CO = 6	Cu/Zn/Al	H-SAPO-34	380	50	79	8	<1	92	0	(55)
2018	Dow	H_2_/CO = 3	Cu/Zn/Al	H-SAPO-34	380	40	72	5	0	94	0	(55)
2018	Dow	H_2_/CO/CO_2_ = 67/23/10	Cu/Zn/Al	H-SAPO-34	380	40	58	3	<1	96	0	(55)
2018	Dow	H_2_/CO/CO_2_ = 60/20/20	Cu/Zn/Al	H-SAPO-34	380	40	46	3	<1	96	0	(55)
2018	Dow	H_2_/CO_2_ = 3	Cu/Zn/Al	H-SAPO-34	400	40	2^b^	23	0	70	0	(55)
2018	Dow	H_2_/CO_2_ = 3	Cu/Zn/Al	H-SAPO-34	375	40	5^b^	6	0	85	4	(55)
2018	Dow	H_2_/CO_2_ = 3	Cu/Zn/Al	H-SAPO-34	350	40	8^b^	5	0	83	6	(55)
2018	Dow	H_2_/CO_2_ = 3	Cu/Zn/Al	H-SAPO-34	325	40	4^b^	6	0	34	0	(55)
2018	Dow	H_2_/CO_2_ = 3	Cu/Zn/Al	H-SAPO-34	300	40	3^b^	0	0	1	0	(55)
2018	Dow	H_2_/CO_2_ = 3	Cu/Zn/Al	H-SAPO-34	350	28	2^b^	7	0	69	0	(55)
2018	Dow	H_2_/CO_2_ = 3	Cu/Zn/Al	H-SAPO-34	350	2	0^b^	4	0	84	4	(55)
2018	Dow	H_2_/CO_2_ = 1	Cu/Zn/Al	H-SAPO-34	350	40	2^b^	8	0	84	5	(55)
2018	Dow	H_2_/CO_2_ = 10	Cu/Zn/Al	H-SAPO-34	350	40	25^b^	7	0	91	5	(55)
2018	Dow	H_2_/CO = 3	Cr/Zn	H-SAPO-34	400	50	67	13	3	82	2	(55)
2018	Dow	H_2_/CO = 3	Cr/Zn	H-SAPO-34	400	70	82	14	1	83	2	(55)
2018	Dow	H_2_/CO = 3	Cu/Zn/Al	H-SAPO-5/H-SAPO-34	380	50	79	4	0	83	4	(55)
2018	Dow	H_2_/CO = 3	Cu/Zn/Al	H-SAPO-18	400	50	69	9	0	89	2	(55)
2018	Dow	H_2_/CO = 3	Cu/Zn/Al	β-zeolite	385	50	71	13	0	86	2	(55)
2019	Dow	H_2_/CO = 2	Cr/Zn	H-SAPO-34	400	20	43	8	51^c^	36^c^	<5	(56)
2019	Dow	H_2_/CO = 1.5	Cr/Zn	H-SAPO-34	400	20	35	11	45^c^	38^c^	<5	(56)
2019	Dow	H_2_/CO = 3	Cr/Zn	H-SAPO-34	400	20	50	10	20^c^	61^c^	<9	(56)
2019	Dow	H_2_/CO = 3	Cr/Zn	H-SAPO-34	400	50	76	6	5^c^	76^c^	<12	(56)
2019	Dow	H_2_/CO = 2	Cr/Zn	H-SAPO-34	400	70	38	16	1^c^	51^c^	<3	(56)
2019	Dow	H_2_/CO = 3	Cr/Zn	H-SAPO-34	400	70	80	14	2^c^	71^c^	<12	(56)
2019	Dow	H_2_/CO = 2	Cr/Zn	H-SAPO-34	450	20	32	43	2^c^	51^c^	<5	(56)
2020	DICP	H_2_/CO = 2	Cu/Zn/Al	H-SSZ-13	365	20	9^b^	2	38	51	10	(57)
2020	DICP	H_2_/CO = 2	Pd/Zn/Cr	H-SAPO-34	400	25	13^b^	2	16	80	3	(57)
2020	DICP	H_2_/CO = 2	Cu/Zn/Al/Cr/Co	H-SAPO-34	370	20	9^b^	3	67	19	11	(57)
2020	DICP	H_2_/CO = 2	Cu/Zn	H-ZSM-5	400	25	8^b^	1	50	41	8	(57)
2020	DICP	H_2_/CO = 2	Co/Zn/Al	H-SSZ-13	400	25	10^b^	8	64	13	17	(57)
2020	DICP	H_2_/CO = 2	Co/Zn/Ti	H-SAPO-34	400	20	5^b^	3	57	32	8	(57)
2020	DICP	H_2_/CO = 2	Cu/Zn/Mo	H-SSZ-13	360	8	4^b^	4	50	42	4	(57)
2020	DICP	H_2_/CO = 2	Cu/Zn/V	H-SSZ-13	360	8	4^b^	4	53	31	12	(57)
2020	DICP	H_2_/CO = 2	Cu/Zn/Mn	H-SSZ-13	360	8	4^b^	4	63	25	8	(57)
2020	DICP	H_2_/CO = 2	Pd/Zn/Zr	H-SSZ-13	380	15	4^b^	4	56	10	30	(57)
2020	DICP	H_2_/CO = 2	Mo/Zn/Zr/Al	H-SAPO-34	400	30	5^b^	7	58	26	9	(57)
2020	DICP	H_2_/CO = 2	Zn/Ti/Ga	H-SAPO-34	400	35	4^b^	3	57	33	7	(57)
2020	DICP	H_2_/CO = 2	Co/Zn/Ti/Mn	H-SAPO-34	400	35	7^b^	5	68	25	2	(57)
2020	DICP	H_2_/CO = 2	Ca/Mn/Al/Cu/Zn	H-SSZ-13	375	37	16^b^	3	18	74	5	(57)
2020	DICP	H_2_/CO = 2	Ca/Mn/Cr/Co	H-MgSAPO-34	415	30	16^b^	3	16	75	6	(57)
2020	DICP	H_2_/CO = 2	Fe/Zn/Al/Cr/Na	H-SAPO-34	400	30	9^b^	6	66	21	7	(57)
2020	DICP	H_2_/CO = 2	Fe/Zn/Al/Cr/Mg	H-SAPO-34	400	30	10^b^	3	73	17	8	(57)
2020	DICP	H_2_/CO = 2	Fe/Zn/Al/Cr/K	H-SAPO-34	400	30	8^b^	9	76	9	6	(57)
2020	DICP	H_2_/CO = 2	Fe/Zn/Al/Cr/Ce	H-SAPO-34	400	30	8^b^	11	67	18	3	(57)
2020	DICP	H_2_/CO = 2	Fe/Zn/Al/Cr/La	H-SAPO-34	400	30	11^b^	2	73	13	12	(57)
2020	DICP	H_2_/CO = 2	Fe/Zn/Ga/Cr/Mn	H-SAPO-34	400	30	18^b^	3	75	20	2	(57)
2020	DICP	H_2_/CO = 2	Fe/Zn/Ge/Cr/Mn	H-SAPO-34	400	30	16^b^	2	70	21	7	(57)
2020	DICP	H_2_/CO = 2	Fe/Zn/Zr/Cr/Mn	H-SAPO-34	400	30	19^b^	4	75	17	4	(57)
2020	DICP	H_2_/CO = 2	Fe/Zn/In/Cr/Mn	H-SAPO-34	400	30	19^b^	6	66	21	7	(57)
2020	DICP	H_2_/CO = 2	Fe/Mn/Al/Cr/Na	H-SAPO-34	400	30	9^b^	6	64	25	6	(57)
2020	DICP	H_2_/CO = 2	Fe/Mn/Al/Cr/Mg	H-SAPO-34	400	30	10^b^	3	73	17	8	(57)
2020	DICP	H_2_/CO = 2	Fe/Mn/Al/Cr/K	H-SAPO-34	400	30	8^b^	9	74	11	6	(57)
2020	DICP	H_2_/CO = 2	Fe/Mn/Al/Cr/Ce	H-SAPO-34	400	30	10^b^	16	60	15	9	(57)
2020	DICP	H_2_/CO = 2	Fe/Mn/Al/Cr/La	H-SAPO-34	400	30	10^b^	12	70	16	2	(57)
2021	SXICC	H_2_/CO = 1.5	Zn/Mn/In/Ga/Ni/Cr/K	H-SAPO-34	420	20	14^b^	7	84	7	2	(58)
2021	SXICC	H_2_/CO = 2.5	Zn/Mn/In/Ga/Ni/Cr/K	H-SAPO-34	400	30	23^b^	7	80	11	2	(58)
2021	SXICC	H_2_/CO = 2.5	Zn/Mn/In/Ga/Ni/Cr/K	H-SAPO-34	380	50	14^b^	4	84	11	2	(58)
2021	SXICC	H_2_/CO = 2.5	Zn/Mn/In/Ga/Ni/Cr/K	H-SAPO-34	390	50	21^b^	6	80	10	4	(58)
2021	SXICC	H_2_/CO = 2.5	Zn/Mn/In/Ga/Ni/Cr/K	H-SAPO-34	400	50	18^b^	9	78	11	2	(58)
2021	SXICC	H_2_/CO = 2.5	Zn/Mn/In/Ga/Ni/Cr/K	H-SAPO-34	390	60	30^b^	8	81	9	2	(58)
2021	SXICC	H_2_/CO = 3.5	Zn/Mn/In/Ga/Ni/Cr/K	H-SAPO-34	400	60	29^b^	10	71	18	1	(58)
2021	SXICC	H_2_/CO = 2.5	Zn/Mn/In/Ga/Ni/Cr/K	H-SSZ-13	390	50	19^b^	4	85	6	4	(58)
2021	SXICC	H_2_/CO = 1.5	Zn/Mn/In/Ga/Ni/Cr/K	H-ZSM-5	380	80	16^b^	6	82	9	3	(58)
2021	SXICC	H_2_/CO = 2.5	Zn/Mn/In/Ga/Ni/Cr/K	H-ZSM-5	410	50	20^b^	6	76	16	3	(58)
2021	SXICC	H_2_/CO = 3.5	Zn/Mn/In/Ga/Ni/Cr/K	H-ZSM-5	380	70	26^b^	8	75	15	2	(58)
2021	TYUT	H_2_/CO = 2	Zn/Zr	H-SAPO-34/Silicate-1	410	20	9^b^	12	80	6	2	(59)
2021	DICP	2-stage	Zn/Al	H-SAPO-34	T_1_: 390	40	28	n.r.	82	n.r.	n.r.	(60)
		H_2_/CO = 1			T_2_: 390							
2021	DICP	2-stage	Zn/Al/Cr	H-SAPO-18	T_1_: 390	40	33	n.r.	80	n.r.	n.r.	(60)
		H_2_/CO = 1			T_2_: 390							
2021	DICP	2-stage	Zn/Al/Zr	H-SSZ-13	T_1_: 390	40	31	n.r.	78	n.r.	n.r.	(60)
		H_2_/CO = 1			T_2_: 390							
2021	DICP	2-stage	Cu/Zn/Al	H-SAPO-34	T_1_: 260	40	58	n.r.	76	n.r.	n.r.	(60)
		H_2_/CO = 1			T_2_: 390							
2021	DICP	2-stage	Cu/Zn/Al + Al_2_O_3_	H-SAPO-34	T_1_: 260	40	46	n.r.	77	n.r.	n.r.	(60)
		H_2_/CO = 1			T_2_: 390							
2022	SXICC	H_2_/CO_2_ = 3	Ga/Zr	H-SSZ-13	350	30	23^b^	4	2	94	0	(61)
2022	SXICC	H_2_/CO_2_ = 3	Cr	H-SAPO-34	370	5	9^b^	1	96	2	1	(62)
2022	SXICC	H_2_/CO_2_ = 3	Cr/Zn	H-SAPO-34	370	5	5^b^	5	89	3	2	(62)
2022	SXICC	H_2_/CO_2_ = 3	Cr/In	H-SAPO-34	370	5	7^b^	8	87	3	2	(62)
2022	SXICC	H_2_/CO_2_ = 3	Cr/Al	H-SAPO-34	370	5	5^b^	5	85	3	2	(62)
2022	SXICC	H_2_/CO_2_ = 3	Cr/Zr	H-SAPO-34	370	5	5^b^	7	85	5	3	(62)
2022	Sinopec	H_2_/CO = 1	Cr	H-ZSM-5	395	20	18	n.r.	n.r.	n.r.	80^d^	(63)
2022	Sinopec	H_2_/CO = 1	Mn	H-ZSM-5	395	20	19	n.r.	n.r.	n.r.	82^d^	(63)
2022	Sinopec	H_2_/CO = 1	Ce	H-ZSM-5	395	20	16	n.r.	n.r.	n.r.	73^d^	(63)
2022	Sinopec	H_2_/CO = 1	Zr	H-ZSM-5	395	20	14	n.r.	n.r.	n.r.	84^d^	(63)
2022	Sinopec	H_2_/CO = 1	Zn/Cr	H-ZSM-5	395	20	18	n.r.	n.r.	n.r.	79^d^	(63)
2022	Sinopec	H_2_/CO = 1	Zn/Mn	H-ZSM-5	395	20	20	n.r.	n.r.	n.r.	78^d^	(63)
2022	Sinopec	H_2_/CO = 1	Zr/In	H-ZSM-5	395	20	15	n.r.	n.r.	n.r.	82^d^	(63)
2022	Sinopec	H_2_/CO = 1	Cr/Mn	H-ZSM-5	395	20	18	n.r.	n.r.	n.r.	82^d^	(63)
2022	Sinopec	H_2_/CO = 1	Zr/Ce	H-ZSM-5	395	20	15	n.r.	n.r.	n.r.	81^d^	(63)
2022	Sinopec	H_2_/CO = 1	Mn	H-ZSM-11	395	20	20	n.r.	n.r.	n.r.	81^d^	(63)
2022	Sinopec	H_2_/CO = 1	Mn	H-ZSM-5/Silicalite-1	395	20	17	n.r.	n.r.	n.r.	84^d^	(63)
2022	Sinopec	H_2_/CO = 1	Mn	H-ZSM-5/Silicalite-2	395	20	17	n.r.	n.r.	n.r.	83^d^	(63)
2022	Sinopec	H_2_/CO = 1	Mn	H-ZSM-11/Silicalite-2	395	20	17	n.r.	n.r.	n.r.	83^d^	(63)
2022	Sinopec	H_2_/CO = 1	Mn	H-ZSM-11/Silicalite-1	395	20	18	n.r.	n.r.	n.r.	83^d^	(63)
2022	Sinopec	H_2_/CO = 1	Mn	H-ZSM-5	350	80	33	n.r.	n.r.	n.r.	72^d^	(63)
2022	Sinopec	H_2_/CO = 0.5	Mn	H-ZSM-5	395	80	28	n.r.	n.r.	n.r.	73^d^	(63)
2022	Sinopec	H_2_/CO = 4	Mn	H-ZSM-5	350	50	25	n.r.	n.r.	n.r.	68^d^	(63)
2022	Sinopec	H_2_/CO = 1	Mn	H-ZSM-5	395	50	20	n.r.	n.r.	n.r.	77^d^	(63)
2022	Sinopec	H_2_/CO = 1	Mn	H-ZSM-5	450	50	23	n.r.	n.r.	n.r.	72^d^	(63)
2022	Sinopec	H_2_/CO = 0.5	Mn	H-ZSM-5	395	40	20	n.r.	n.r.	n.r.	79^d^	(63)
2022	DICP	H_2_/CO = 2	Zn	H-SAPO-34/18	410	35	24	5	86	5	4	(64)
2022	DICP	H_2_/CO = 5.5	Zn	H-SAPO-34/18	400	9	38	5	85	8	2	(64)
2022	DICP	H_2_/CO = 3	Zn	H-SAPO-34/18	380	45	24	3	85	7	5	(64)
2022	DICP	H_2_/CO = 6	Mn	H-SAPO-34/18	370	100	33	7	71	14	8	(64)
2022	DICP	H_2_/CO = 3.5	Ce	AlPO-34/18	470	15	28	4	77	11	8	(64)
2022	DICP	H_2_/CO = 4.5	Bi	H-SAPO-34/18	400	70	56	3	83	9	5	(64)
2022	DICP	H_2_/CO = 6.5	In	H-SAPO-34/18	380	25	37	7	76	8	9	(64)
2022	DICP	H_2_/CO = 8.5	Ga	AlPO-34/18	370	5	31	7	69	11	13	(64)
2022	DICP	H_2_/CO = 1	Zn/Cr	H-SSZ-13/39	370	35	28	5	74	7	14	(64)
2022	DICP	H_2_/CO = 2.5	Zn/Al	H-SAPO-34/18	410	50	37	2	88	5	5	(64)
2022	DICP	H_2_/CO = 2.5	Zn/Ga	H-GaAPO-34/18	430	30	58	5	82	6	7	(64)
2022	DICP	H_2_/CO = 1	Zn/In	H-GaSAPO-34/18	520	40	19	6	87	6	1	(64)
2022	DICP	H_2_/CO = 0.5	Mn/Cr	H-ZnAPO-34/18	480	90	14	7	68	12	13	(64)
2022	DICP	H_2_/CO = 3	Mn/Al	H-MgAPO-34/18	470	60	38	3	86	6	5	(64)
2022	DICP	H_2_/CO = 1.5	Mn/Zr	H-GaAPO-34/18	450	50	37	2	86	6	6	(64)
2022	DICP	H_2_/CO = 2.5	Mn/In	H-SAPO-34/18	450	50	34	3	88	6	3	(64)
2022	DICP	H_2_/CO = 3.5	Co/Al	H-SAPO-34/18	350	50	27	3	76	10	11	(64)
2022	DICP	H_2_/CO = 6	Fe/Al	H-SAPO-34/18	350	70	20	5	85	7	3	(64)
2022	DICP	H_2_/CO = 4	In/Al/Mn	H-SAPO-34/18	400	60	58	5	80	6	9	(64)
2022	DICP	H_2_/CO = 4	In/Ga/Mn	AlPO-34/18	400	40	33	7	73	7	13	(64)

a Abbreviations: n.r. Not reported.

b % Conversion to hydrocarbons, i.e., exclude (R)WGS activity.

c C_2_–C_3_ selectivity.

d Aromatics selectivity.

**Figure 3:**
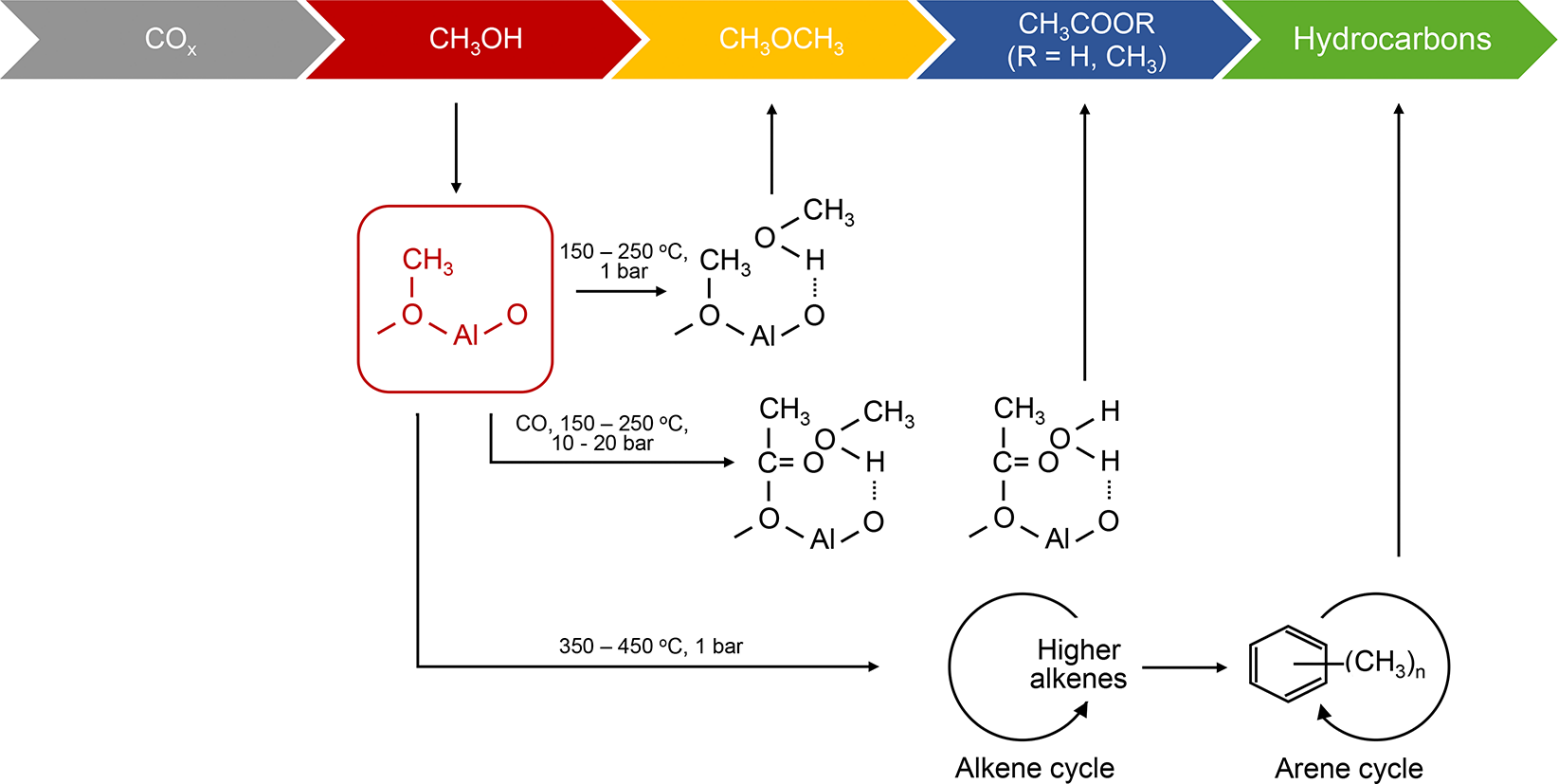
Illustration of the direct conversion of CO_x_ to hydrocarbons via oxygenate intermediates. Three relevant intermediates, namely, methanol, DME, and ketene (acetyl species), are discussed in this review.

This Review highlights the opportunities, challenges, prior learnings, and future research in the zeolites/zeotypes catalytic function for the direct conversion of CO_x_ to valuable products via oxygenate intermediates. There are recent reviews regarding the advances in C_1_ catalysis,^3,38,39^ CO_2_ to methanol,^28,40,41^ and methanol to hydrocarbons (MTH),^37,42−44^ demonstrating the relevance and importance of these research fields. Nonetheless, there has not yet been a critical discussion of the zeolite/zeotype catalytic function in the context of CO_x_ valorization using tandem catalysis. Central to zeolite/zeotype functionality in this context is their Brønsted acid properties and, as will be further elaborated in the following sections, their ability to form and maintain a population of methoxy sites (Figure 3).

## 2 Methanol-Mediated CO_x_ Conversion to Hydrocarbons

### 2.1 A Short Introduction to the MTH Process

To date, most studies devoted to oxygenate-mediated conversion of CO_x_ to hydrocarbons focused on methanol as the intermediate. As background to the discussion of various process options below, we will first give a brief introduction to the methanol to hydrocarbons (MTH) process. The MTH process (subdivided into olefins (MTO), gasoline (MTG), propene (MTP), and aromatics (MTA)) is a major industrial process for the conversion of C_1_ feedstock (natural gas, biogas, coal) to light olefins (MTO, MTP) and has been a topic of more than 6000 research papers over the last 10 years (source: Web of Science, May 16, 2022. Search words: Methanol to hydrocarbons, *or* methanol to gasoline, *or* methanol to olefins). Its academic interest stems partly from its industrial significance, and partly from fundamental studies published before the major commercialization wave (1977–2010). Those studies enabled the distinction of competing reactions taking place inside the microporous network of the zeolite/zeotype, based on the product distribution in the reactor effluent (see below). As such, fundamental studies of the MTH reaction have contributed greatly to the fundamental understanding of zeolites and zeotypes in general. The early studies are well documented in prior review and perspective articles,^35,65−69^ and we will only present a few of the early key contributions that are particularly relevant for the methanol-mediated CO_x_ to hydrocarbons reaction.

**Figure 4:**
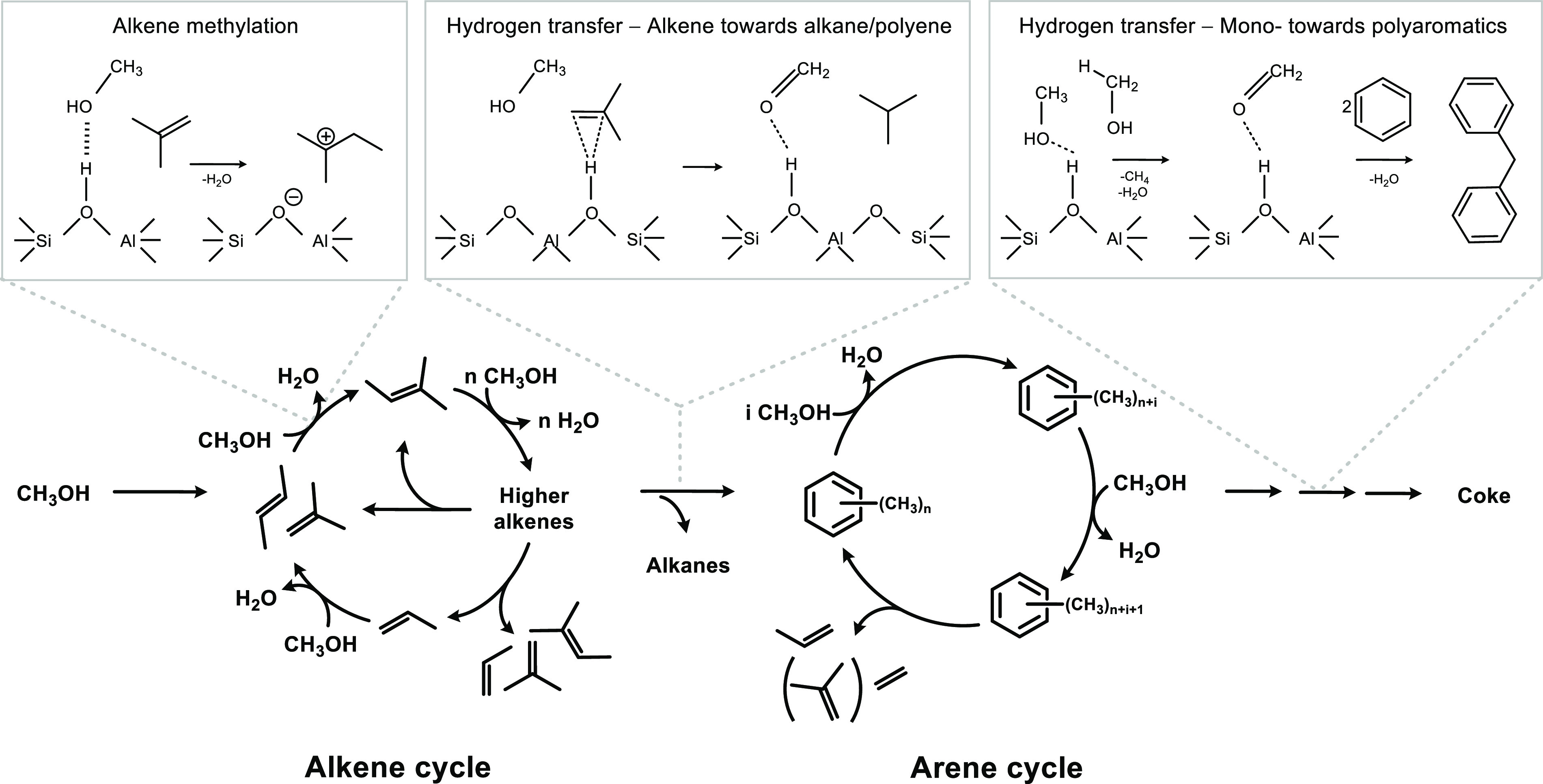
Dual cycle mechanism for the conversion of methanol to hydrocarbons, adapted with permission from ref (88). Copyright 2013 Elsevier. Mechanistic details in insets are extracted with permission from refs (89) and (90). Copyright 2017 Elsevier.

Early studies showed that the MTH reaction is autocatalytic, and that alkenes and methyl benzenes act as cocatalytic species.^70−80^ They further suggested a sequential reaction scheme, where methanol is first converted to DME, followed by olefin formation and methylation to higher alkenes. Subsequently, alkenes are converted into alkanes and aromatics, and finally to coke.^81^ Water is a coproduct of all reaction steps. The “hydrocarbon pool” concept next presented by Dahl and Kolboe was based on isotopic labeling studies over H-SAPO-34 and broke with the sequential scheme, suggesting instead that all products are formed from a pool of hydrocarbon species confined inside the zeolite/zeotype.^82,83^ Subsequent studies combined isotopic labeling with a technique developed by the Guisnet group, in which the zeolite lattice is dissolved after reaction, without altering the hydrocarbons retained inside the zeolite crystals.^84^ Those studies led to a next groundbreaking discovery, i.e., that propene (and higher alkenes) is formed by alkene cracking as well as polymethyl benzene dealkylation, while ethene is formed predominantly by polymethyl benzene dealkylation, under typical MTH conditions (350–450 °C, 1 bar).^85,86^ This discovery is the basis of the generally accepted “dual cycle” reaction scheme, which combines sequential and pool-type reaction concepts (Figure 4). The dual cycle concept paved the way for using the C_3_/C_2_ ratio in the reactor effluent as a descriptor for the abundance of the alkene versus the arene cycle in various zeolites. The Hydrogen Transfer Index, HTI = [C_n_^–^ / (C_n_^=^ + C_n_^–^)], where C_n_^=^ and C_n_^–^ are an alkene and alkane, respectively, with n carbon atoms, is another useful descriptor.^87^ It is based on the observation of a linear correlation between alkane and arene yields in the effluent of wide-pore zeolites, suggesting that aromatic compounds are mainly formed by hydrogen transfer between alkenes (and (cyclic) polyenes) rather than dehydrogenation reactions. These two descriptors are extremely useful for deriving mechanistic information from conventional screening tests of zeolite and zeotype catalysts.

Zeolites/zeotypes are molecular sieves, and the kinetic diameter of the smallest pore sets an upper limit to the size of the majority of products that may elute from a material with a given topology (Tables 1–2). Beneath that size, product selectivity relies on a subtle interplay between diffusivity and intrinsic reaction rates, commonly described by the Thiele modulus.^91^ On the macroscopic level, correlations were found between MTH product selectivity and parameters such as crystal diameter, acid site density, reaction temperature, and methanol pressure. Yet, the plethora of products contained in the zeolite pores during operation, and in some cases a diversity of active site framework positions, complicate the elucidation of structure–function correlations. In that respect, the successful synthesis of Zeolite Socony Mobil–5 (ZSM-5) nanosheets by Ryoo and co-workers represented a turning point of zeolite research, by enabling comparisons of catalyst effectiveness factor and product selectivity between conventional crystals and nanosheets with negligible diffusion length.^92,93^ For MTH, it was shown that the reaction’s autocatalytic nature leads to an inverse effectiveness factor, but only to minor changes in product distribution between 1 and 3 μm ZSM-5 crystals and 2–4 nm ZSM-5 nanosheets with similar acid site density.^94^ Later studies showed that larger ZSM-5 crystals yield less arenes and more ethene compared to smaller crystals, indicating promotion of the arene cycle by the longer diffusion length resulting in higher abundance of retained arenes.^95^

**Figure 5:**
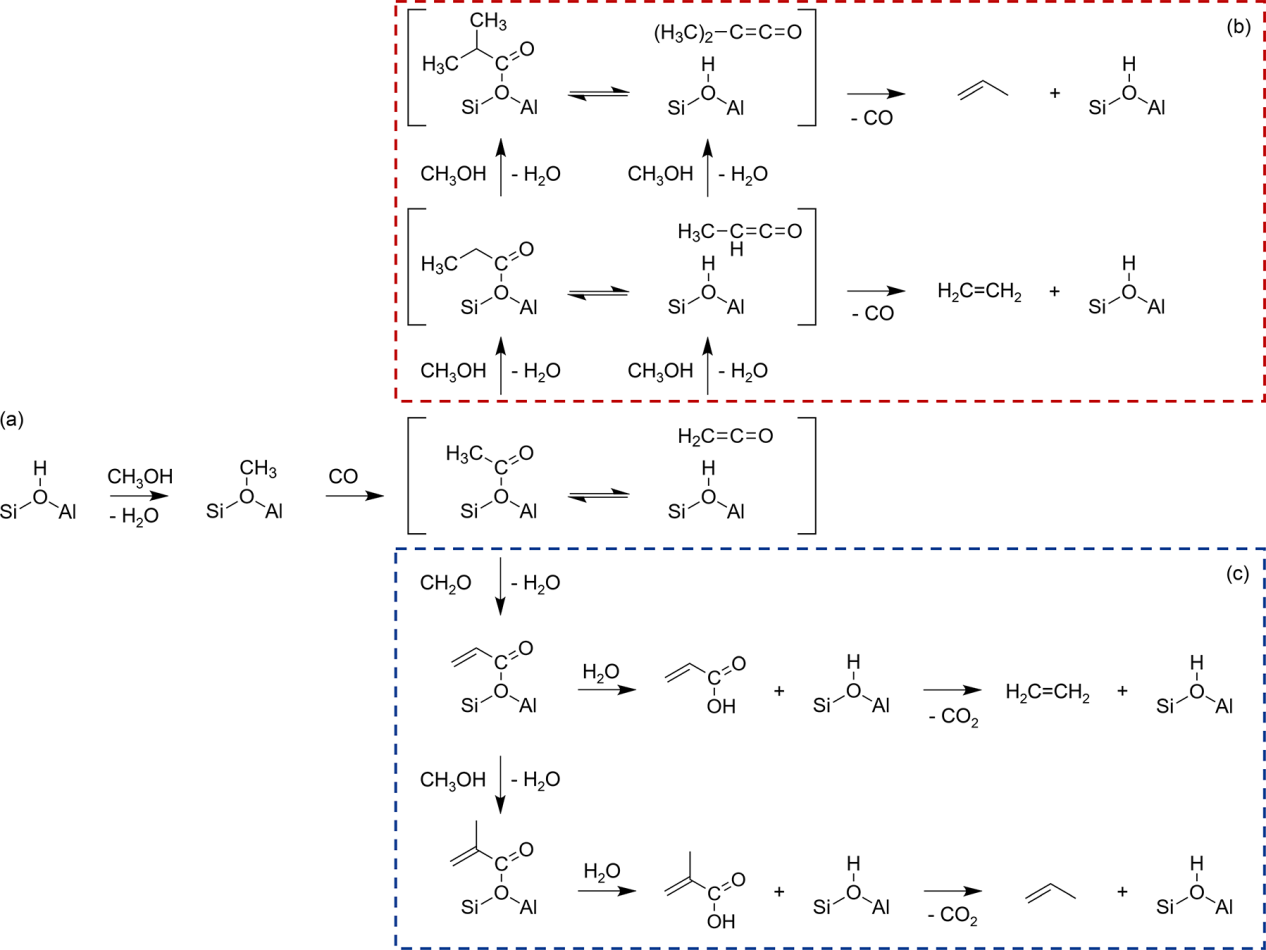
Proposed mechanistic routes to the first direct C–C bond formation and first olefin formation via Koch carbonylation. (a) The surface methoxy group undergoes carbonylation to form a surface acetyl species or ketene, which is the first C–C-containing intermediate.^104,105^ (b) The surface acetyl group or ketene could then propagate with methanol and decarbonylate to form the first ethene or propene.^106,107,109^ (c) The surface acetyl group could also react with formaldehyde to form unsaturated carboxylic acid intermediates, which decarboxylate to form the first ethene or propene.^102^ A propadienone resonance structure might exist for Path (c), analogous to the methyl-substituted ketenes in Path (b).

While early studies agree that the MTH reaction occurs via the dual hydrocarbon pool mechanism, no consensus was reached on the formation of the initial C–C bond, and this received much attention in the past decade. Several mechanistic routes pointing to the direct C–C bond formation with key intermediates such as carbene, methane–formaldehyde, methoxymethyl cation, methyleneoxy cation and acetyl have been suggested.^42^ To highlight the role of CO in the initiation step, the Koch carbonylation mechanism is discussed in more detail here. According to the Koch carbonylation mechanism, methanol is first dehydrogenated to formaldehyde, which is then oxidized to CO and subsequently carbonylated with methoxy species to form acetic acid or methyl acetate (Figure 5). Likewise, methanol dehydrogenation has been found to constitute the first step in the conversion of olefins to aromatic products, and aromatic products to polyaromatic coke precursors (vide infra, Figure 4).^89,90,96^ As formaldehyde is proposed to be the origin of CO in the MTH reaction, a deeper understanding of the significance of both formaldehyde and CO is relevant for the discussion on the integration of MTH and CO_x_ hydrogenation.

Formaldehyde is a key intermediate in two of the proposed initiation mechanisms, namely, the methane–formaldehyde and the Koch carbonylation routes. In the first case, formaldehyde is not decomposed to CO but instead reacts directly with methane to form the first C–C bond.^97^ Methane and formaldehyde could be formed from methanol disproportionation over Brønsted acid sites (BAS), meaning their production rates correlate positively to methanol partial pressure.^98^ Depending on the coreactant (none, methanol/DME, olefin), H_2_, CH_4_, or paraffins are coproduced with the formaldehyde (Figure 4). Co-reaction of methanol with olefins to form paraffins and formaldehyde is in accordance with the hydrogen transfer reactions described previously in this section. A positive correlation has been found between the abundance of hydrogen transfer products and the concentration of Lewis acid sites in the zeolite.^96^ This is different from the rates of alkene and arene methylation reactions, which have been found to correlate positively with the concentration of Brønsted acid sites.

In addition to its key role in the initial C–C bond formation, formaldehyde is responsible for polyene and aromatics formation, which leads to coking and catalyst deactivation.^99,100^ Specifically, formaldehyde has been demonstrated through cofeeding and isotopic labeling (^13^C-formaldehyde) experiments to react with olefins via Prins condensation into dienes and aromatics.^101,102^ Hence, decomposition of formaldehyde to CO and H_2_ by a Y_2_O_3_ catalyst has been attempted as a strategy to improve catalyst lifetime.^100,103^ The influence of proximity between the CHA zeolite/zeotype (SAPO-34 or SSZ-13) and Y_2_O_3_ was studied at 400 °C, 1 bar, 0.12 bar methanol, CHA/Y_2_O_3_ = 3, and the most intimate mixing, i.e., intrapellet at 1 μm proximity, improved the catalyst lifetime by 4 times.^103^ In the direct conversion of CO_x_ to hydrocarbons, the partial pressure of CO would expectedly be much higher than the conventional MTH reaction, and the formaldehyde reactions may be affected.

Moving from the origin of CO to its significance, it is proposed that CO reacts with surface methoxy species to form the first C–C bond via Koch carbonylation. The Lercher group first identified methyl acetate and acetic acid as the first C–C bond containing intermediates from a ZSM-5 catalyst in the early stages of MTH.^104^ Using an online MS, methyl acetate and acetic acid were detected at the lower temperatures of 200 to 300 °C (1 bar) and were then converted to olefins at higher temperatures. Switching the carrier gas of methanol from He to CO, the olefin formation rate increased, and the existence of acetyl surface groups was confirmed with in situ IR spectroscopy. Such promotional effects of CO were not observed in previous literature, and the authors attributed this to a higher partial pressure of H_2_O, which inhibited the carbonylation reaction. The acetic acid or methyl acetate is proposed to then react with formaldehyde into unsaturated carboxylate or carboxylic acid, respectively, which decarboxylates into the first olefin (Figure 5).^102^ Independently, the Weckhuysen group employed advanced UV–vis and solid-state NMR spectroscopy techniques to identify methyl acetate as the first C–C-bond-containing intermediate to be formed in a SAPO-34 catalyst at 400 °C and 1 bar.^105,106^ Contrary to the mechanism above, their experimental insights supported the theoretical results and mechanism put forward by Plessow and Studt, in which ketene or methyl acetate reacts with a surface methoxy specie followed by decarbonylation to form the first olefin (Figure 5).^107−109^ In the latter mechanism, CO acts as a cocatalyst and is not consumed (Path (b)). In the former mechanism (Path (c)), CO is indirectly converted to CO_2_. A hint in favor of Path (b), in which multiple “first C–C bonds” may be formed from a single CO molecule, arises from prior studies in which the coke formed in the MTH initiation zone of a ZSM-23 catalyst contained close to 2 orders of magnitude more C than the CH_4_ formed in the same zone.^110^ While the exact mechanism to produce the first olefin still needs to be resolved, the latest work by the Bordiga group observed the presence of free vibrating CO throughout the MTO reaction catalyzed by MAPO-18s and suggested that CO may play a role beyond the first C–C bond initiation.^111^ Zeolite-catalyzed carbonylation mechanisms are described in more detail in Sections 4.1 and 4.2. For a more extensive review of hydrocarbon pool initiation mechanisms, we refer the reader to the recent review by Yarulina et al.^42^

Besides CO, water is another molecule of interest in the MTH process, which is relevant for the integrated process from CO_x_ to hydrocarbons. Water is well known to compete with methanol and DME for adsorption at the Brønsted acid sites,^112^ and for forming protonated water clusters at high water contents, thereby slowing down methanol conversion.^113−115^ Water coverage of the BAS may be reduced by working at high temperature, but may also facilitate hydration of the Si–O–Al bond, leading to extra-framework alumina formation and permanent deactivation of the catalyst.^116−118^ Hence, the temperature must be chosen with care. On the other hand, water has also been shown to be beneficial for catalytic stability, so it is used as a strategy to improve the lifetime of SAPO-34 catalysts at typical MTO conditions of 400 °C and 1 bar.^79,112,119^ A probable reason is the hydrolysis of formaldehyde, which is a known coke precursor, thereby reducing the coking rate.^120^ Another reason may be the reduction of relative reaction vs diffusion rates (Thiele modulus) resulting from unselective quenching of hydrocarbon pool species formation and conversion reactions by water adsorption on BAS, as recently reported for a SAPO-18 catalyst.^121^

**Figure 6:**
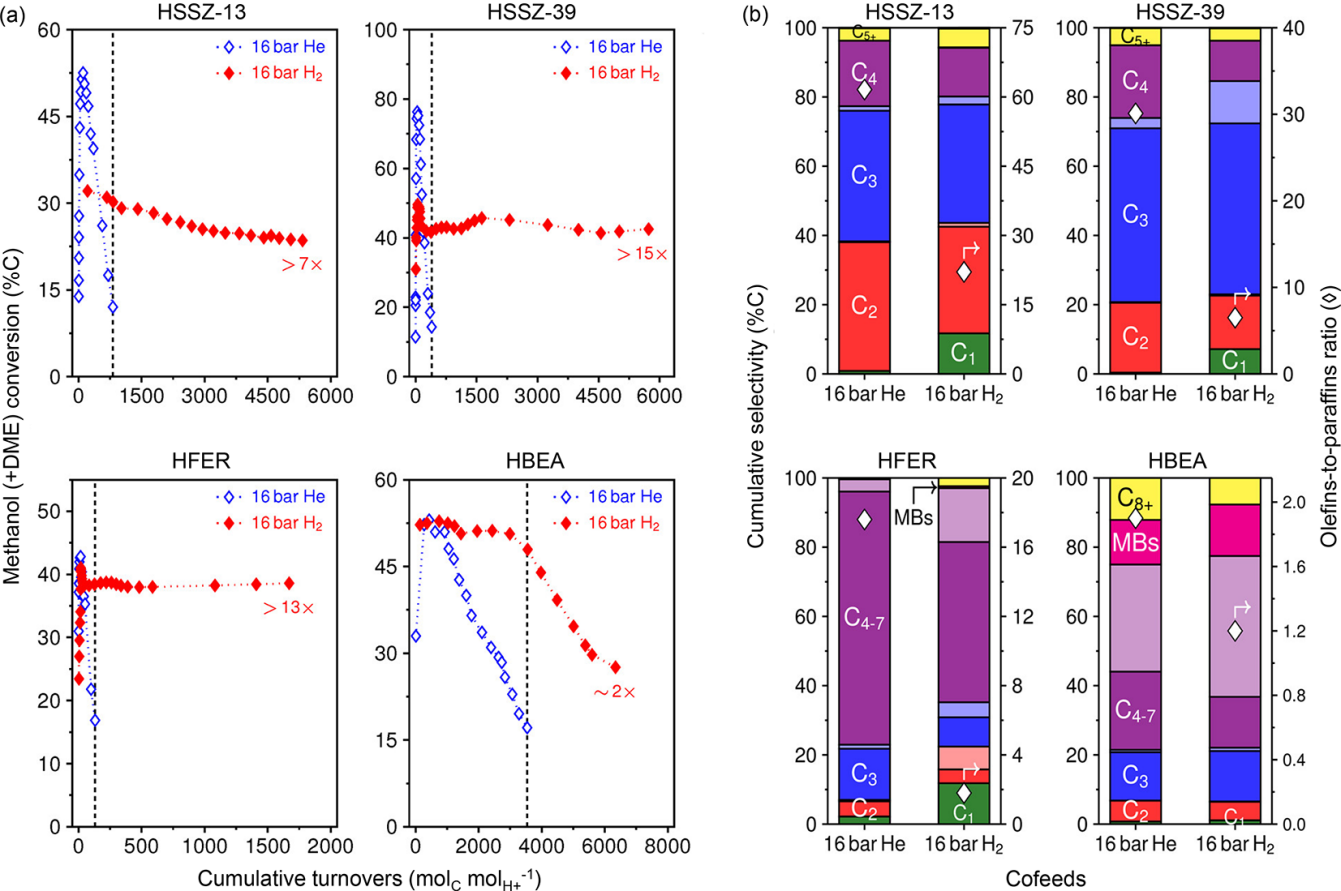
(a) Methanol conversion profiles versus cumulative turnovers and (b) cumulative selectivity (left ordinate) and the overall olefins-to-paraffins ratio (right ordinate) in the effluent stream observed during methanol feeds with and without H_2_ cofeeds over HSSZ-13, HSSZ-39, HFER, and HBEA. Reaction conditions: 350 °C, 0.005 bar methanol, 16 bar He or H_2_ cofeed, 350 [HSSZ-13]; 114 [HSSZ-39]; 61 [HFER]; 89 [HBEA] mol_C_ (mol_H^+^_·ks)^−1^. The dark- and light-shaded bars in (b) represent the olefinic and paraffinic forms, respectively, of the respective carbon group listed in the dark bars; “MBs” represents methyl-substituted benzenes. Reproduced from ref (122).

In order to close the process gap between the MTH process and the direct process for CO_x_ hydrogenation to hydrocarbons, a “next-generation” MTH catalyst and process has to operate at elevated pressures and lower temperatures in a CO/CO_2_/H_2_/H_2_O environment. Thus, recent breakthroughs in the MTH process have focused on cofeeding these gases at elevated pressures. A main gas component, H_2_, has been shown to be favorable for zeolite performance at elevated pressures of 16 to 30 bar, by drastically reducing catalyst deactivation by coke formation.^123−127^ From Figure 6a, the lifetime of four zeolites (SSZ-13, SSZ-39, FER, BEA) improved when 16 bar of H_2_ was cofed with methanol.^122^ In particular, H_2_ was demonstrated to hydrogenate the coke precursors, formaldehyde, and 1,3-butadiene. This effect has a huge impact on process parameters, especially regeneration cycles for 8-ring cavity-window structured catalysts. Interesting to note, regeneration of deactivated ZSM-5 catalysts was attempted with H_2_ at 20 bar and 480 or 550 °C to yield light hydrocarbons and aromatics.^128^ Unfortunately, most studies pointed to hydrogenation even of olefins to form paraffinic products, as represented in Figure 6b.

Other gases present in the CO_x_ hydrogenation/tandem reactor, such as CO and H_2_O, may then be employed to counteract the hydrogenation of olefins during the MTH reaction due to high pressure H_2_ cofeeding.^126,127^ At 450 °C and 40 bar, the cofeeding of methanol, H_2_, and H_2_O increased the methanol handling capacity more than 200 times in comparison to typical MTH conditions.^126^ However, as mentioned earlier about the H_2_O influences, this lifetime improvement may be yet at the expense of catalyst degradation. Importantly, in another recent contribution, CO was found to have a very strong effect, suppressing olefin hydrogenation while maintaining the favorable turn-over number effect of cofed H_2_, especially for M(II)-substituted AlPO-18.^127^ Additional experiments where ethene was cofed with surplus H_2_/N_2_ or H_2_/CO over SAPO-18 confirmed that CO addition suppresses ethene hydrogenation.

The mechanism of product formation from carbonylated intermediates is not yet fully resolved. It could proceed by decarbonylation/decarboxylation (as proposed for the first C–C bond formation, Figure 5) or by dehydration. Dehydration seems plausible in the contribution from Liu and co-workers, where they cofed CO with methanol with CO/methanol ratios in the range of 20–113 at 400 °C, 40–50 bar total pressure, over H-ZSM-5 and observed ^13^C in methylcyclopentenone products after ^13^CO/^12^C-methanol cofeed experiments.^129^ Notably, very little ^13^C was found in propene and some more in propane. The thermodynamic drive of aromatic formation may be a reason for the dehydration instead of decarboxylation in longer C_n_ chains/cyclic compounds. A further interesting point of this contribution is that CO seems to partly suppress the hydrogen transfer route from alkenes to aromatics, leading to much less alkane formation. This result is in line with Xie et al.^127^ Recently, Shi et al. cofed H_2_ (0–12 bar), CO (0–8 bar), and/or H_2_O (0–4.5 bar) with methanol (1 or 10 kPa) over H-SAPO-34 at 400 °C.^130^ The results were in general agreement with the results described above. Furthermore, they found that the influence of CO on product selectivity vanished at the highest methanol pressure. This observation was ascribed to the higher concentration of organic compounds in the catalyst cavities for higher methanol concentrations, thereby outcompeting the reaction of CO with methoxy species. H_2_O addition was found to decrease the formation rate and steady state concentration of C_2+_ compounds in the catalyst, without influencing product selectivity, in line with Valecillos et al.^121^

### 2.2 Integration of the MTH Reactions with CO_x_ Hydrogenation

The industrial methanol synthesis catalyst (CZA) consists of Cu as the active element, and ZnO and Al_2_O_3_ as structural and electronic promoters.^131^ The operating conditions are 200 to 300 °C, 50 to 100 bar, with a gaseous feed of CO/CO_2_/H_2_. The feed composition is determined by the stoichiometry required for methanol synthesis, which is defined as M = (H_2_ – CO_2_)/(CO + CO_2_) ≈ 2. The discovery and historical development of the industrial Cu-based methanol synthesis catalysts is described in the review by Waugh.^132^ For recent progress on industrial methanol synthesis from CO/CO_2_/H_2_, the review by Sehested on catalyst development and the review by Bozzano and Manenti on reactor technologies are recommended.^131,133^ A current focus is the conversion of CO_2_ and H_2_ to methanol in which the Cu-based catalysts face the challenge of selectivity and stability due to oxidation and sintering. At 200 °C, 30 bar, H_2_/CO_2_ = 3, and GHSV = 9000 h^–1^, the space time yield of methanol over a commercial CZA catalyst decreased by 34.5% after 720 h time-on-stream and XPS results of the spent catalyst revealed the oxidation of metallic Cu to Cu^2+^.^134^ From XRD and N_2_O adsorption, Cu particle size did not change, but ZnO dispersion decreased. Hence, a cause for the decrease in methanol time yield was concluded to be oxidation of metallic Cu instead of sintering of Cu nanoparticles. On the other hand, several studies at harsher conditions, e.g., 250 °C and 50 bar, correlated the decrease in methanol space time yield with steam-induced Cu nanoparticle growth.^135−137^ Currently, oxide-based bulk catalysts are increasingly explored, plausibly inspired by the first-generation ZnCrO_x_ catalysts developed by BASF in 1923.^28,40^

Methanol-mediated conversion of CO_x_ and H_2_ to hydrocarbons is covered in several recent contributions and review articles.^3,18,20^ Focusing on the zeolite function, most contributions apply the commercial MTH catalysts, H-ZSM-5 and H-SAPO-34, mixed with metal–support or mixed metal oxide CO_x_ hydrogenation catalysts. Reaction temperature is typically 350 °C and higher, in order to thermodynamically favor dealkylation of aromatic intermediates,^138,139^ as well as cracking of longer-chain alkenes to form lighter analogues.^140^ Benzene, toluene, and xylenes (BTX) and light alkenes, propene in particular, are the most valuable products. Under conventional MTH conditions (350 to 500 °C, 1 bar, 13 kPa methanol in inert flow), high alkene yields may be obtained over a wide range of zeolites, including H-ZSM-5 and H-SAPO-34, especially at 400 °C. Higher temperatures favor hydrogen transfer reactions and yield more aromatic and paraffinic products.^141^

The applications of zeolites in C_1_ conversion have been reviewed in a broad context that includes FTS and methanol-mediated processes,^38,142^ and that is different from this review, which focuses on the oxygenate-mediated processes. The effects of zeolite topology, crystal size, acidity, and proximity of the two catalytic functions are summarized previously, so only the key developments together with our viewpoints will be highlighted. A main issue related to the methanol-mediated CO_x_ hydrogenation process is the extent to which the overall product selectivity is influenced by altered reaction conditions and products formed on the methanol synthesis catalyst, compared to MTO. In qualitative terms, a recent study of the methanol-mediated CO hydrogenation process where ZnAlO_x_ was combined with various SAPO zeotypes showed that the general trend of zeolite shape selectivity observed in prior MTO studies prevail:^46,143^ 8-ring/12-ring window-cavity structures yield C_2_–C_4_ olefins and paraffins as main products, while 1D 10-ring structures yield C_4_–C_6_ olefins and paraffins as main products. 1D and 3D 12-ring structures yield C_3_–C_5+_ as main products, with a higher paraffin-to-olefin ratio than the 8- and 10-ring pore/window structures, due to substantial formation of aromatic products.

**Figure 7:**
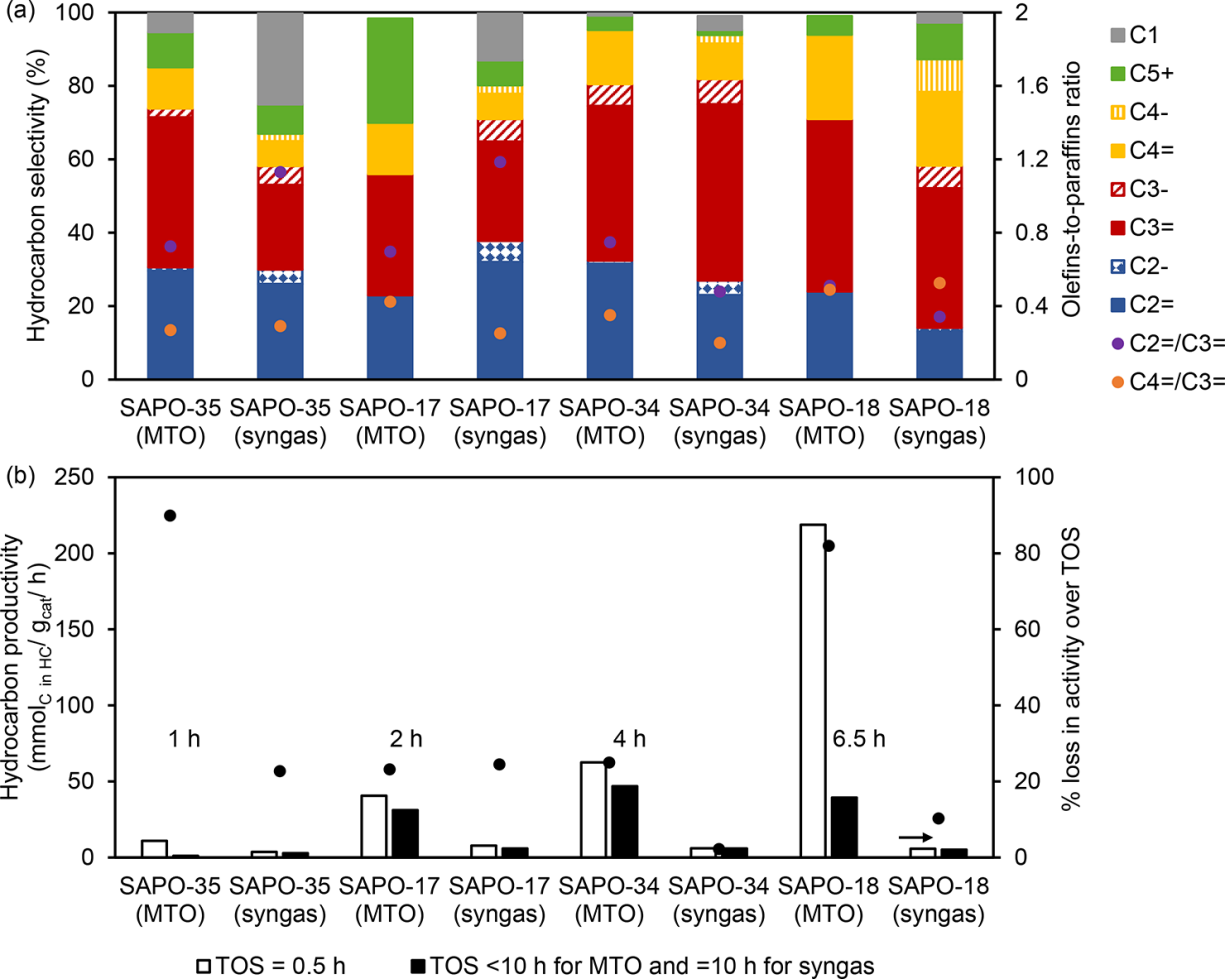
(a) Hydrocarbon product distribution and (b) hydrocarbon productivity over TOS attained using 8-ring zeotypes during MTO or syngas conversion at 400 °C. Data sets for SAPO-35 (MTO) from ref (144), SAPO-17 (MTO) from ref (145), SAPO-34 (MTO) from ref (146), SAPO-18 (MTO) from ref (147), and all SAPOs (syngas) from ref (143).

A quantitative comparison of the hydrocarbon selectivity attained with four 8-ring SAPOs under MTO or syngas conversion conditions is illustrated in Figure 7. The data sets for the SAPOs tested for MTO were selected based on the synthesis protocols used to prepare the SAPOs tested for the conversion of syngas.^143−148^ Due to the 8-ring structures, C_2_–C_4_ hydrocarbon selectivity was at least 67% in all cases. Furthermore, the distribution of ethene, propene, and butenes produced in syngas conversion fit well to the concept of the “cage-defining ring size” proposed by the Davis group for MTO.^148^ Specifically, SAPO-35, SAPO-17, and SAPO-34 produced more ethene and propene, while SAPO-18 produced more propene and butenes. In the conversion of syngas, more methane and paraffins were produced than those in the MTO reaction. Methane selectivity in MTO is typically lower than 2%, except during initiation and late deactivation stages.^144,149^ Both stages are characterized by low concentrations of olefins and aromatic products, which favor methanol–methoxy reactions to form methane and formaldehyde (vide ultra). Although methane has been assigned as a possible product from the methanol synthesis catalysts, it may not be the reason in this case since the same ZnAlO_x_ catalyst was used with the different SAPOs. The methane selectivity also appeared to increase with increasing “cage-defining ring size” of the SAPOs during syngas conversion. Another possible reason for the higher selectivity toward paraffins could be the hydrogenation of the olefins due to the high-pressure H_2_ environment, as discussed previously in the cofeeding of high-pressure H_2_ in the MTO process.^127^ Hydrocarbon formation rates (on a C_1_ basis) and productivity numbers after 1 and 10 h on stream for MTO and tandem operation of the four catalyst structures are compared in Figure 7b. The initial rates are typically 1–2 orders of magnitude higher for MTO operation but rapidly decline. Instead, conversion rates under tandem operation are slow and rather stable over the test period. While the test duration was limited to 10 h in the examples of Figure 7, the superior time-on-stream (TOS) stability of the tandem process is confirmed by other literature studies, in which the tandem reaction was run for extended periods.^3^ A recent example is the CO_2_ conversion over a GaZrO_x_ + SSZ-13 catalyst at 350 °C, 30 bar, H_2_/CO_2_ = 6, in which the C-based hydrocarbon production rate was initially 1.3 mmol/g_cat_·h, and still 0.9 mmol/g_cat_·h after 500 h on stream.^150^ The conversion capacity of this system (>550 mmol/g_cat_) is therefore higher than most of the MTO examples in Figure 7.

As outlined by Tian et al.,^36^ the rapid deactivation of MTO catalysts imposes the use of a circulating fluidized-bed reactor and regenerator configuration similar to a fluid catalytic cracking (FCC) riser reactor concept for rapid and frequent removal of coke to regenerate catalysts. Attrition strength is therefore an important parameter of MTO catalyst design, due to erosion in the fluidized bed, as well as strain and chemical degradation of the zeolite/zeotype lattice imposed by frequent coke formation–regeneration cycles. Efforts targeting improved long-term catalyst cycle stability and carbon yields include regeneration in H_2_ and alkanes,^151^ precoking and water cofeed,^119^ and transformation of coke species to active carbonaceous intermediates via steam cracking.^152^ While those approaches may be useful even for catalyst regeneration after long cycle times, the fluidized-bed-specific catalyst attrition strength is less of an issue for tandem catalyst operation, for which cycle times are sufficiently long for fixed bed operation. On the other hand, the low product formation rates observed under tandem operation will impact reactor size and recycle ratios.

Another 8-ring zeolite, RUB-13, was recently investigated for the direct conversion of CO_2_.^153^ RUB-13 has a slightly wider window size than SAPO-34, and it shifted product selectivity toward C_3_ and C_4_ olefins rather than ethene. A total C_2_–C_4_ olefin selectivity in hydrocarbons of 65–83% at a CO_2_ conversion of 10–16%, of which > 90% was C_3_–C_4_, was observed over ZnZrO_x_/RUB-13. ZrCrO_x_/RUB-13 showed the highest ethene selectivity among the four tandem catalysts. This result was ascribed to the higher strong acid content and proportion of this catalyst, promoting the aromatic-based cycle to produce more ethene.

**Figure 8:**
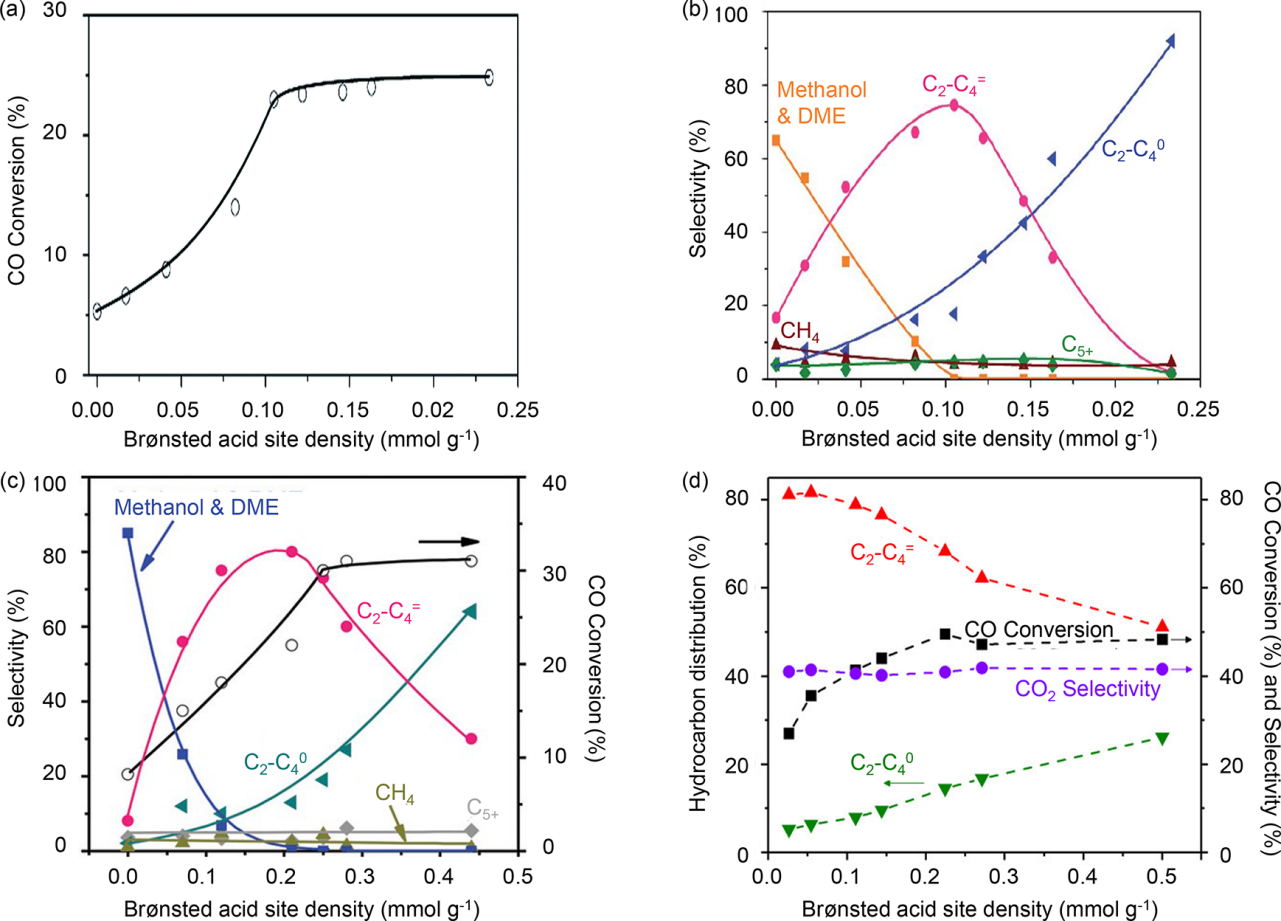
Influence of zeotype acidity on the direct conversion of syngas to lower olefins. (a) CO conversion and (b) hydrocarbon selectivity as a function of BAS density in SSZ-13. Reaction conditions: ZnZrO_x_ as methanol synthesis catalyst, 400 °C, 30 bar, H_2_/CO = 2. Reproduced from ref (154). (c) Hydrocarbon selectivity as a function of the BAS density in SAPO-34. Reaction conditions: ZnAlO_x_ as methanol synthesis catalyst, 400 °C, 30 bar, H_2_/CO = 2. Reproduced from ref (155). (d) Hydrocarbon selectivity as a function of the BAS in SAPO-18. Reaction conditions: ZnCrO_x_ as methanol synthesis catalyst, 400 °C, 40 bar, H_2_/CO = 2.5. Reproduced from ref (156).

In addition to the partial pressures of the reactants and products, the extent of olefin hydrogenation has been reported to depend on the acidity of the zeolite/zeotype.^154−156^ From Figure 8a and 8b, the density of BAS in SSZ-13 was revealed to influence CO conversion and hydrocarbon product selectivity in the conversion of syngas catalyzed by ZnZrO_x_/SSZ-13, at 400 °C, 30 bar, H_2_/CO = 2.^154^ When the BAS density was lower than 0.1 mmol/g, an increase in BAS corresponded to an increase in CO conversion and a decrease in methanol and DME products. The increase in BAS led to a higher conversion and rapid removal of methanol and DME, hence, creating a thermodynamic driving force to increase CO conversion. The C_2_–C_4_ olefin/paraffin ratios remained stable between 4 and 5, possibly due to lack of vacant BAS for olefin adsorption. When the BAS density was higher than 0.1 mmol/g, complete conversion of methanol and DME was attained, and the CO conversion did not increase beyond 25%. The availability of BAS led to olefin hydrogenation, so the C_2_–C_4_ olefin/paraffin ratios decreased to 0.03 at BAS = 0.23 mmol/g. These effects of the BAS density were also observed for other catalytic systems, such as ZnAlO_x_/SAPO-34 tested at 400 °C, 30 bar, H_2_/CO = 2 (Figure 8c) and ZnCrO_x_/SAPO-18 tested at 400 °C, 40 bar, H_2_/CO = 2.5 (Figure 8d).^155,156^ In the study on the role of SAPO-18 acidity, the higher BAS density was also found to increase the C_3_/C_2_ ratio, and the authors attributed this to the secondary reactions of ethene, which decreased ethene selectivity.^156^

The considerations for zeolite/zeotype acidity in the direct CO_x_ hydrogenation to hydrocarbons via methanol are unlike in the MTH processes. In MTO, the SAPO-34 zeotype is preferred over its isostructural SSZ-13 zeolite^36,37^ because the stronger acidic strength of the SSZ-13 often results in faster deactivation and more paraffins.^157,158^ This preference is not obvious in direct CO_x_ hydrogenation, as inferred from Figure 8b and 8c. It should be noted, though, that even MTO conditions may be tuned to favor SSZ-13 over SAPO-34 as MTO catalyst.^139^ Thus, targeted studies on the influences of zeotype/zeolite acidity, including acidic strength, BAS and LAS, in the oxygenates-mediated CO_x_ conversion would be needed to unravel deeper insights.

**Figure 9:**
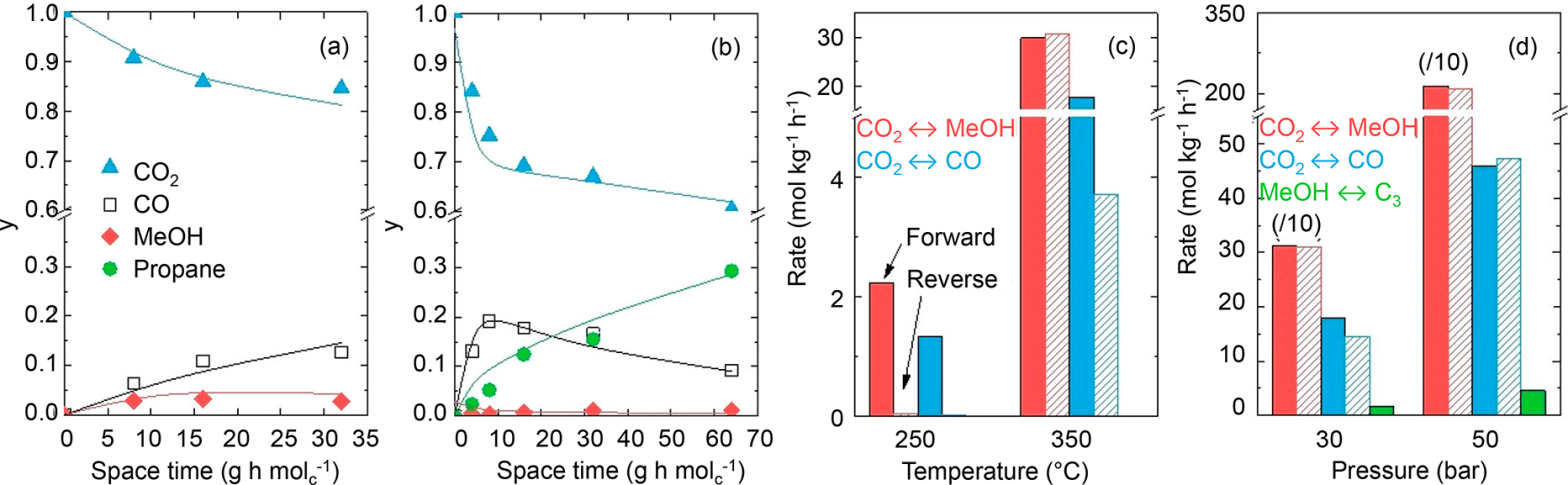
Experimental data fitting of (a) CO_2_ to methanol over the PdZn/ZrO_2_ catalyst at 300 °C and 30 bar and (b) CO_2_ to propane over the PdZn/ZrO_2_+SAPO-34 system at 350 °C and 50 bar. (c) Comparison of reaction rates for methanol and CO formation over the PdZn/ZrO_2_ catalyst at 250 and 350 °C and 30 bar (12,000 mL/g_cat_/h), and (d) influence of propane formation on these rates over the PdZn/ZrO_2_+SAPO-34 system at 350 °C and 30 and 50 bar (6000 mL/g_cat_/h). Reproduced from ref (159).

Considering the kinetics of the tandem reaction from CO_2_ and H_2_ to hydrocarbons, a recent study of a mixture of PdZn/ZrO_2_ and H-SAPO-34 catalysts showed that the reactions to form methanol and CO from CO_2_ rapidly reached equilibrium over the PdZn/ZrO_2_ catalyst, while methanol conversion to form hydrocarbons was the rate-limiting step of the reaction (Figure 9).^159^ In this particular case, the hydrogenating function of the PdZn alloy is so strong that only paraffinic products were formed, with exceptional selectivity toward propane. Overall, this study suggests that the hydrocarbon formation rate remains a challenge in the tandem reaction scheme. The leveling out of CO conversion versus BAS density in Figure 8a points in the same direction. In a follow-up study, Cordero-Lanzac et al. demonstrated an inverse first order correlation between the water content and the rate of methanol conversion to hydrocarbons over the PdZn/ZrO_2_+H-SAPO-34 catalyst.^160^

Beyond effluent selectivity, a general feature of tandem catalysts is their much superior resistance to coking compared to MTO operation conditions (cf. Figure 7b). The process conditions and origin of the improvement in catalytic stability were investigated by Nieskens et al. through a direct comparison of the lifetime and coke species of SAPO-34 in MTO and syngas conversion.^124^ Notably, different hydrocarbon species were extracted from the spent MTO and syngas catalysts. From the spent SAPO-34 used in converting syngas at 400 °C, 15 bar, and H_2_/CO = 3, mainly smaller aromatic molecules, such as benzene, toluene, xylenes, and naphthalenes, were found. From the spent SAPO-34 used in converting methanol at 400 °C, 1 bar, and WHSV methanol = 1.3 h^–1^, the trapped coke species were predominantly anthracenes and pyrenes. The differences were attributed to the presence of H_2_ in the feed, in particular, the H_2_/methanol partial pressures. Furthermore, the reaction temperature had to be higher than 300 °C for high-pressure H_2_ to suppress hard coke formation. These results are in agreement with the high-pressure cofeed studies in MTO (Section 2.1), whereby the H_2_ content of the feed and effluent gas were observed to hinder the hydrogen transfer reactions that precede aromatics and polyaromatics formation in zeolites/zeotypes.

From the mechanistic perspective, the general understanding is that methanol conversion to hydrocarbons in the direct CO_x_ hydrogenation processes occurs via the conventional “dual cycle” reaction mechanism for MTH (Figure 4). However, Hou and co-workers recently proposed a “triple cycle” instead.^161^ A quasi-in situ, solid-state NMR and online GC/GC-MS analysis methodology was implemented to monitor the direct syngas conversion during start-up and steady state operation. ZnAlO_x_ was mixed with either ZSM-5 (Si/Al = 82) or ZSM-5 (Si/Al = 16) and tested at 300 °C, 25 bar, and H_2_/CO = 1. Excluding the 45% CO_2_ selectivity, the tandem catalyst with more BAS showed 80% selectivity toward aromatics, while the other tandem catalyst was more selective toward paraffins (∼60% C_2_–C_4_, ∼20% C_5+_). These hydrocarbon product distributions were different from the MTH control experiments as the aromatics selectivity of both catalysts were only ∼35%. According to the solid state NMR analysis, formate, methanol, and DME were observed after 30 s of reaction, while acetates and carboxylates were detected after 2 min of reaction. The acetates and carboxylates were proposed to be formed from olefins/alkoxyls and CO via Koch carbonylation, and they were presented as evidence for the oxygenate-based cycle.

The new oxygenate-based cycle bears a resemblance to the reaction pathway for the first olefin formation in MTH (Figure 5), with the same intermediate (acetate) being observed in both cases. The three main differences between the MTO and syngas case is first, that the CO/methanol ratio is dramatically different between the two cases. In the syngas case, it is typically > 10^2^ (based on effluent analysis), while in the MTO case, it is likely < 10^–2^ (when estimated from the CH_4_ yield). This difference will dramatically shift the relative rates of surface methoxy reactions with either CO to form acetyl/ketene/acetate, or olefins/arenes to form higher olefins/methylated aromatic products. According to Figure 5 Path (b), CO acts as a cocatalyst of methanol conversion in the zeolite/zeotype, just like olefins and monocyclic aromatics–the difference between the cycles being that CO is not an autocatalytic species. Prior studies by the Liu group (vide ultra, Section 2.1) showed that ^13^C-CO cofed with ^12^C-methanol over H-ZSM-5 yielded ^13^C-containing aromatic products, while at the same time suppressing paraffin formation, which is the trademark of aromatics formation by hydrogen transfer reactions in MTH.^129^ Those results are in line with the “triple-cycle” reaction concept and point to detailed studies of the relative rates of carbonylation versus methylation reactions as a highly interesting topic of further study. The second main difference between the MTO and syngas case is the H_2_/methanol ratio, which is even more dramatic than the CO/methanol ratio difference. Prior cofeed studies (vide ultra, Section 2.1) showed how hydrogen cofed with methanol suppresses coke formation by hydrogenation of coke precursors, and how H_2_/CO cofed with methanol forms a well-balanced pool of retained species that produces olefins rather than paraffins, yet with limited deactivation due to coke formation. Again, follow-up studies to unravel individual and combined effects would be highly valuable for future catalyst optimization by design. The third main difference between the syngas and MTO cases is the (de-)hydrogenation function of the methanol-producing catalyst. As pointed out by Hou and co-workers, mixing the two catalyst functions enables acetate/acetic acid (and higher analogues) to diffuse out of the zeolite/zeotype and be hydrogenated to ethene on the methanol-producing catalyst, hence accelerating the final steps of the MTO initiation reactions to first C–C bond formation.^161^ Together, the recent mechanistic insights in tandem reactions create a novel playground for fundamental as well as more application-oriented studies toward a next-generation tandem process.

## 3 Dimethyl-Ether-Mediated CO_x_ Conversion to Hydrocarbons

With one exception (vide infra, Section 3.3), all contributions cited in this review article report or presume that dimethyl ether is formed via methanol dehydration. As will be seen in Sections 3.2–3.3, the dehydration site may be present in the CO_2_ hydrogenation catalyst, in the form of a support material or a binder added during catalyst formulation, or in the hydrocarbon-forming zeolite/zeotype. First, however, in Section 3.1, we focus on the conversion of DME to hydrocarbons.

### 3.1 Dimethyl Ether to Hydrocarbons (DTH)

In the first publication in 1977 on MTH using ZSM-5 at 371 °C, Chang and Silvestri from Mobil proposed that the conversion of methanol to gasoline proceeded via partial methanol dehydration to DME, which resulted in an equilibrated feed mixture.^81^ The first MTG plant was commercialized by Mobil in New Zealand in 1985, and in the process flowsheets, methanol was dehydrated to DME in the first reactor and the effluent was then converted to gasoline in the second reactor.^162^ This dual reactor flowsheet removed heat produced during methanol dehydration and allowed better heat control during gasoline production from the methanol/DME/H_2_O equilibrated feed mixture. Among the subsequent MTH industrial developments, the utilization of DME as an intermediate was included in Topsøe’s TIGAS process, Lurgi’s MTP process, and Dalian Institute of Chemical Physics (DICP)’s DMTO process.^35,36,163−165^

DME and methanol were often treated as a single reactant feed, because they were considered to be in equilibrium under relevant MTH reaction conditions.^65,81,166^ This assumption appears to be valid because DTH and MTH were shown to follow the same dual hydrocarbon pool mechanism and to yield similar product distribution.^81,167−170^ Nonetheless, the mechanistic studies also concluded differences in the reaction rates, and these were highly dependent on zeolite/zeotype properties, such as topology, crystal size, and acidity.

In an early study of SAPO-34 at 425 °C, Holmen et al. used a tapered element oscillating microbalance (TEOM) to determine methanol and DME mass transfer limitations as a function of crystal sizes between 0.25 and 2.5 μm.^149^ DME diffusion was found to be more hindered than methanol, therefore resulting in slower overall reaction rates.^149^ In a similar study of SAPO-34 at 350 °C, 0.075 and 0.8 μm crystals were compared for DTH and MTH, and no diffusion limitations were observed.^169^ There were no significant differences between DTH and MTH, and the smaller crystals resulted in a longer lifetime. In a more recent study of SAPO-34 at 350 to 450 °C, Wang et al. noted a longer initial induction period with corresponding higher propylene selectivity for DTH than MTH.^170^ DME hydration was found to be unfavorable below 400 °C, which resulted in lower methanol partial pressure in the reactor and slower reaction rates for DTH. Despite the lower reactivity of DTH, it had the advantages of slower catalyst deactivation and higher propylene selectivity during the initial period, and this could potentially be exploited.

Moving on to ZSM-5, there is agreement that methylation rates in DTH are significantly faster than in MTH.^90,168,170,171^ Svelle et al. performed propylene and toluene methylation experiments using methanol and DME with ZSM-5 at 350 °C, and concluded that 1-butene formation rates were at least two times faster for DME than methanol over 0.02 to 0.08 bar (DME or methanol/propylene = 1 to 4).^168^ Castano et al. studied DTH and MTH using ZSM-5 at 400 °C and 1.5 bar, and also found faster kinetics of methylation for DTH in comparison to MTH.^171^ They reasoned that the faster methylation rate and lower water concentration present in DTH led to faster conversion of hydrocarbon pool intermediates. However, this also resulted in the formation of more methylated and condensed aromatics, i.e., coke, hence faster deactivation in DTH. In the direction of arene methylation in ZSM-5, Hibbitts et al. contributed a theoretical investigation on the implications of methanol and DME as methylation agents for all 20 arenes (C_6_ to C_12_) at 350 °C and 1 bar.^172^ They focused on possible confinement effects on the transition state structures and found that reorientations of the transition states could decrease energies up to 45 kJ/mol. This decrease in energy is more significant than the difference between the concerted or sequential methylation mechanism of 20 kJ/mol, implying that both mechanisms could take place. In addition, the energy barriers of methylation by methanol and DME are nearly identical, suggesting that both are equally capable of arene methylation.

**Figure 10:**
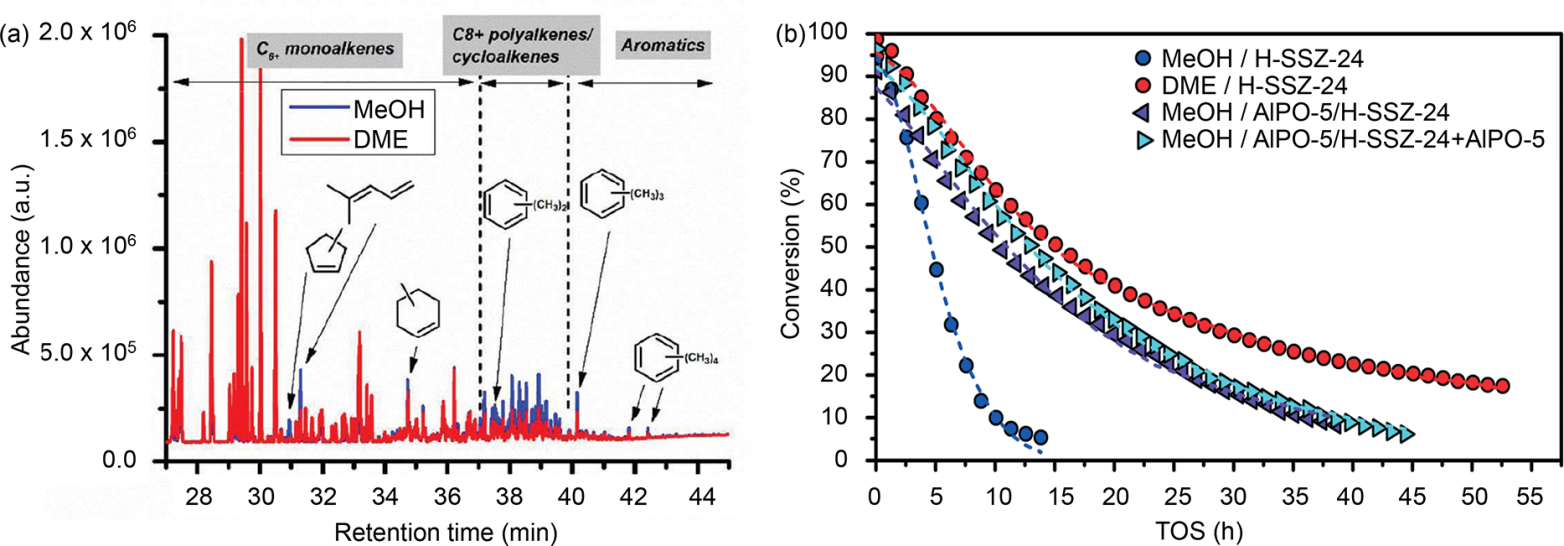
(a) GC chromatogram comparison of coreaction of 40 mbar isobutene with 40 mbar methanol (blue) and 40 mbar DME (red) over H-ZSM-5 at 350 °C. Reproduced from ref (89). (b) Deactivation profiles based on oxygenate conversion versus time-on-stream at 450 °C over SSZ-24 and AlPO-5/SSZ-24. Reproduced with permission from ref (99). Copyright 2017 Royal Society of Chemistry.

To unravel mechanistic differences and origins of DTH and MTH deactivation, Olsbye and co-workers designed a series of experiments which examined DME and methanol influences on individual reaction pathways in the arene cycle. First, the reactivity of DME and methanol with benzene was investigated at 250 to 350 °C, 1 bar, DME or methanol = benzene = 0.06 bar, and < 9% conversion (differential conditions).^90^ Higher methylation and dealkylation rates were observed with DME than with methanol, regardless of topology (MFI, ZSM-5 and AFI, and SSZ-24) and acidity (SSZ-24 and SAPO-5). Importantly, benzene methylation using DME resulted in the formation of toluene, polymethylbenzenes, and alkenes, while the use of methanol resulted in the formation of diphenylmethanes (DPMs) as well. Additional cofeed and isotopic labeling experiments revealed that methanol was first dehydrogenated to formaldehyde, which then reacted with two benzene molecules to form DPM and ultimately coke. Next, the role of formaldehyde was further investigated by cofeeding formaldehyde with methanol at 350 °C, 1 bar, 0.058 bar methanol, and 0.001 bar formaldehyde.^99^ The cofeeding of formaldehyde increased aromatics and ethylene formation, thus supporting the conclusions of the prior study. Based on the argument that methanol is a main source of formaldehyde, formaldehyde was demonstrated to be a critical factor for the faster deactivation during MTH than DTH. Finally, the reactivity of DME and methanol with isobutene was investigated at 350 °C, 1 bar, and DME or methanol = isobutene = 0.04 bar.^89^ Isobutene was used as a probe molecule to distinguish between methylation and H-transfer pathways, and H-transfer is critical for not only the arene cycle but also coke formation. In the case of MTH, methylation and H-transfer rates were equally competitive, whereas in the case of DTH, methylation rates were faster than H-transfer rates so the arene cycle and arene products were less dominating (Figure 10a). From these thorough cofeed studies on methanol vs DME, the origin of faster MTH deactivation was the formation of formaldehyde, which led to DPM, and the competitive rates of methylation and H-transfer. Thus, using DME rather than methanol as feed has clear benefits, and these studies provide mechanistic grounds on the use of a first reactor to dehydrate methanol to DME before a second reactor to produce hydrocarbons in the process flowsheet.

Martinez-Espin et al. further contested the hypothesis of methanol and DME being in equilibrium in the second reactor which produces hydrocarbons in the process.^99^ Notably, at relevant MTH conditions of 350 °C and 1 bar regardless of methanol, DME, or DME with water feeds, equilibrium was not reached with ZSM-5. This finding is also valid for other topologies with strong BAS, such as SSZ-24 (AFI), and it is due to the similar rates for methanol dehydration to DME and for methylation reactions over the strong BAS. The methanol and DME equilibrium could be established when weak acidic sites such as P–OH were present, as in the case of AlPO-5 addition to the catalyst bed (Figure 10b).

**Figure 11:**
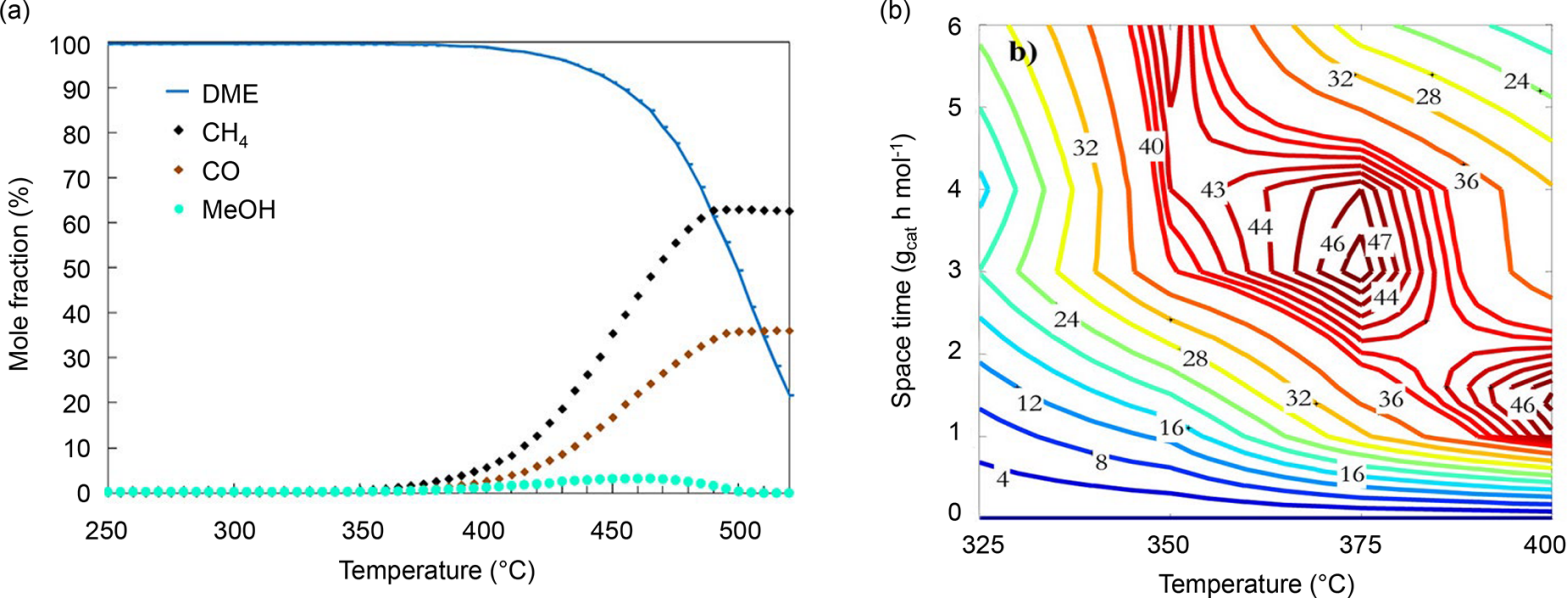
(a) Thermal cracking behavior of DME and (b) map of lower olefin yield at 1 atm of pure DME for a range of space times and temperatures. Reproduced from ref (174).

Besides the above studies which compared methanol and DME, it is noteworthy to then highlight relevant insights on DTH. Pérez-Uriarte et al. investigated the effects of zeolite topology using ZSM-5, SAPO-34, and SAPO-18 for DME conversion to lower olefins at 350 and 400 °C, and 1.5 bar pure DME.^173^ ZSM-5 showed the highest activity and stability, but surprisingly it showed the highest selectivity toward lower olefins (27% ethylene, 50% propylene, and 16% butenes) at low space time (∼10% conversion). As a follow-up, operating conditions were screened to optimize lower olefins yield using the ZSM-5 (Si/Al = 280) catalyst.^174^ Interestingly, DME was shown to undergo thermal cracking to give CO and methane when the reaction temperature was higher than 400 °C (Figure 11a). As a result, the selectivity and yield of lower olefins decreased at higher temperatures, as presented in Figure 11b. In Figure 11b, the red region of the heat map represented the highest yields of lower olefins, which was between 40 and 50%. The lower steam partial pressure in DTH was suggested to be the cause for higher reactivity but also faster deactivation, presumably due to the high DME partial pressure. Ortega and Kolb also studied the influence of process variables, specifically a temperature range between 325 and 375 °C and space time range between 0.008 and 0.040 h·kg_cat_/kg_DME_ using a ZSM-5 (Si/Al = 58) catalyst.^175^ Thorough data and kinetic analysis revealed that product selectivity was dependent on DME conversion and independent of reaction temperatures and space times. They further concluded that equilibrium was not reached between DME and methanol in this study, thereby supporting the earlier finding of Martinez-Espin et al.^99^

The Bhan group conceived several studies on olefins (i.e., ethylene, propylene, 1-butene, *trans*-butene, *cis*-butene, and isobutene) and aromatics (i.e., benzene, toluene, *para*-xylene, and *ortho*-xylene) methylation reactions using DME. Olefin methylation reactions using DME were performed with various zeolites (FER, MFI, MOR, and BEA) at low temperatures (<127 °C), high DME/olefin ratios (>15), and low conversions (<0.2%) to isolate primary reactions.^176,177^ Under such conditions, olefin methylation rates were determined to be independent of DME partial pressure and first-order in olefin partial pressure. Higher olefins and higher stabilities of the intermediate carbenium ions resulted in faster methylation rates. Aromatics methylation reactions using DME were carried out with ZSM-5 at similar conditions, and zero-order dependence on DME partial pressure was concluded.^178^ They further studied DTH over ZSM-5 at more relevant conditions of 275 °C, 1 bar, 0.7 bar DME, and ∼20% conversion.^179^ Co-feeding of 0.04 bar ^13^C-propylene with 0.7 bar ^12^C-DME suggested that C_5_–C_7_ olefins were formed mainly from methylation reactions, and these higher olefins subsequently cracked to form propylene. Co-feeding of 0.04 bar of ^13^C-toluene with 0.7 bar of ^12^C-DME showed that the higher aromatics originated from C_8+_ aliphatic cyclization reactions instead of toluene methylation reactions.

In conclusion, DTH has several advantages over MTH: The lower oxygen content of DME leads to a smaller exotherm in a DTH reactor compared to that in an MTH reactor. Furthermore, DME has a higher conversion rate for methylation of alkenes and arenes compared with methanol and is more selective for alkene/arene methylation relative to formaldehyde formation. This selectivity leads to higher turn-over numbers before deactivation by coking for DTH versus MTH. The reaction rate advantage of DTH versus MTH is more pronounced for medium-to-large (10-/12-ring) pore zeolites/zeotypes, since in small-window (8-ring) zeolites/zeotypes (CHA, AEI, ...) DME conversion is slower than methanol conversion due to slower diffusion of DME into the micropores.

### 3.2 Methanol to Dimethyl Ether (MTD)

Solid acids catalyze the dehydration of methanol to dimethyl ether (MTD), and the two most common catalysts are γ-Al_2_O_3_ and ZSM-5.^29,180^ The advantages of using γ-Al_2_O_3_ include low cost, high DME selectivity, and high catalytic and mechanical stability. The weak–moderate acidic sites of γ-Al_2_O_3_ catalyze methanol dehydration but are incapable of catalyzing the MTH reaction. Weakly adsorbed surface methoxy species form DME, while the strongly adsorbed surface methoxy species convert to surface formates, which subsequently decompose to CO, H_2_, and CH_4_.^181^ On the other hand, the strong acidic sites of ZSM-5 catalyze both reactions at higher reaction temperatures. ZSM-5 may be preferred over γ-Al_2_O_3_ for its superior hydrothermal stability and higher activity at low temperatures, but it has a lower reaction temperature window of 160–250 °C.^182^

As a connection between γ-Al_2_O_3_ and zeolites, Si-modification of Al_2_O_3_ was found to increase surface area and acidity, which led to improvement in catalyst lifetime at 300 °C, 1 bar, methanol/N_2_ = 0.11, and GHSV = 15,600 h^–1^.^183^ As another connection between γ-Al_2_O_3_ and zeolites, Olsbye and co-workers showed that porous aluminophosphates (i.e., AlPO-5 with AFI topology) could be used for MTD at higher temperatures of 450 °C because they contain weak acidic sites for MTD but not strong acidic sites for MTH.^99^

Improvements to the catalytic performance of zeolites/zeotypes focus on suppressing hydrocarbon selectivity and coking by eliminating the strong acidic sites, identifying suitable frameworks, and improving catalytic stability by coke studies. The first attempt to remove strong acidic sites in ZSM-5 using Na cations led to 100% DME selectivity over the temperature range of 230 to 340 °C.^184^ Various cations and zeolites with different Si/Al ratios were later screened for the liquid phase MTD reaction at 250 °C and 30 bar in a slurry reactor.^185^ Among the zeolites (ZSM-5, Y, MOR, FER, and BEA) with a similar Si/Al ≈ 20, MOR appeared to be the most active and selective. Subsequent ion-exchange of MOR with cations such as Zn, Ni, Al, Zr, Mg, and Na improved selectivity and stability through the removal of strong acidic sites. The catalytic performance of zeolites ZSM-5, MOR, BEA, and FER were also evaluated in typical fixed bed reactors from 180 to 300 °C, and the superior stability and low coke deposition rate of FER was attributed to its two-dimensional structure with smaller pores.^186,187^ The coke formation mechanism of ZSM-5 during MTD was studied at 200 °C using operando UV–Raman spectroscopy by Li et al.^188^ Methylbenzenium carbenium ions (MB^+^), a known coke precursor, was found to transform rapidly into “hard coke” at the top of the catalyst bed, but this transformation was suppressed toward the end of the catalyst bed due to water produced in the reaction.

**Figure 12:**
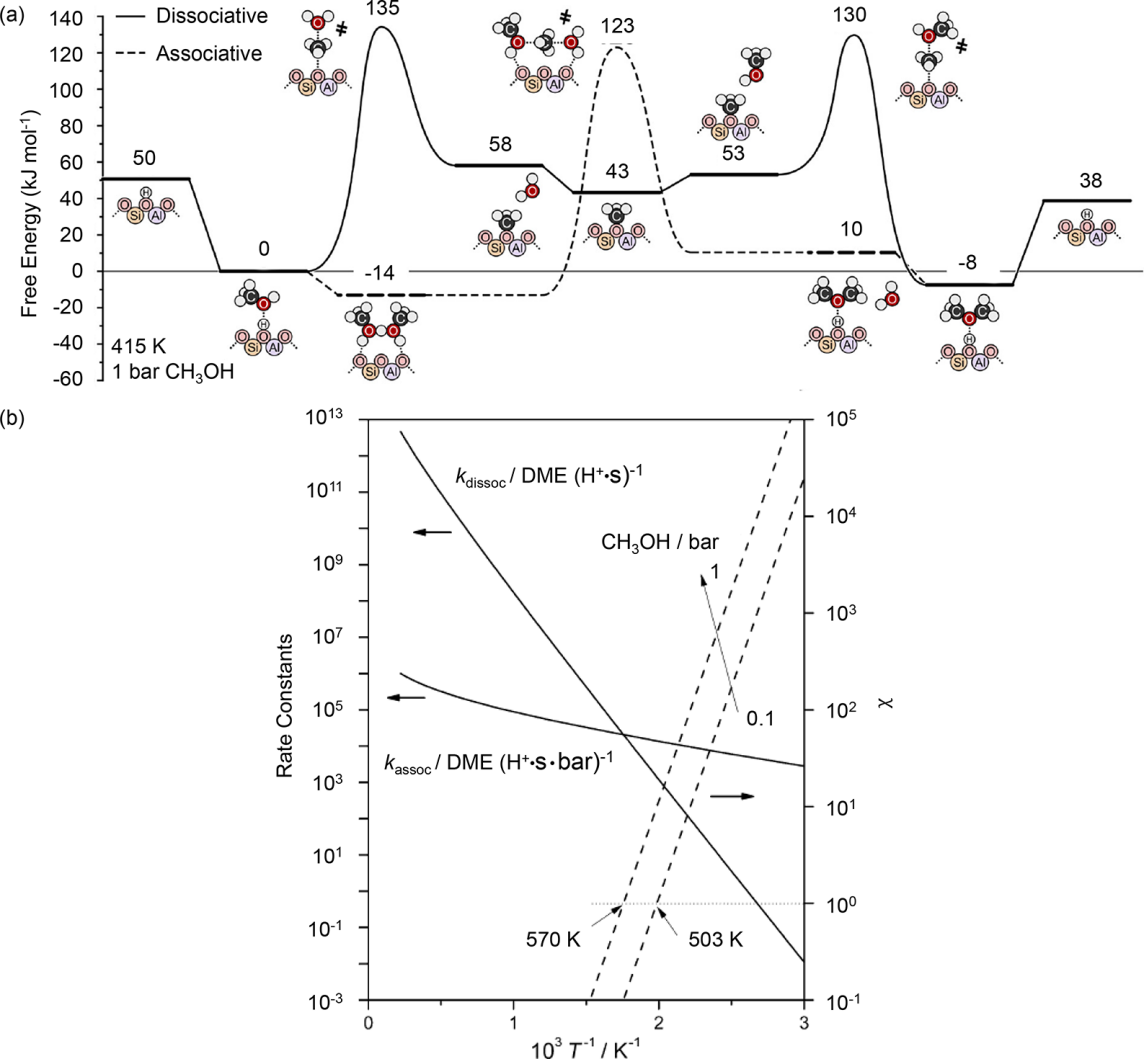
(a) Reaction coordinate diagram showing DFT-calculated free energies (ΔG; kJ mol^–1^) of intermediates and transition states involved in methanol dehydration on isolated H^+^ sites in CHA at 415 K and 1 bar of methanol. Reproduced with permission from ref (193). Copyright 2019 Elsevier. (b) Associative and dissociative rate constants as a function of temperature over ZSM-5. Reproduced with permission from ref (191). Copyright 2014 John Wiley & Sons.

In addition to catalyst development, mechanistic insight was needed to understand the origin of the influence of reaction parameters in MTD. There are two possible mechanisms, either concerted (associative) or sequential (dissociative) pathways, as shown in Figure 12a.^189−192^ In the direct associative mechanism, two methanol molecules coadsorb at the BAS to form a protonated methanol dimer. The protonated methanol dimer then rotates to form the transition state, which eliminates DME and water in a kinetically relevant step. In the sequential dissociative mechanism, a methanol molecule first adsorbs on a BAS and dehydrates to form a surface methoxy intermediate in a kinetically relevant step. The surface methoxy intermediate then reacts with another methanol molecule to form DME.

The preference of one mechanism over the other depends on reaction parameters, e.g., temperature, methanol partial pressure, and water partial pressure. Jones and Iglesia used a combination of kinetic, spectroscopic, and theoretical findings on ZSM-5 to support the associative mechanism being dominant at relevant temperatures and pressures for MTD, i.e., below 230 °C and 0.1 bar methanol or below 300 °C and 1 bar methanol (Figure 12b).^191^ At lower temperatures and higher pressures, the associative mechanism dominates due to the lower enthalpy transition state. At higher temperatures relevant for the title reaction, it is suggested that methanol–DME interconversions become equilibrated, and the dissociative mechanism dominates. These correlations were supported by a more elaborated van der Waals (vdW)-corrected DFT study on ZSM-5 with heterogeneous Al distribution.^192^ However, Grabow et al. showed that the absolute reaction temperatures and pressures in which the dominating mechanism crossovers from associative to dissociative depend on the location of the BAS, i.e., Al sitting, in ZSM-5. On the other hand, the DFT study on ZSM-22 (TON topology) by Moses and Nørskov concluded that the dissociative mechanism is dominating regardless of reaction temperature.^194^ Although water lowers the activation energies of key reactions in the associative mechanism, the dissociative mechanism remains more favorable.

The preference of one mechanism over the other also depends on the pore and cavity sizes of the zeolite, since confinement of the transition states would result in stabilization and a lower activation barrier. In this context, BAS descriptors such as acid strength, location, and proximity are also considered. It has been shown that the stronger the acid strength in ZSM-5, the more stable the transition states regardless of the reaction pathways.^192,194^ Jones and Iglesia surmised that the associative mechanism dominates in catalysts with larger voids, which could fit the larger dimeric transition state of the associative pathway, and such solvation by confinement would be beneficial. However, if the pore and cavity sizes were too small to accommodate an associative transition state, then the dissociative mechanism dominates. They subsequently verified their hypothesis over FAU, SFH, BEA, MOR, MTW, MFI, and MTT zeolites, and illustrated the negative relation between largest free sphere diameter and methanol dehydration rates.^195^ The confinement effect was exemplified as well in the MTD mechanistic studies using small-pore zeolites, i.e., CHA, AEI, LTA, and LEV, by the group of Gounder.^193,196^ Interestingly, the confinement effect was not always favorable in these small-pore zeolites, because the turnover rates of MTD became inhibited at high methanol pressures, i.e., > 0.1 bar at 142 °C.^193^ The origin of such a kinetic inhibition effect was proposed to be the stabilization of methanol clusters greater than three methanol molecules. This was not observed for the medium- or large-pore zeolites.

### 3.3 Integration of the MTD and DTH Reactions with CO_x_ Hydrogenation

DME is proposed to be a more efficient intermediate than methanol for the conversion of CO_x_ to hydrocarbons. DME has a lower H/C ratio than methanol, meaning that a broader ratio of syngas feed could be applied with a better C utilization. For instance, a net consumption of CO_2_ is achievable when the direct DME process starts with dry methane reforming.^197^ The direct DME process has more favorable thermodynamics, resulting in overall higher energy efficiency and higher conversion per pass.^198−200^ In addition, DME conversion limits formaldehyde formation and results in higher methylation rates in comparison to methanol, substantially prolonging catalyst lifetime.^99^ In view of the clear benefits on using DME as an intermediate, a critical assessment of the current status of such processes is needed before considering new strategies in this direction.

Generally there are three types of bifunctional catalysts that are able to convert CO_x_ to DME, by integrating methanol synthesis and methanol dehydration catalytic functions. The most obvious is the mixing of the two catalysts, the second type is the synthesis of core–shell catalysts, and the third is the use of oxide catalysts with acidic properties. The first two categories of catalysts have been reviewed, but the latter has scarcely been discussed.^29,180,201^ We will discuss relevant studies that focus on the MTD (zeolite) catalytic function in the tandem processes for CO_x_ conversion, before moving into oxide catalyst developments for CO_2_ hydrogenation to methanol and DME. We will also highlight improvements from process design, including reactor design and addition of adsorbents. Finally we present our case on the opportunities and risks on using DME as an intermediate, and our proposed solutions to mitigate the risks.

**Figure 13:**
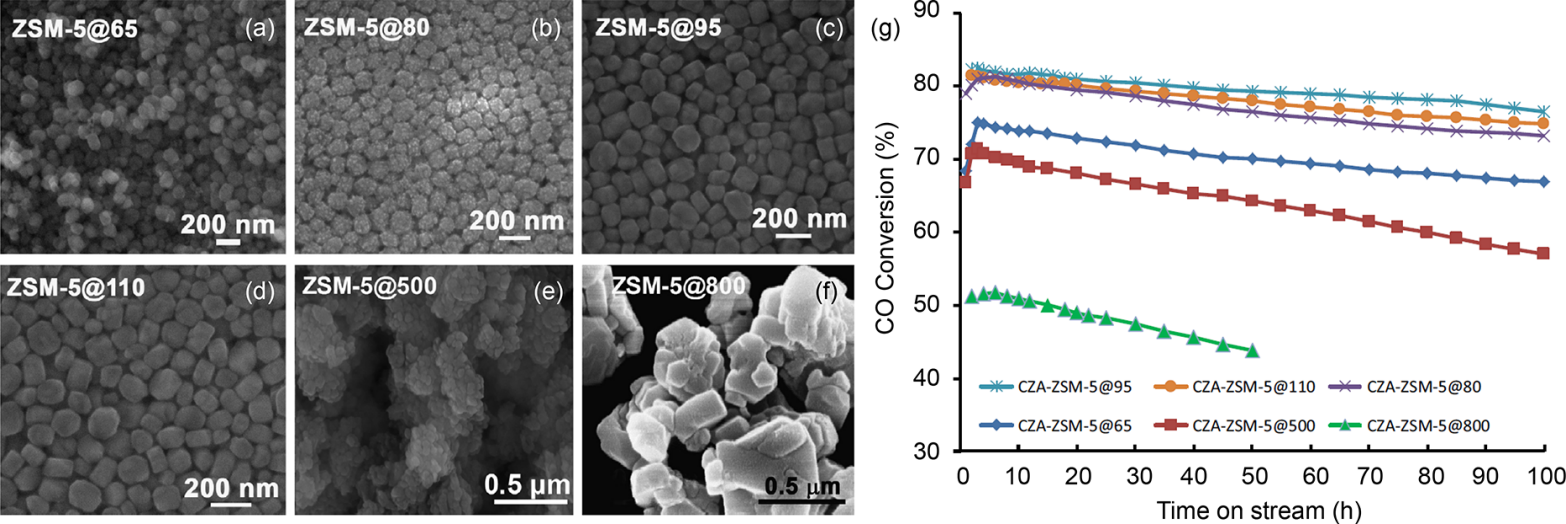
(a–f) Representative SEM images of ZSM-5 with varied crystallite sizes and (g) the effect of ZSM-5 crystal sizes on activity and stability for DME formation. Reaction conditions: 260 °C, 20 bar, H_2_/CO = 2, and 3600 mL/g_cat_/h. Reproduced with permission from ref (204). Copyright 2016 Elsevier.

For DME production from syngas, the industrial catalyst consisting of Cu, Zn, and Al (CZA) was almost universally used for the methanol synthesis catalytic function.^29,180^ ZSM-5 was typically used as reference, and studies include the influences of zeolite framework, ZSM-5 modification and particle size, proximity, and kinetics. The proximity effect was studied with different degree of mixing (slurry, grinding, pelletized) at 260 °C, 40 bar, and 1700 mL/g_cat_/h, and the catalysts prepared by slurry and grinding methods performed worse due to ion exchange of Cu^2+^ and Zn^2+^ to BAS.^202^ The physical mix of CZA and the ZSM-5 catalyst showed 89% CO conversion with 64% DME selectivity. Acidity of ZSM-5 was modified by MgO addition and evaluated at 260 °C, 40 bar, and 1500 mL/g_cat_/h (H_2_/CO/CO_2_ = 66/30/4). Dispersed MgO (∼5 wt %) introduced basic sites and LAS to ZSM-5 while decreasing BAS, leading to a decrease in CO_2_ and hydrocarbon selectivities to 31% and 0.4%, respectively (with 64% DME) at 95% CO conversion.^203^ Referring to Figure 13, crystallite size of ZSM-5 was varied between 65 and 800 nm, and activity (at 260 °C, 20 bar, and 3600 mL/g_cat_/h) was found to have an inverse relation with particle size due to mass transfer.^204^ The influence of the crystallite size was suggested to be stronger than the influence of BAS density. Catalyst deactivation was attributed to sintering of Cu nanoparticles on the BAS located at the external surface of ZSM-5. Reaction parameters and kinetics were also considered from 210 to 270 °C and from 10 to 50 bar for the CZA/ZSM-5 catalysts.^205^ Higher temperature and pressure were beneficial, while water cofeeding decreased methanol synthesis rates. The influence of zeolite topology was studied with ZSM-5, FER, IM-5, TNU-9, MCM-22, and ITQ-2 (Si/Al = 9–14) at 260 °C, 40 bar, and 1700 mL/g_cat_/h.^206^ Catalyst deactivation due to Cu nanoparticle sintering was found to be more detrimental than framework-induced coking and was further showed to correlate with BAS located at the external surface of zeolites. Thus, the close intimacy of the two catalytic functions was suggested to be unfavorable.

DME production from CO_2_ hydrogenation is more challenging due to unfavorable thermodynamics, which limits CO_2_ conversion to less than 30% at 200 to 280 °C and 30 bar. Kinetic studies showed that the formate formation step was rate-determining for methanol synthesis, resulting in a much slower methanol formation rate than methanol conversion rate.^207,208^ CZA was commonly used for methanol synthesis but other metallic catalysts such as Pd were explored recently due to the strong sintering of Cu nanoparticles in the presence of high steam partial pressure.^201,209^ The high steam partial pressure was detrimental for γ-Al_2_O_3_ as discussed earlier, resulting in superior performance of zeolites over γ-Al_2_O_3_ at CO_2_ hydrogenation conditions of 220 to 280 °C, 30 to 40 bar, and H_2_/CO_2_ = 3 to 4.^210−212^ Bonura et al. studied the influences of the mixing of CZA and ZSM-5, and of ZSM-5 acidity by varying Si/Al ratio from 15 to 100 at 240 °C, 30 bar, 10,000 mL/g_cat_/h, and H_2_/CO_2_ = 3.^213^ The zeolites with varied Si/Al ratios were tested only for MTD reaction, and Si/Al = 25 was selected based on activity and stability during water cofeeding. At 240 °C, 30 bar, 2500 mL/g_cat_/h, and H_2_/CO_2_ = 3, the physical mixture of CZA and ZSM-5 showed 38% DME, 10% methanol, and 52% CO selectivity at 15% CO_2_ conversion. The catalyst with CZA precipitated on ZSM-5 showed a slight 2% improvement on activity and selectivity. Bonura et al. extended their earlier study with FER zeolite of varied acidity and grain size.^214^ The catalyst CZA with nanosized FER (200–500 nm) was least active and selective toward DME in comparison to those with FER crystal size of > 1000 nm. The increase in BAS density appeared to cause larger Cu sintering and a faster deactivation rate, which was brought about by higher steam partial pressure.

To attain precise control of the proximity and intimacy of the two catalytic functions for STD, core–shell catalysts were pioneered by the group of Tsubaki in 2010.^215^ It was challenging to synthesize the zeolite shell over the CZA core because NaOH (commonly used in zeolite synthesis) could not be used to prevent damage to the CZA catalyst and also because the mechanical and hydrothermal stabilities of the CZA catalysts were poor. The core–shell catalyst consisting of a CZA core of 850 to 1700 μm and a zeolite shell of 4 to 5 μm attained the highest DME selectivity of 97% at 6% CO conversion (250 °C, 50 bar, and H_2_/CO/CO_2_ = 6/3/0.5). In comparison, the catalyst prepared by mixing attained 41% DME selectivity with 57% CO conversion. The same group extended the core–shell strategy to other zeotypes including SAPO-11 and SAPO-46, which simplified the synthesis protocol toward physical coating in view of potential scale-up and industrial relevance.^216,217^ Ateka et al. compared the core–shell (100–300 μm) and physically mixed catalysts consisting of CuO-ZnO-ZrO_2_ and SAPO-11 for syngas and CO_2_ conversion at 275 °C, 30 bar, and CO_2_/CO = 1.^218^ The core–shell and physically mixed catalysts attained 81% DME selectivity at 11% CO_x_ conversion and 77% DME selectivity at 9% CO_x_ conversion, respectively. Interestingly, the core–shell catalyst showed higher CO_2_ conversion than the physically mixed catalyst over 24 h TOS (4% vs 2%), and this was attributed to easier H_2_O diffusion from the methanol synthesis catalytic sites, which shifted the equilibrium. Modeling catalytic performance as a function of ZSM-5 wt % in the core–shell catalyst, Klumpp et al. highlighted the limitations of the core–shell catalysts in mass transfer and varying mass ratios of core and shell catalysts.^219^ Most literature agrees that the core–shell catalysts hold the advantage in DME selectivity but not activity due to mass transfer limitations.

In addition to the bifunctional catalysts, which consist of 2 distinct catalysts for methanol synthesis and conversion to DME, oxides with acidic sites are an emerging class of catalysts that are capable of producing DME from CO_x_. The industrial methanol synthesis catalyst CZA contains a small fraction of Al_2_O_3_, which could in principle catalyze methanol dehydration, but it is typically added as a stabilizer and binder for Cu and Zn, so its catalytic activity has not been considered previously. The Copéret group prepared 3 nm Cu nanoparticles (4 wt % Cu) supported on Al_2_O_3_ or SiO_2_ using the surface organometallic chemistry approach and evaluated their catalytic performance for CO_2_ conversion to methanol at 230 °C, 25 bar, H_2_/CO_2_ = 3, and < 7% CO_2_ conversion.^220^ The Cu/Al_2_O_3_ catalysts with LAS showed 30%, 15%, and 55% selectivity toward DME, methanol, and CO, respectively. On the other hand, Cu/SiO_2_ possessed no LAS, so only methanol and CO were formed. The ability of LAS on oxidic supports to catalyze methanol dehydration was also discussed by Prieto et al. in their investigation on LAS on oxidic supports as kinetic descriptors for CO_2_ conversion to methanol using Cu nanoparticles.^221^ Negligible DME was produced in their main study due to the low reaction temperature and conversion, which inhibited secondary reactions; however, the capability of LAS of the Ta, Al, Zr, Sc, and Y oxides to dehydrate methanol was verified in control experiments. A recent development from the Copéret group on SiO_2_-supported PdGa nanoparticles showing DME selectivity further demonstrates the potential of using a single catalyst with two active sites in closest intimacy to convert CO_x_ to DME.^222^ These catalysts could facilitate the rapid conversion of methanol to DME, after which DME would be the major intermediate to hydrocarbons.

**Figure 14:**
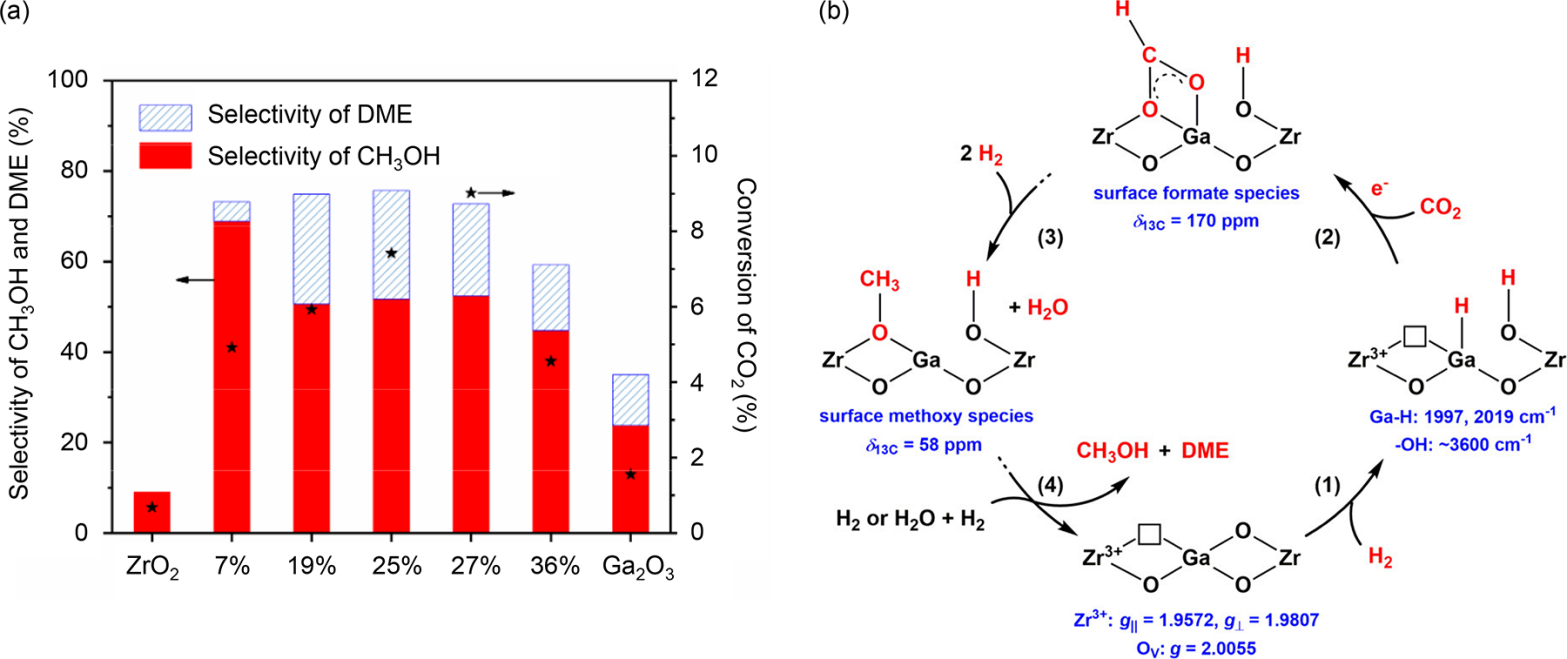
(a) CO_2_ conversion and selectivity toward methanol and DME over GaZrO_x_ catalysts and (b) the proposed mechanism. Reaction conditions: 330 °C, 30 bar, H_2_/CO_2_ = 3, and 24,000 mL/g_cat_/h. Reproduced from ref (225).

Moving from supported catalysts, several studies on mixed oxides mixed with zeolites for conversion of CO_x_ to hydrocarbons identified both methanol and DME as reaction intermediates.^154,155,223,224^ Notably, the Ye Wang group developed several state-of-the-art catalytic systems such as ZnZrO_x_ and ZnAlO_x_ coupled with various zeolites, and the observation of both methanol and DME in the product spectrum provided indirect evidence that the process proceeded via methanol and DME. They further demonstrated the capability of binary oxides with spinel structure to convert both CO and CO_2_ to an oxygenate mixture of methanol and DME at 400 °C, 30 bar, and H_2_/CO = 2 or H_2_/CO_2_ = 3.^155^ These spinel oxides were prepared by coprecipitation, and the best-performing catalyst ZnAlO_x_ showed the highest DME selectivity of 50% at 3% CO conversion, with the remaining product spectrum making up of 9% methanol, 6% hydrocarbons, and 35% CO_2_. Wu et al. reported another binary oxide GaZrO_x_ prepared by evaporation-induced self-assembly (EISA) to be selective toward DME and methanol from CO_2_ conversion.^225^ As presented in Figure 14a, ZrO_2_ showed 10% methanol selectivity but no DME (at < 1% CO_2_ conversion), while Ga_2_O_3_ showed ∼25% methanol and ∼10% DME selectivity (at ∼1.5% CO_2_ conversion) at 330 °C, 30 bar, 24,000 mL/g_cat_/h, and H_2_/CO_2_ = 3. GaZrO_x_ with 19 to 27 wt % Ga loading showed around 50% methanol and 25% DME selectivity at 6 to 9% CO_2_ conversion. Detailed characterization performed to understand the synergistic effects of the bimetallic components resulted in the proposed reaction mechanism shown in Figure 14b. Importantly, DME was suggested to be formed directly from hydrolysis and hydrogenation of surface methoxy species, instead of sequentially via methanol dehydration.

Considering the latest developments in CO_x_ conversion and CO_2_ utilization, there are several research questions and opportunities in the direction of DME-mediated conversion to hydrocarbons. First, new classes of catalysts including supported catalysts and bulk oxides are capable of converting CO_x_ to DME on a single catalyst instead of the conventional bifunctional catalysts consisting of copper-based methanol synthesis catalysts and zeolites for methanol dehydration. For the supported catalysts, LAS seems to be a key descriptor for DME production and could be introduced by support doping.^222^ For the bulk oxides, key descriptors for DME formation are less obvious because there are more variables such as synthesis method, elements and their loading/mixing, structure, and size. For instance, GaZrO_x_ prepared by coprecipitation and tested at 300 °C and 20 bar was not reported to produce DME, while the previous case study showed GaZrO_x_ to produce ∼0.5 mol of DME per mol of methanol.^225,226^ Thus, catalyst development and identification of key descriptors for the conversion of CO_x_ to DME would be useful for further improvements to the DME-mediated tandem process. Second, mechanistic studies thus far focused on active sites for CO_2_ and H_2_ activation, and much less is known about the active sites for methanol or DME formation. In the proposed reaction mechanisms, both methanol and DME originated from surface methoxy species, but it is rather ambiguous on when DME would be formed rather than methanol. One mechanism hypothesized that methanol was formed from the surface methoxy species and should dehydrate to form DME.^155^ The other mechanism inferred that DME could be formed directly from hydrolysis and hydrogenation on surface methoxy species.^225^ Distinguishing such mechanistic details would be critical for designing active sites for DME formation.

High steam partial pressure remains to be a challenge in the tandem process, and this may be tackled from either catalyst design or process and reactor design. From the catalyst design perspective, the introduction of hydrophobicity into catalysts to suppress water gas shift kinetics has been increasingly explored. This strategy has been proven to be effective for syngas conversion via Fischer–Tropsch Synthesis (FTS),^227−230^ and it has potential in syngas conversion via oxygenate intermediates as discussed in this Review. Using Cu/ZnO and ZSM-5 as a reference bifunctional catalyst for syngas conversion to DME, the functionalization of the Cu/ZnO nanoparticles using stearic acid to increase hydrophobicity appeared to be effective and resulted in higher DME selectivity.^231^

**Figure 15:**
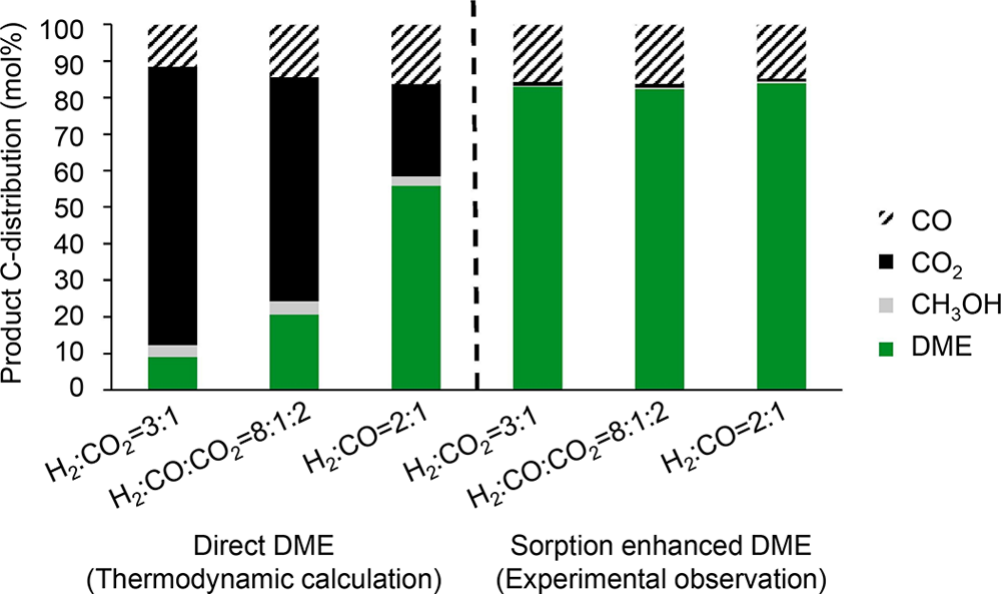
Thermodynamic (maximally possible) carbon distribution versus experimentally obtained results for sorption-enhanced DME synthesis. A H_2_O break-through time of approximately 20 min was indicated for the experimental data reported in this figure. Reaction conditions: 275 °C, 40 bar, and stoichiometric H_2_ to CO_x_ feed as shown in figure. Reproduced with permission from ref (233). Copyright 2019 Elsevier.

From a process intensification perspective, steam partial pressure could be limited by the addition of adsorbents or the use of membrane reactors, and in both cases, zeolites play an essential role as the adsorbent and membrane materials.^232,233^ Van Kampen et al. authored a recent review on steam separation enhanced reaction processes, in which they discuss the technical aspects and outlook of these technologies.^233^ Of relevance is the sorption-enhanced DME synthesis from CO_x_ conversion, in which the benefits of adding adsorbents to the direct DME synthesis process is demonstrated (Figure 15).^27,234^ At 275 °C, 25 bar, and H_2_/CO_2_ = 3, the addition of an LTA zeolite adsorbent to a typical bifunctional catalyst of CZA and γ-Al_2_O resulted in > 80% CO_2_ conversion and > 70% DME selectivity per pass. Notably, sorbent-enhanced processes require frequent sorbent regeneration by pressure or temperature swing, thereby complicating process design and operation. Optional reactor configurations are moving bed and fluidized bed reactors.^27^ The LTA zeolite is also commonly selected as a membrane for in situ water removal in the relevant processes such as methanol synthesis and MTO.^235,236^ ZSM-5, MOR, and SIL were theoretically evaluated as membranes for the production of DME from CO_2_ but were found to be unsuitable.^237^ These zeolites had low permselectivity toward water, resulting in high loss of methanol. Zeolite membrane reactors are also simulated for CO_2_ conversion to hydrocarbons via FTS, and it was concluded that the in situ removal of water enhanced hydrocarbon yield by shifting the RWGS equilibrium to a certain extent, and a further increase in water removal led to changes in hydrocarbon product distribution and hot spot formation.^238^ As concluded by van Kampen et al., these technological advances in process intensification are mostly attained from theoretical thermodynamic calculations, and the feasibility and potential should still be addressed experimentally.

Last but not least, the tandem process concept of conversion of CO_x_ to DME or hydrocarbons is reassessed. Bansode and Urakawa^239^ reported experimental results for CO_2_/H_2_ conversion over Cu/ZnO/Al_2_O_3_ alone or mixed with ZSM-5, at an exceptionally high pressure of 360 bar. The CZA catalyst reached > 95% CO_2_ conversion and > 98% methanol selectivity at 260 °C with a CO_2_/H_2_ ratio of 1:10, almost identical to the equilibrium yield under these conditions. A high H_2_ content in the feed was required to enhance the rate of methanol formation. A mixture of CZA with H-ZSM-5 at the same pressure and CO_2_/H_2_ ratio yielded 97% CO_2_ conversion and 89% DME selectivity at 300 °C. Hydrocarbon production was targeted by a two-stage approach, in which CZA was loaded into the first and H-ZSM-5 into the second of two reactors in series, where the first was operated at 360 bar, 260 °C, and CO_2_/H_2_ = 1:10, and the effluent was led into the second reactor which was operated at 375 °C and either 360 or 1 bar. Operation at 360 bar yielded 95% CO_2_ conversion with 4% selectivity to DME and 85% selectivity to C_1_–C_4_ alkanes, while operation at 1 bar yielded 97% CO_2_ conversion with 10% selectivity to DME, 23% to C_1_–C_4_ alkanes, and 42% to C_2_–C_3_ alkenes.

**Figure 16:**
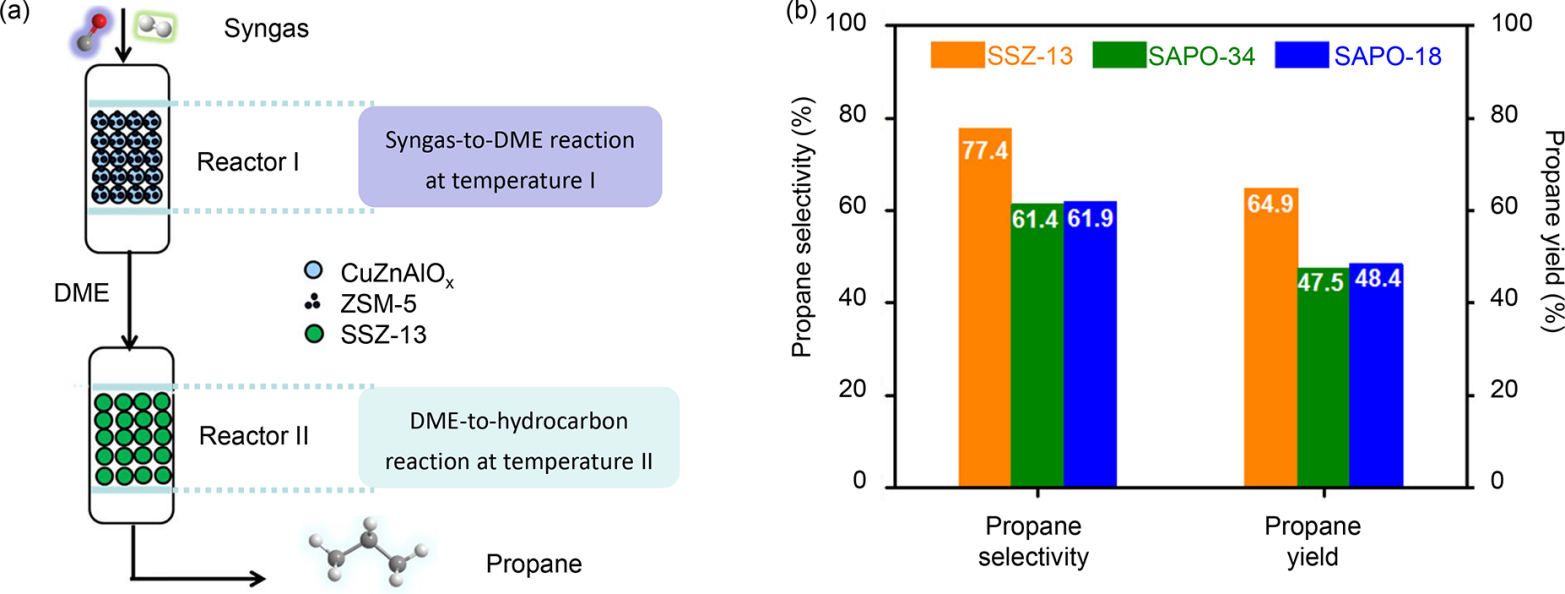
(a) Configuration of the dual-bed catalyst system for syngas-to-propane and (b) effect of different zeolites in the lower bed on the selectivity and yield of propane. Reaction conditions: T (upper bed) = 250 °C, T (lower bed) = 410 °C, 50 bar, H_2_/CO = 7, and 4000 mL/g_cat_/h. Reproduced from ref (241).

The two-stage reactors-in-series concept is also proposed by the team of Liu for the conversion of syngas to gasoline or propane, and the advantage of decoupling reaction conditions, i.e., lower temperatures for DME synthesis and higher temperatures for DME conversion to hydrocarbons, is exemplified.^240,241^ The same group had earlier demonstrated a dual-bed reactor concept for DME synthesis and conversion to produce olefins from synthesis gas.^242^ To produce gasoline, the first reactor was loaded with CZA and Al_2_O_3_ catalysts to convert syngas to DME at 260 °C, and the second reactor was loaded with a nanosized ZSM-5 (Si/Al = 97) zeolite to convert DME to C_5_–C_11_ hydrocarbons at 320 °C.^240^ At 30 bar and H_2_/CO = 2, 79% C_5_–C_11_ hydrocarbon selectivity (excluding 32% CO_2_ selectivity) was achieved at 87% CO conversion. Larger ZSM-5 crystals and a lower Si/Al ratio resulted in lower activity and faster catalyst deactivation. To produce propane as illustrated in Figure 16a, CZA and ZSM-5 catalysts were loaded in the first reactor operating at 260 °C, and an SSZ-13 zeolite was loaded in the second reactor operating at 410 °C.^241^ At 50 bar and H_2_/CO = 7, propane selectivity reached 77% (excluding 11% CO_2_ selectivity) at 96% CO conversion. SAPO-34 and SAPO-18 zeotypes were also studied, and the lower acid strength resulted in a poor catalyst lifetime and lower propane selectivity (Figure 16b). Furthermore, the Si/Al ratios in SSZ-13 were varied, and there appeared to be a positive relation between the BAS density and propane selectivity. Three observations could be made regarding the two-stage reactors-in-series concept. First, this concept is more attractive for the production of propane than gasoline/aromatics from CO/CO_2_/H_2_, since a higher reaction temperature is needed, which means a larger temperature gap between methanol synthesis and conversion. Second, a higher BAS density is preferred for the selective production of propane but not for gasoline, and this points to different requirements of the zeolite component in such processes. Third, under the conditions of this study, the propane productivity increased from 5 mmol/g/h for the single-reactor test to 7 and 14 mmol/g/h, respectively, for the two reactors in series without and with a methanol dehydration catalyst (ZSM-5) in the first reactor.

## 4 Ketene-Mediated CO_x_ Conversion to Hydrocarbons

### 4.1 Methanol Carbonylation

Methanol carbonylation to acetic acid is an industrial process of more than 15 million tonnes per annum.^243^ The industrial catalysts are homogeneous Rh or Ir complexes, and the reaction conditions are typically 200 °C and 30 bar. Halogen promoters/cocatalysts are required to facilitate the process. This process was first commercialized in 1960 by BASF using a Co iodide catalyst at 250 °C and 700 bar, and the BASF process was subsequently phased out due to the Monsanto process, which operated at milder conditions.^244^ The Monsanto process was developed in the late 1960s and is based on homogeneous Rh complex catalysts operated at 150 to 200 °C and 30 to 60 bar. In addition to the challenges associated with homogeneous catalysis, e.g., catalyst costs, stability, and recovery, a drawback of the Rh-based catalysts is the requisite of high water partial pressure to prevent precipitation of the homogeneous catalyst. The high water partial pressure and the WGS activity of the Rh sites result in 10% undesired CO_2_ selectivity; hence, a breakthrough was made by BP in 1996 with the development of the Cativa process.^245,246^ The Cativa process utilizes homogeneous Ir-based catalysts and has a similar process window as the Monsanto process, but it operates at low water partial pressure. The low partial pressure coupled with low WGS activity of the Ir-based catalysts imply that > 99% acetic acid selectivity is achieved.

To eliminate the above-mentioned challenges of processes catalyzed by homogeneous catalysts, heterogeneous catalysts based on immobilizing the homogeneous complexes on supports were developed.^243,247^ Most of such heterogeneous catalysts still require iodide cocatalysts, which would increase cost and complexity due to its high corrosivity. Detailed studies of the homogeneous Rh system suggested that iodide, beyond its role as cocatalyst, enhances the nucleophilicity of the metal complex in the transition state, which leads to methyl group ligand insertion.^248^ Zeolites are considered as support materials for the homogeneous complexes due to favorable dispersion,^249^ but discussion on this application of zeolites is beyond the scope here.

Inspired by the acid-catalyzed “Koch” reaction, Fujimoto and colleagues demonstrated in 1984 the feasibility of using zeolites to catalyze methanol carbonylation.^250^ In this study, zeolite-Y (FAU topology), MOR, and ZSM-5 were tested at 200 to 300 °C, 9.8 bar, and CO/methanol = 1. Although DME was the major product (∼90% selectivity) in all cases, acetic acid and methyl acetate were observed in the product spectrum. Cu incorporation to MOR via ion exchange was found to increase the carbonylation rate but did not change the product distribution. Smith from BP and his collaborators later optimized the Cu-MOR catalyst and process conditions (350 °C, 10 bar, CO/methanol = 10, and GHSV = 3000 h^–1^) to attain > 70% selectivity of acetic acid and methyl acetate.^251^ However, selectivity stability remained a challenge as this selectivity was stable for 12 h TOS, and hydrocarbons and DME were the major products during the first 5 h and last 5 h of reaction, respectively.

The Corma group, in collaboration with BP, utilized spectroscopic tools (operando FTIR and in situ magic-angle spinning NMR) to determine reaction intermediates during methanol carbonylation over MOR and Cu-MOR catalysts.^252^ Cu-MOR was selected as a bifunctional catalyst to contain a function for stabilization of methyl species and a function for CO activation, thereby overcoming the rate-determining CO insertion step. The same intermediate methoxy and acylium cations were observed over both MOR and Cu-MOR catalysts; however, acrylic acid and methyl acetate were the corresponding final products. In the case of MOR, H_2_O adsorption dominated, resulting in acrylic acid. On the other hand, Cu-MOR possessed an active site composed of two neighboring sites, one bridged hydroxy and a neighboring Cu^+^. The BAS was responsible for methanol activation, while Cu^+^ accounted for CO activation and preferential DME adsorption over methanol and H_2_O, resulting in methyl acetate production.

A relevant tandem catalysis concept was developed by the Hargreaves lab together with BP to convert methanol to acetic acid without CO feed.^253^ A Pd/CeO_2_ catalyst was used to decompose methanol to generate CO in situ, and the subsequent methanol carbonylation was catalyzed by Cu-MOR. At 300 °C, 1 bar, methanol/Ar = 0.4, and GHSV = 10,400 h^–1^, a mixture of CO, DME, and acetic acid was produced in approximate ratios of 300:40:1 in a stacked bed configuration. The stacked bed configuration performed better than the physically mixed bed, because methanol could decompose to formaldehyde and ultimately the needed CO. In a physically mixed bed, formaldehyde would be in close proximity with a BAS and transformed to coke before splitting to CO. This connects with the strategy of Hwang and Bhan to suppress deactivation in the MTO process, when they added Y_2_O_3_ to CHA catalysts to decompose formaldehyde into CO and H_2_ before formaldehyde transformation to polyaromatics (vide ultra, Section 2.1).

The detrimental influence of H_2_O on methanol carbonylation, stemming from parallel methanol dehydration and/or competitive adsorption, is the main driving force for the shift toward DME carbonylation. A recent breakthrough was achieved by the Liu group with their pyridine-modified MOR catalysts operating at 250 °C, 50 bar, and CO/methanol = 400.^254^ The higher reaction temperature was used to counter the H_2_O effects, and the pyridine modification served to block the 12-MR channels to inhibit hydrocarbon selectivity and coke formation. Hence, the pyridine-modified catalyst showed > 90% acetic acid selectivity at full conversion over 145 h TOS.

Beyond zeolites, Qi et al recently reported 54% acetic acid selectivity when feeding methanol and CO over Na-promoted Rh nanoclusters on ZrO_2_ support at 573 K, with 1.4% CO conversion.^255^ Even more recently, the same group achieved 96% selectivity to acetic acid over the Rh-ReO_4_/SiO_2_ catalyst at 543 K, with 39% CO conversion at a CO:methanol feed ratio of 1:1 (30 mbar each). In both cases, DME was the main byproduct.^256^ Comparative tests of a series of catalysts revealed that DME is formed on bulk Re_2_O_7_ and ReO_x_ crystals, while acetic acid is formed on atomically dispersed ReO_4_.

### 4.2 DME Carbonylation

The Iglesia group in cooperation with BP in 2006 introduced the DME carbonylation process with > 99% methyl acetate selectivity over zeolite catalysts at 150 to 190 °C.^257^ The selectivity decreased at higher temperatures due to hydrocarbon formation. The rate of methyl acetate formation decreased from MOR to FER to ZSM-5 and was not detectable for USY and BEA at 147 to 240 °C, 10 bar, and CO/DME/Ar = 93/2/5. The rate of methyl acetate synthesis was independent of DME partial pressure but increased with CO partial pressure, hence, suggesting reactions of gas-phase or adsorbed CO with DME-derived intermediates to be rate-determining. H_2_O cofeeding with DME and CO experiments verified that DME carbonylation was more favorable due to the lack of competitive adsorption between H_2_O and CO at LAS and/or parallel methanol dehydration reactions. The elementary steps of DME carbonylation were proposed to be reversible dissociative adsorption of DME to form surface methyl species, followed by CO insertion to form surface acetyl species, and finally reaction of surface methyl and acetyl species to desorb methyl acetate.^258^ There were two distinct sites: one site stabilizes acidic protons and methyl/acetyl species, while the other site is responsible for binding CO. In order to locate the selective sites for DME carbonylation, Na^+^ or Co^2+^ ion-exchange was performed on MOR to replace selectively H^+^ sites in 8-MR and 12-MR channels, respectively.^259^

**Figure 17:**
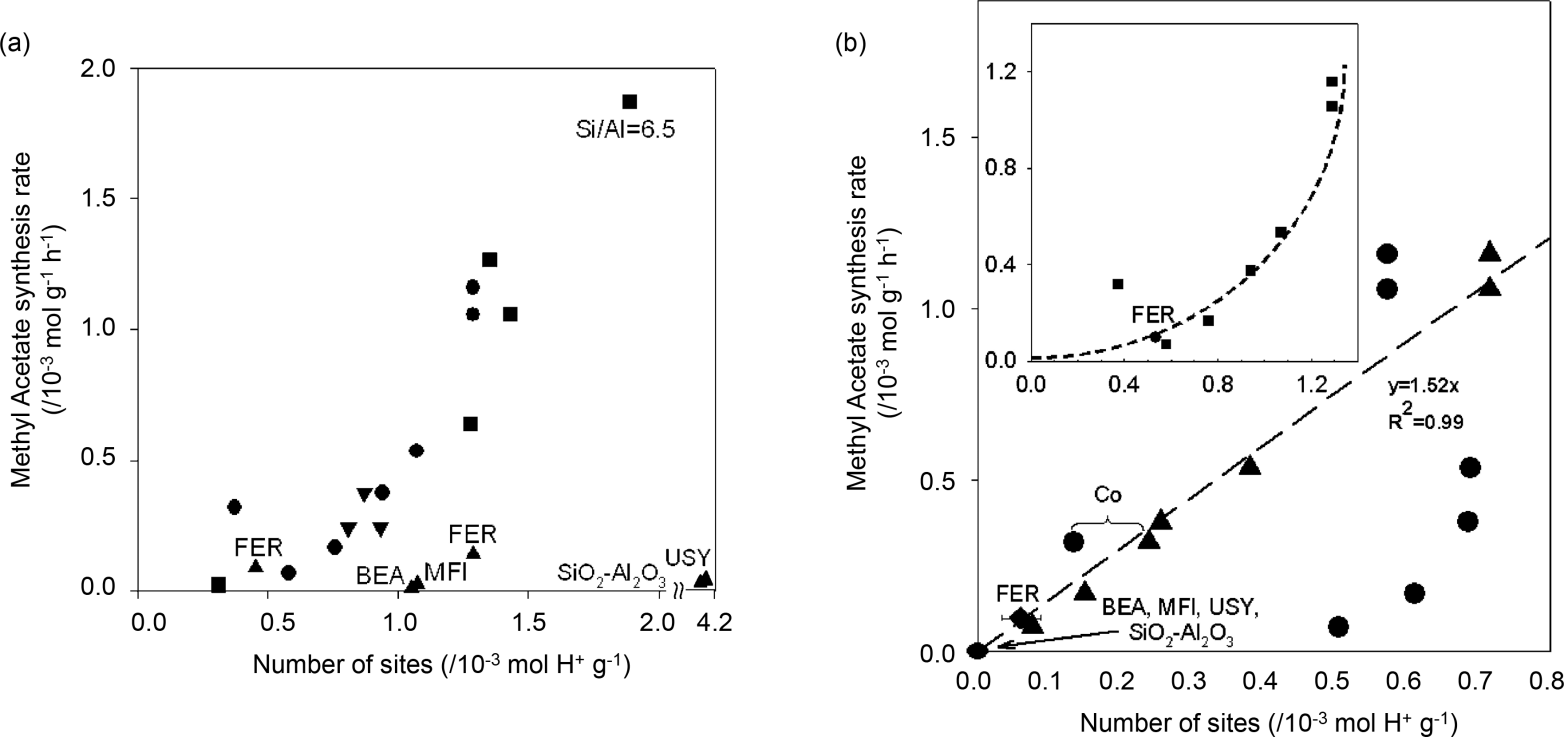
DME carbonylation rates plotted against (a) the number of total H^+^ sites in MOR (●,■,▼) and other zeolites (▲) and (b) the number of H^+^ sites per unit mass in 8-MR channels of MOR (▲), FER (◆), and 12-MR channels of MOR (●). Inset shows DME carbonylation rates plotted against the total number of H^+^ sites in these samples. Reaction conditions: 165 °C, 10 bar, and CO/DME/Ar = 93/2/5. Reproduced from ref (259).

As demonstrated in Figure 17, the formation of C–C bonds via CO insertion was concluded to occur selectively in 8-MR channels, which matched well with the reactivity in zeolites with 8-MR channels (MOR and FER) and the lack of reactivity in zeolites without 8-MR channels (BEA, FAU, and MFI). Several theories were put forward to explain the 8-MR site requirements, including selective stabilization of cationic transition states, confinement and solvation effects, and lower ion-pair enthalpies together with lower entropies.^260−262^

**Figure 18:**
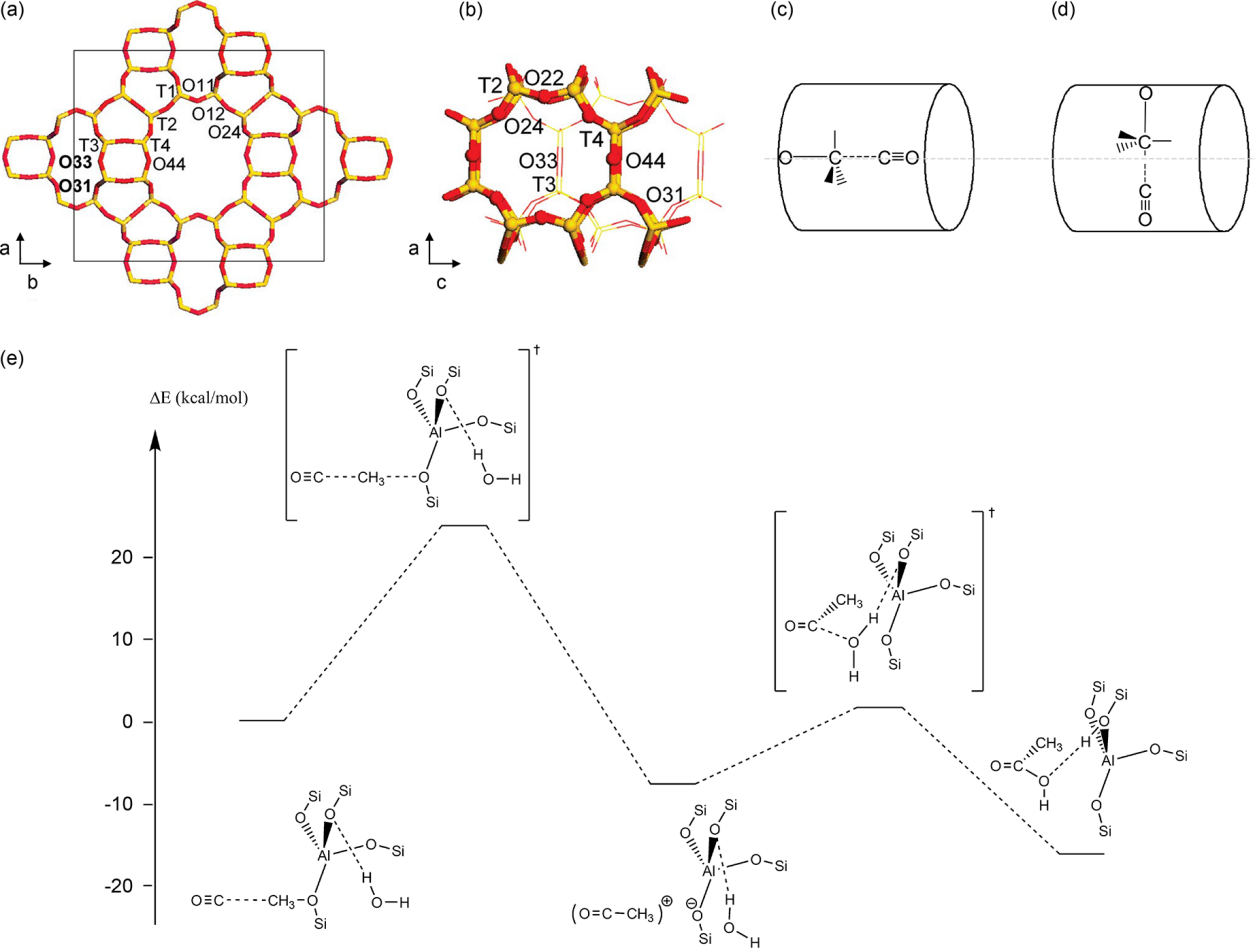
Structure of MOR in the (a) *c* and (b) *b* directions, with the respective O labeling. Schematic representation of the relative orientation of the O framework-CH_3_ bond and the channel axis at (c) the T3-O33 position of MOR and (d) any other position in an 8-MR channel. (e) Calculated energy profile for methanol carbonylation at the T3-O33 position. Reproduced from ref (263).

The above studies on MOR were continued by Corma and co-workers with quantum-chemical methods so as to locate the active sites for methanol and DME carbonylation.^263^ From Figure 18, four nonequivalent tetrahedral sites in the MOR unit cell were considered, namely T1 in the 12-MR channel, T2 and T4 in the intersection between 12-MR and 8-MR, and T3 in the 8-MR channels. At the T1, T2, and T4 positions, the formation of DME and hydrocarbons were kinetically favored over carbonylation due to lack of steric hindrance. The T3-O33 position was the only selective site for carbonylation because at this position, the methoxy group was parallel to the cylinder axis, which allowed the attack of CO to fit perfectly without steric hindrance, as presented in Figure 18. From the calculated energy profile at the T3-O33 position (Figure 18), the transition state for the attack of the CO on the methoxy group possesses the highest activation energy of 23.9 kcal/mol. The acylium cation reaction intermediate is 6.9 kcal/mol more stable than the initial state, and 9.8 kcal/mol less stable than the final state. This theoretical analysis validated prior experimental studies, which proposed CO insertion to the surface methoxy group as the rate-determining step. During methanol carbonylation, H_2_O or methanol reaction with the acylium cation would produce acetic acid, and the resulting methoxy/water ratio = 1. During DME carbonylation, methyl acetate would be formed instead, and the resulting methoxy/water ratio = 2. Thus, the negative influence of water was doubled during methanol carbonylation, and the formation of water clusters could block the 8-MR channels.^264^ The negative effect of H_2_O on carbonylation rates originated from competitive adsorption of CO and H_2_O, and the displacement of equilibrium toward reactants that decreased the abundance of surface methoxy groups for CO insertion. To disintegrate the water clusters and to compensate for the lower rates, methanol carbonylation required higher reaction temperatures than DME carbonylation, which in turn decreased selectivity toward acetic acid.

**Figure 19:**
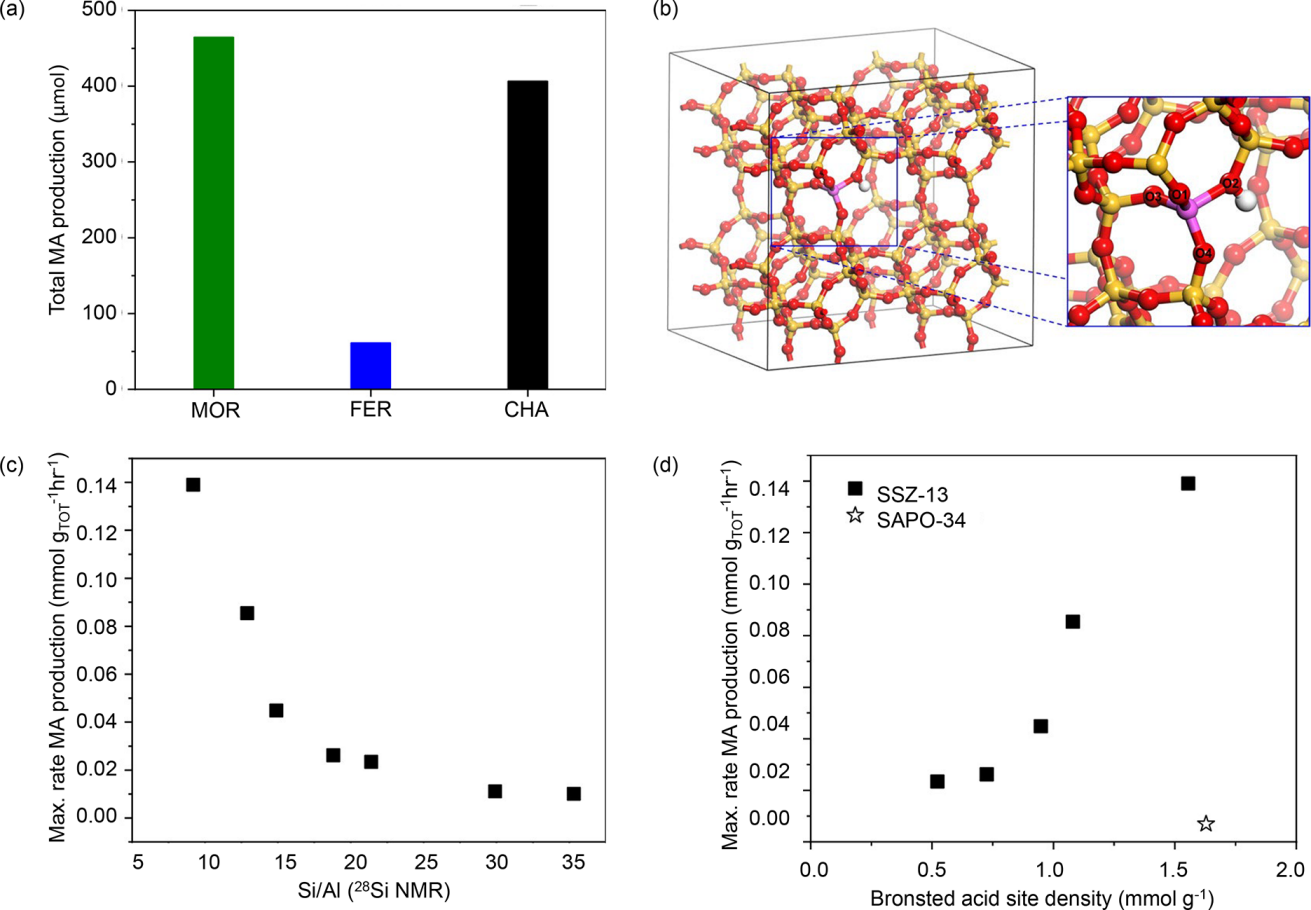
(a) Total methyl acetate (MA) production after 24 h of TOS over each framework. (b) 2 × 2 × 2 supercell of zeolite CHA with Al substituted at the sole T site to form a Brønsted acid site (BAS). Close-up viewpoint of the Brønsted acid site depicts the four crystallographically distinct O sites. Atom colors: Al (purple), Si (yellow), O (red), and H (white). (c) Maximum rates of MA production measured over the SSZ-13s as a function of Si/Al. (d) Maximum rate of MA production as a function of BAS density for SSZ-13s and SAPO-34. Reaction conditions: 165 °C, 1 bar, and CO/DME/He = 95/2/3. Reproduced from ref (289).

Several studies devoted to the in situ characterization of the MOR zeolites supported the hypothesis of acetyl intermediates. In situ solid-state NMR studies on three different MOR catalysts (MOR with both 8-MR and 12-MR channels and MOR with accessible 8-MR or 12-MR channels) provided direct evidence of methoxy species and, notably, acetyl species in 8-MR channels.^265^ In situ DRIFT spectroscopy revealed that the acetyl species were formed only at higher CO partial pressures of 5 to 30 bar.^266^ In situ solid-state NMR was also used to clarify the nature of the acetyl intermediate, and a covalent acetyl–zeolite complex was identified rather than the usually assumed acylium cation.^267^

Ketene was first proposed based on DFT calculations by Jensen et al. to be a potential reaction intermediate in MOR-catalyzed DME carbonylation.^268^ The low energy barrier (8–11 kJ/mol) for the conversion of acetyl carbocation to ketene was in a range similar to that for the direct conversion of acetyl carbocation to acetyl (1–20 kJ/mol), making both pathways feasible. To prove this hypothesis, D_2_O was introduced in the feed upon reaching a steady state at 165 °C, 10 bar, and CO/DME = 98/2. As the reaction between D_2_O and ketene was the only possibility to form doubly deuterated acetic acid, the observation of such a molecule confirmed the presence of ketene.

Much attention has been placed on the 8-MR channels due to its reactivity, but the role of the 12-MR channels should not be neglected. Both experimental and theoretical studies point to the 12-MR channels functioning as the main mass transfer routes in MOR.^269,270^ Due to the size of the 12-MR channels, which accommodates aromatic molecules, coking in the 12-MR channels leading to catalyst deactivation is common. Thus to improve on the catalytic TON of MOR zeolites, synthesis strategies including nanosizing of the crystals,^271,272^ selective dealumination,^273,274^ and blocking of 12-MR channels were employed.^275^

Promoter addition is often used in catalysis to improve performance, and Cu modification to MOR was already attempted in the first publications. Recently, Ma and co-workers performed a study in which the nature and amount of Cu species in H-MOR were systematically varied by applying various insertion and pretreatment methods. The materials were characterized by XRD, TEM, and CO adsorption IR studies and XPS. Then, they were subjected to testing as DME carbonylation catalysts at 15 atm and 200 °C in a flow reactor. A positive correlation was found between the amount of Cu^0^ in nanoclusters and the carbonylation reaction rate, up to a certain amount of Cu, where Cu migrated to the outer surface of the zeolite and grew into larger particles. On the other hand, the correlation between Cu^+^ amount and DME carbonylation rate was negative, possibly because Cu^+^ was covering BAS in the 8-ring pockets of MOR. Further experimental and computational results suggested a synergistic role of Cu^0^ and the BAS, in which DME adsorbs on the BAS, forming methanol and a methyl group, which adsorbs on Cu^0^ and reacts with coadsorbed CO and the methanol to finally form methyl acetate.^276,277^ To maintain Cu stability and dispersion, Zn could be added together with Cu via ion exchange.^278^ In the screening of Cu, Ni, Co, Zn, and Ag, the cations exchanged in the 8-MR channels, i.e., Cu, Ni, and Co, improved the catalysts.^279^ In a study focused on Co-MOR, Co^2+^ ions were exchanged in the 8-MR channels at low loading (Co/Al < 0.1 and Si/Al = 8.5) but were also present in the 12-MR at higher loading Co/Al = 0.1–0.25).^280^ Co^2+^ ions at the 8-MR channels facilitated the adsorption of both CO and DME molecules, while those at the 12-MR channels reduced BAS density, leading to less coking. Besides ion exchange, which decreases BAS density, framework heteroatom substitution is an option to introduce promoters. For example, Fe heteroatoms were incorporated in the 12-MR channels of the MOR framework, resulting in lower acidic strength and density which ultimately reduced carbon deposition.^281^ Ce heteroatom substitution into the framework also resulted in lower acidic strength, but the positive influence on catalytic performance was proposed to be due to an enrichment of Al sites in the 8-MR channels.^282^

MOR is the clear favorite for DME carbonylation, and in the far second spot is FER, which consists of 8-MR and 10-MR channels. The 10-MR channels of ZSM-35 (FER) are close to the size of a benzene molecule, hinting that diffusion of aromatic molecules and corresponding hard coke formation could be inhibited, arguably increasing the competitiveness of FER from the catalytic stability angle.^283^ Although the surface methoxy groups were calculated to form preferentially in both 8-MR and 10-MR channels of FER, CO attack on the surface methoxy group took place selectively at the 6-MR zone of the 8-MR channel of FER zeolites.^284^ Accordingly, the focus has been on positioning BAS sites in the 8-MR channels, and synthesis strategies include the use of various structure-directing agents to direct Al,^285^ and the seeding and recrystallization approach.^286^

Other zeolites containing 8-MR channels, including the ETL, SZR, and CHA topologies, are recently explored. The EU-12 (ETL topology) zeolite is two-dimensional with two types of straight 8-MR channels intersected by one type of sinusoidal 8-MR channel.^287^ At 220 °C, 15 bar, CO/DME/Ar = 93/4/3, and 2400 mL/g_cat_/h, the highest selectivity toward methyl acetate of 90% was attained at ∼16% DME conversion. Coke was formed mainly on the external surface of ETL due to its small channels, instead of within the channels as in MOR and FER. The SUZ-4 (SZR topology) zeolite is three-dimensional with a 10-MR channel intersected by two arrays of 8-MR channels.^288^ At 200 °C, 20 bar, CO/DME/Ar/He = 50/5/2.5/42.5, and 1250 mL/g_cat_/h, 90% methyl acetate selectivity and 10% DME conversion were stable for 100 h of TOS. The abundant 8-MR pore openings on the rod-shaped SUZ-4 facilitated the diffusion of the reactive molecules, leading to superior catalytic stability. Interestingly, zeolite topologies MOR, FER, IRN, ATS, and GON were theoretically screened using Monte Carlo associated with molecular dynamics simulation, and ATS and IRN zeolites were highlighted as potential candidates based on both diffusion dynamics and reaction kinetics.^270^

Industrial zeolites with MFI and CHA topologies have also been considered. At 165 °C, 1 bar, CO/DME/inert = 95/2/3, 30,000 mL/g_cat_/h, and Si/Al = 10, SSZ-13 (CHA) was less active than MOR but more active than FER (Figure 19a).^289^ According to DFT calculations in Figure 19b, the favored sites were located within the plane of the 8MR window, in agreement with the MOR literature. Comparing SSZ-13 and SAPO-34 of similar BAS density (1.7 mmol/g), the weaker acidic strength of SAPO-34 led to lower coverage of methoxy and CO surface species, resulting in lower activity (Figure 19c and d). This positive relation between acidic strength and activity is also reported for ZSM-5 (MFI) in which acidic strength was varied by substituting framework Al with heteroatoms Ga and B.^290^

### 4.3 Integration of Methanol and DME Carbonylation with CO_x_ Hydrogenation

**Figure 20:**
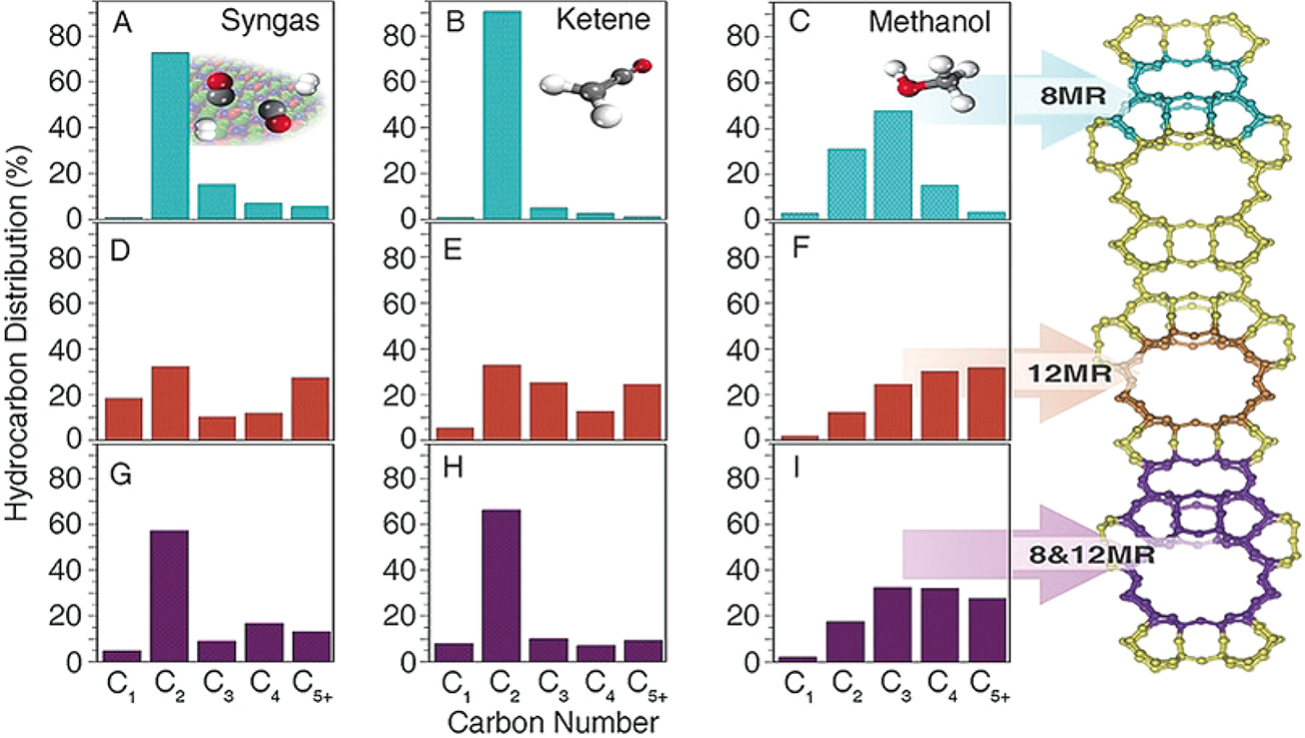
Hydrocarbon distribution in the conversion of syngas, ketene, and methanol over different sites of the MOR zeolites at 375 °C. (a–c) MOR#2-py with only the 8-MR acid sites accessible, (d–f) MOR#14 with only the 12-MR acid sites accessible, and (g–i) MOR#3 with both the 8-MR and 12-MR acid sites available. (a, d, and g) Syngas over ZnCrO_x_-MOR, (b, e, and h) ketene conversion over MOR, and (c, f, and i) methanol conversion over MOR. Reproduced with permission from ref (291). Copyright 2018 John Wiley & Sons.

In the first report on the Ox-Zeo process, Bao and co-workers proposed ketene to be the key intermediate in the conversion of syngas to lower olefins using ZnCrO_x_/SAPO-34 catalysts, because ketene was detected using vacuum ultraviolet photoionization mass spectrometry.^33^ The methanol cofeeding experiments leading to a drastic drop in catalytic performance substantiated the importance of ketene instead of methanol. In order to exploit the ketene intermediate for steering hydrocarbon selectivity, the solid acid component was switched from SAPO-34 to MOR zeolites.^291^ At 360 °C, 20 bar, H_2_/CO = 1, and 1857 mL/g_cat_/h, 73% ethylene selectivity (excluding 48% CO_2_ selectivity) was achieved at 26% CO conversion using a ZnCrO_x_/MOR catalyst combination in which the MOR had only 8-MR sites accessible to reactivity. Employing solid-state NMR spectroscopy and ^129^Xe as a probe molecule, the preferential adsorption of ketene in the 8-MR sites and methanol in the 12-MR sites were verified. The subsequent correlations of hydrocarbon selectivity to the number of sites accessible in the 8-MRs were consistent with the proposition of ketene as a key intermediate (Figure 20). To broaden the scope, ZnGa_2_O_4_, Ga_2_O_3_, and ZnO were mixed with MOR and tested at 400 °C, 40 bar, H_2_/CO = 2.5, and 1600 mL/g_cat_/h.^292^ Arguing that since the three oxides produced similar levels of methanol and DME and an identical MOR was used for the formation of hydrocarbons, the differences in hydrocarbon selectivity pointed to the presence of ketene as an intermediate. Interestingly, the ex situ NMR analysis detected carbonylation products including acetate and propionate. Specifically, these carbonylation products and the initial C–C bond via ketene were proposed to be formed on the oxide surface.

With the intention to determine if the key intermediate is indeed ketene or methanol, theoretical understanding of the ZnCrO_x_ systems is needed.^293,294^ DFT calculations and microkinetic simulations on the highly reduced ZnCr_2_O_4_ (110) surface revealed the propensity of CO to absorb on the O vacancy sites, followed by reaction with H to form a CHO surface species.^293^ The distinction between ketene and methanol is dependent on what happens next to the CHO surface species. To form ketene, CHO is first dissociated to CH and O, followed by hydrogenation to CH_2_ and finally CO insertion. To form methanol, CHO goes through consecutive hydrogenation steps. Quantitatively, the pathway to ketene requires less energy, so ketene is more readily formed than methanol. This finding is supported by a parallel microkinetic modeling study on the ZnCr_2_O_4_ (111) surface, which is calculated to be the most stable surface under reaction conditions.^294^ Accordingly, the surface coverage by CH_3_CO (ketene precursor) is 11 times higher than that by CH_3_O (methanol precursor).

**Figure 21:**
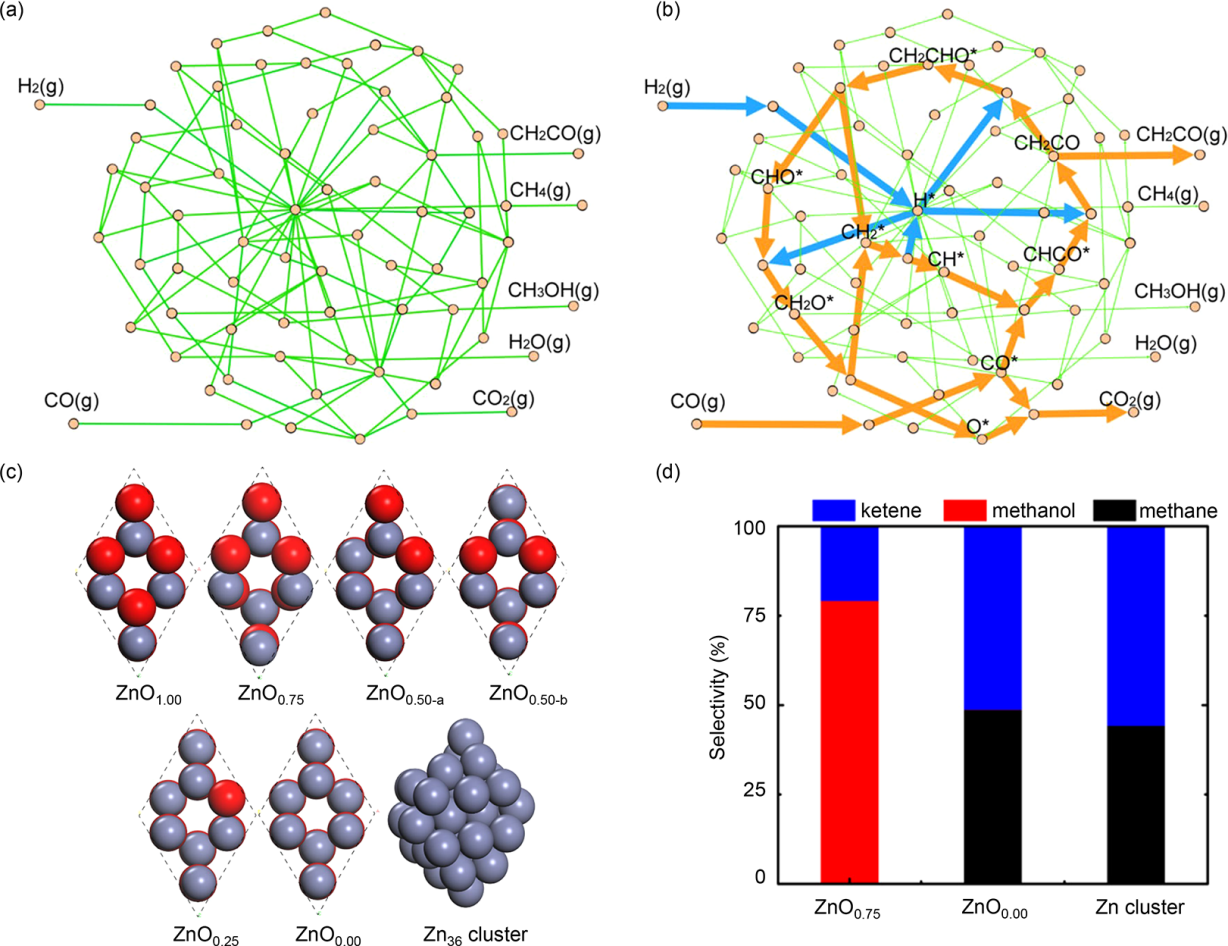
(a) Complete reaction networks of syngas to methane, ketene, and methanol and (b) a specific reaction pathway for ketene production. Orange arrows represent the evolution cycle of carbon-containing species, and blue arrows represent hydrogenation and dehydrogenation. (c) Collection of ZnO surfaces with a varying concentration of oxygen vacancies. A Zn_36_ cluster is used to account for the reactivity of small Zn clusters observed in experiments. (d) Microkinetic modeling results of selectivity over ZnO_0.75_, ZnO_0.00_, and the Zn cluster. Reproduced from ref (295).

Although the above theoretical studies supported the hypothesis of ketene as a key intermediate, they were applicable only for ZnCrO_x_ catalysts, and such calculations should be extended to other relevant bulk oxides. With reference to Figure 21, a comparable study on ZnO surfaces found a correlation between surface oxygen vacancies and product selectivity.^295^ Although ketene is formed on all ZnO surfaces, the methanol production is favored over the more oxidized ZnO surfaces. On a ZnO_0.75_ surface, the selectivity toward methanol and ketene is 80% and 20%, respectively. With an increase in the number of oxygen vacancies, the major product evolves from methanol to ketene to methane. On a metallic Zn_36_ cluster surface, the methane and ketene selectivities are 57% and 43%, respectively.

**Figure 22:**
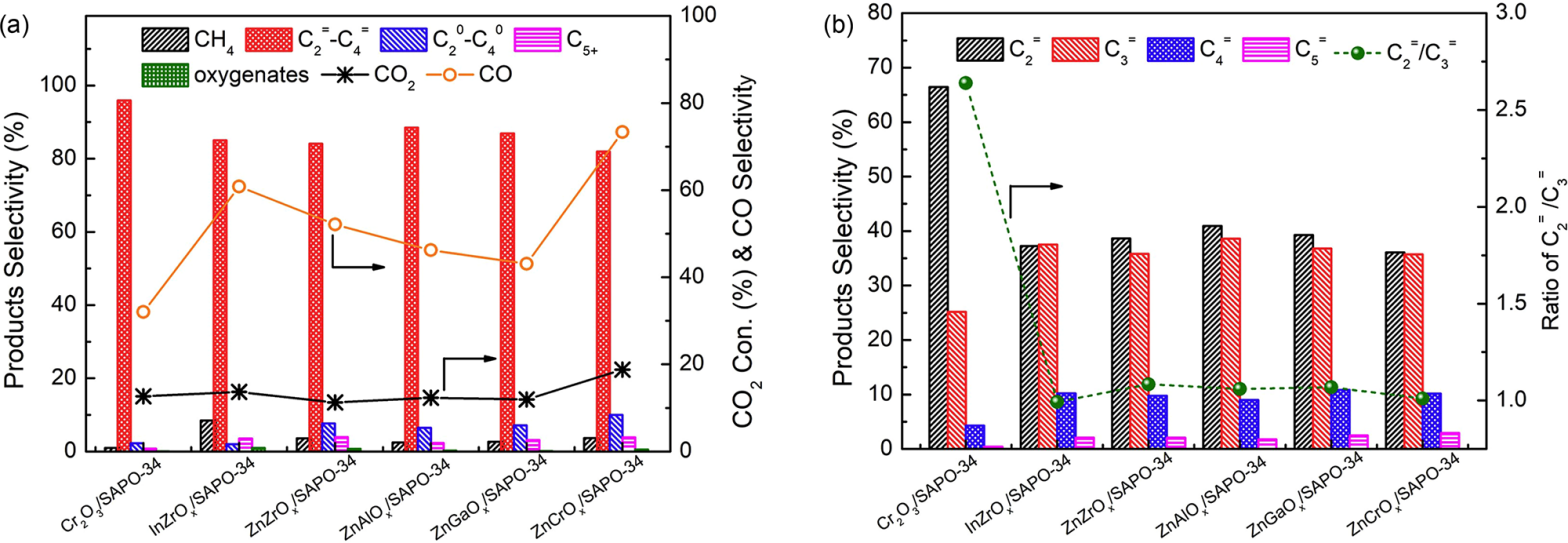
(a) CO_2_ conversion and product distribution and (b) olefins selectivity over various metal oxide/H-SAPO-34 composite catalysts. Reaction conditions: 370 °C, 5 bar, H_2_/CO_2_ = 3, and 4000 mL/g_cat_/h. Reproduced with permission from ref (296). Copyright 2022 Elsevier.

The scope of the ketene-mediated process is expanded with a recent contribution from the Fan group which compared the performance of a set of methanol synthesis catalysts, Cr_2_O_3_, InZrO_x_, and ZnMO_x_; M = Zr, Al, Ga, and Cr, with SAPO-34, under a given set of conditions (Figure 22).^296^ In this case, InZrO_x_ and ZnMO_x_ mixed with SAPO-34 yielded very similar product distributions, both with respect to CO versus hydrocarbon selectivity and C_2_:C_3_:C_4_ ratios. Notably, the product distribution obtained with the Cr_2_O_3_/SAPO-34 mixture was distinctively different, with the hydrocarbon selectivity to C_2_ being 66%, compared to 37% over both InZrO_x_/SAPO-34 and ZnMO_x_/SAPO-34. Mechanistic investigations showed that acetic acid (as an intermediate product) and ethanol (as a final product) were present both on the surface and in the effluent from Cr_2_O_3_, together with methanol. Mixing Cr_2_O_3_ with SAPO-34, ethanol was rapidly converted to ethene, thereby yielding the superior ethene selectivity of this tandem catalyst. The reason why ethene persisted in contact with SAPO-34, instead of being incorporated into the hydrocarbon pool, may be linked to the diffusion restrictions of ethene in small-pore zeolites such as SAPO-34, which were recently explored by the van Speybroeck group.^297,298^ Comparatively, only methanol was detected as the surface and effluent product from ZnZrO_x_. When mixed with SAPO-34, this catalyst yielded a product distribution more similar to that obtained over SAPO-34 under MTO conditions.^139^

**Figure 23:**
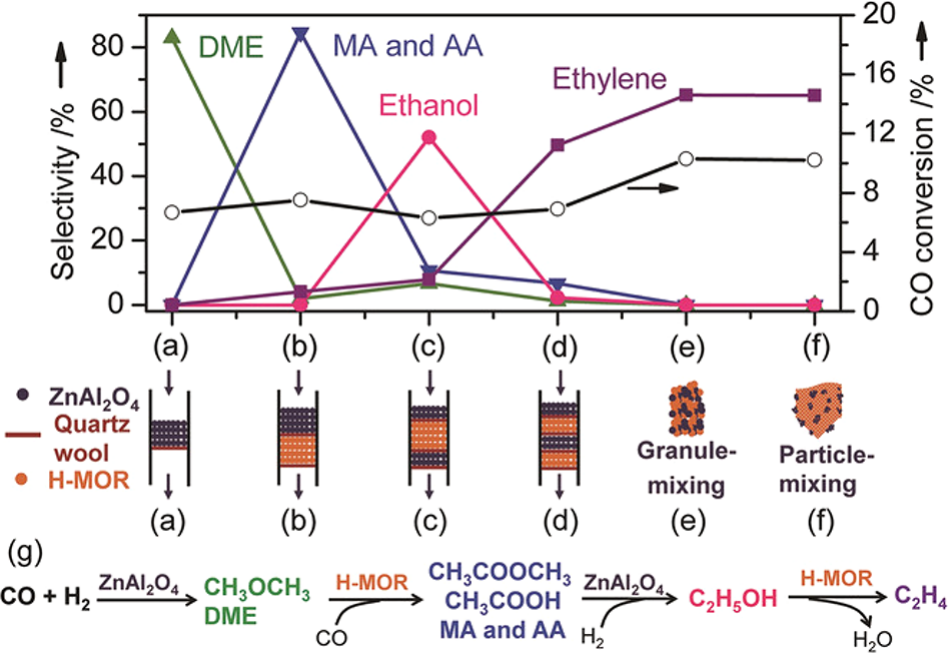
Effect of arrangements of ZnAl_2_O_4_ and H-MOR on catalytic conversion of syngas. (a) ZnAl_2_O_4_. (b) ZnAl_2_O_4_; H-MOR. (c) ZnAl_2_O_4_; H-MOR; ZnAl_2_O_4_. (d) ZnAl_2_O_4_; H-MOR; ZnAl_2_O_4_; H-MOR. (e) Mixture of ZnAl_2_O_4_ and H-MOR granules with sizes of 250–600 mm. (f) Mixture of ZnAl_2_O_4_ particles with sizes of 4–9 nm and H-MOR particles with sizes of 0.3–1 mm. Reaction conditions: H_2_/CO = 1; P = 3 MPa; F = 25 mL min^–1^; time on stream = 20 h; total weights of ZnAl_2_O_4_ and H-MOR = 0.33 and 0.67 g. Reproduced with permission from ref (299). Copyright 2018 John Wiley & Sons.

Besides the above studies which highlight ketene as an important intermediate and ethene as a main product, the more classic approach to integrate DME carbonylation with syngas conversion results in acetic acid, methyl acetate, and ethanol as major products. The proof-of-concept was demonstrated by Wang and co-workers, in which syngas was first converted to DME using a mixed bed of CZA and HZM-5 catalysts, and the effluent stream containing DME was passed through MOR to produce methyl acetate and acetic acid.^299^ Due to the poor CZA catalyst stability at higher temperatures, a ZnAl_2_O_4_ catalyst was used to convert syngas to DME at higher temperatures between 325 and 370 °C. At 370 °C, 30 bar, and H_2_/CO = 1, 87% selectivity toward methyl acetate and acetic acid (excluding 20% CO_2_ selectivity) was attained at 11% CO conversion. This concept was extended to steer the product selectivity toward ethanol and ethylene by means of catalyst bed configurations (see Figure 23). Recognizing the value of ethanol, a follow-up study was devoted to improving ethanol selectivity via catalyst development, catalyst bed configuration, and reaction parameter optimization.^300^ The optimal catalyst configuration was three stacked beds of K/ZnO/ZrO_2_, MOR, and Pt/Sn/SiC catalysts for the formation of methanol, acetic acid, and ethanol, respectively. At 270 °C, 50 bar, and H_2_/CO = 1, 80% ethanol selectivity (excluding < 10% CO_2_ selectivity) was achieved at 4% CO conversion. Notably, Al in the 12-MRs of the MOR catalyst was selectively dealuminated to improve the acetic acid selectivity.

Focusing back to the progress in zeolite research, strategies were inspired from prior work on Koch carbonylation, such as improved CO binding, increasing the number of selective sites in the 8-MRs and decreasing the number of unselective sites in 12-MRs in the zeolites. For instance, Cu modification on MOR was investigated and expectedly promoted CO activation and carbonylation.^301^ However, it was also found that the enhanced electrostatic interaction between Cu^+^ and the acetyl cation resulted in ethane formation. As this latter observation differs from prior understanding, this could be due to the differences in reaction conditions (e.g., feed composition, temperature, and pressure). Another example is the selective phosphate passivation of the BAS in the 12-MR channels using trimethylphosphite as a phosphate precursor, resulting in over 96% selectivity toward C_2_ oxygenates.^302^

Last but not least, the core–shell capsule catalyst approach was investigated by means of a Cu/ZnO core and a micron-sized MOR zeolite shell.^303^ Importantly, the influence of MOR shell thickness between 2 and 40 μm was studied, and a minimum of 20 μm thickness was required to attain 50% ethanol selectivity at 220 °C, 15 bar, H_2_/CO = 1, and 10–14% CO conversion. In addition, Cu was introduced via ion exchange to enhance DME carbonylation in the MOR shell.

The successful development of carbonylation catalysts that are active at moderate temperatures hints to an alternative route for C_3+_ olefins formation, in which acetic acid may be reduced to form ethanol and potentially dehydrolyzed to ethene and subjected to a second carbonylation step. Along this line, Ahlers et al. recently reported the selective formation of 1-propanol from CO_2_, H_2_, and ethene over supported Au catalysts.^304,305^ The reaction was found to proceed by reduction of the CO_2_ to CO over the Au nanoparticles, followed by selective carbonylation and subsequent partial hydrogenation to form propanol. Small Au nanoparticles on a K-promoted TiO_2_ support were found to yield close to 100% selectivity to 1-propanol at 20 bar, 200 °C, and CO_2_/H_2_/C_2_H_4_=1:1:1.

Another moderate-temperature path to C_3+_ olefins from ethene is the oligomerization reactions. Industrially, ethene oligomerization processes are carried out over metal complex catalysts in solution using organometallic cocatalysts.^306^ Major efforts have been made in recent years to develop heterogeneous analogues for this process, including zeolite-based catalysts. Those processes are not yet competitive with the homogeneous processes, but some interesting observations have been made and are mentioned here. Please note that a detailed assessment of the oligomerization process is beyond the scope of this study, and we refer the interested reader to two recent, excellent review papers by Finiel et al. and Olivier-Bourbigou et al.^307,308^ Our brief description focuses on studies carried out in fixed bed reactors with ethene gas flow.

As a first observation, studies of catalysts consisting of Ni ions impregnated or ion-exchanged onto Si-Al-O supports, showed that they are active for ethene oligomerization without a cocatalyst, regardless of the (micro-, meso- or macro-) pore structure of the support material.^308^ Experimental and theoretical evidence led several groups to conclude that reaction between a sacrificial ethene molecule, an open Ni coordination site, and a neighboring Brønsted acid site leads to the formation of a Ni–H site, active for ethene oligomerization. However, the issue is still under debate.^308^ Focusing next on zeolite supports, the Martinez group studied Ni-exchanged and -impregnated H-Beta zeolite and reported a linear increase in ethene conversion with increasing Ni content, until the molar concentration of Ni corresponded to the Brønsted acid concentration of the parent sample, where conversion leveled out. They concluded that single Ni ion sites are responsible for ethene oligomerization.^309^ The conclusion has found support from several independent studies of Ni-zeolite catalysts.^308^ The Martinez group study was carried out at 120 °C, 26 bar ethene pressure, and WHSV (ethene) = 2.1 h^–1^. It further showed that the selectivity to odd-numbered alkenes decreased with increasing Ni content for Ni/H < 1, suggesting carbocation-mediated cracking over free BAS.^309^ Beyond the active site, several studies point to the influence of zeolite pore size and structure. The Hulea group reported a steep increase in ethene conversion level with an increase in pore size of MCM materials, at 150 °C, 35 bar, and 1 h reaction in batch mode.^307^ They ascribed the difference to rapid coke formation, leading to pore blocking in the microporous materials. Recent studies showed that the microporous structure of the zeolite alters both reaction kinetics and product selectivity compared with amorphous supports. Koch et al. studied the kinetics of ethene oligomerization over 1.8 wt % Ni/SiO_2_-Al_2_O_3_ at 443–503 K, ethene partial pressure 1.5–3.5 bar, and τ = 4.8–14.4 kg_cat_·s·mol_C2_^–1^. Microkinetic modeling of the test data revealed a first-order reaction in P(ethene) and a Schulz–Flory product distribution, in accordance with the degenerate polymerization (Cossee–Arlman) mechanism.^310^ The same product distribution was observed for Na-exchanged Ni/Y zeolite (11.2 Å cavity size) at 100–150 °C, 35 bar, and WHSV (ethene) = 2 h^–1^. Recently, experimental studies of Ni/SSZ-24 (7.3 Å pore size) at P(ethene) = 4–26 bar and 130–170 °C revealed a second-order reaction rate in ethene with 35 kJ/mol activation energy, and > 98% selectivity to linear butenes at 1–7% conversion. DFT-based static and molecular dynamics simulations pointed to a degenerate polymerization mechanism and suggested that the second-order rate dependence on P(ethene) is due to formation of an [(ethene)_2_-Ni-ethyl]^+^ ion which is detached from the framework position and stabilized by the framework in the transition state, leading to a substantially decreased activation energy for butene formation. The high C_4_ selectivity was allocated to lower rotational freedom of the corresponding [(ethene)_2_-Ni-butyl]^+^ ion, leading to retained, higher activation energy for C_6_ formation.^311,312^

Overall, lower activity and more rapid deactivation due to pore clogging by coke make Ni/zeolite-based ethene oligomerization catalysts less attractive than their mesoporous counterparts. Future efforts may potentially challenge this conclusion by using lamellar zeolite sheets that retain shape selectivity but offer shorter diffusion paths. Carrying out the process at temperatures and pressures where ethene is in a condensed state, may further promote the heterogeneously catalyzed process by lowering the product desorption energy, as recently demonstrated by Agirrezabal-Telleria and Iglesia for Ni/MCM-41.^313^ The product solvation effect of condensed ethene leads to a dramatic reduction in catalyst deactivation. However, a recent study revealed that this effect is only observed for mesoporous catalysts, while microporous catalysts (BEA and FAU) suffer from pore clogging by hydrocarbon residues even under capillary condensation conditions. The reason may be that the microporous pores are too small to enable packing of the ethene molecules around the active site containing the alkyl intermediate.^314^

## 5 Conclusions and Outlook

CO_2_ valorization is a key technology for the postfossil society, where it will enable production of consumer goods with properties equal to those obtained by converting fossil carbon, as well liquid fuels for energy storage. The oxygenate-mediated Ox-Zeo tandem process for syngas (CO + H_2_) conversion to hydrocarbons in a single reactor and its expansion to comprise CO_2_ hydrogenation in that same reactor have the potential to become a leading technology for such conversion. It offers exceptional selectivity to desired product ranges: Light olefins, BTX, or gasoline range aromatic/paraffin blends. Several decades of zeolite and zeotype catalyst studies of oxygenate (methanol and dimethyl ether) conversion into hydrocarbons yields a platform for further selectivity optimization.

Recent contributions cited in this Review demonstrate that the characteristic shape selectivity offered by zeolites and zeotypes dominates effluent product composition in the tandem process, to a similar extent as in methanol and dimethyl ether to hydrocarbons processes. However, they also demonstrate that the altered gas composition induced by the tandem process, especially the H_2_, CO, and H_2_O feed content relative to methanol, influences product formation rate and selectivity. The presence of the external, methanol-producing cocatalyst alters product formation rate and selectivity even further, especially in cases where the zeolite/zeotype pores are large enough to enable diffusion of intermediates and products in and out of the zeolite/zeotype crystals for intermittent interaction with the hydrogenation catalyst.

From a process perspective, a major advantage of the methanol-mediated conversion of CO_x_ and H_2_ to hydrocarbons is the superior TON of this process compared to MTH alone, especially for 8-ring window-cavity structures such as SAPO-34. This feature may enable the use of fixed bed reactors instead of the fluidized bed reactor with continuous regeneration that is currently used for the SAPO-34-catalyzed MTO/DMTO process. On the other hand, the rather low product formation rates induced by the thermodynamically limited, low methanol/water ratio in the tandem reactor may require recycle rates comparable to those used in the methanol-forming reactor of conventional reactor-in-series processes. Rapid conversion of methanol to dimethyl ether could probably mitigate this limitation to some extent due to the higher equilibrium conversion to DME than methanol at a given set of conditions. Recent contributions further suggest that a two-reactor tandem approach may attenuate the conversion limitation, by optimizing the conditions in the first reactor for high DME yields and those in the second reactor for the MTH/DTH conversion. The two-reactor approach maintains the advantage of the tandem process in terms of catalyst TON, due to the ensemble of gas components fed with methanol and DME to the second reactor. On the other hand, the opportunity of direct, synergetic interaction between the two catalyst functions is lost.

Another option to maximize product yield and conversion rates is continuous removal of water in the tandem reactor or between two-stage reactors. However, further studies are needed to elucidate the effect of water removal on coke production.

Focusing beyond effluent product composition and into the zeolite/zeotype, cofeed and tandem process studies combined with operando spectroscopy and (transient) Guisnet-type analysis of retained hydrocarbon and carbohydrate species clearly demonstrate that the hydrocarbon pool composition is strongly affected by the presence of H_2_, CO, and water. More precisely, hydrogen cofed with methanol leads to hydrogenation of precursors to aromatics within the zeolite, resulting in dramatically higher turn-over numbers (TON) before deactivation by coke formation than for a methanol-only feed. Higher acid strength promotes hydrogenation more than sites with lower acid strength. CO, when cofed with methanol, leads to enhanced carbonylation activity and is incorporated in aromatic products, while simultaneously limiting the conventional hydrogen transfer route to aromatics formation. When cofed with H_2_ and methanol, CO hinders olefin hydrogenation reactions while maintaining the high TON numbers installed by H_2_ cofeed. H_2_O cofed with H_2_ and methanol has a similar effect on olefins hydrogenation as the CO/H_2_/CH_3_OH cofeed but also leads to degradation of the zeolite framework over time. Very recently, the observation of abundant carbonylated species during steady-state operation of tandem catalyst systems by using quasi-in situ solid-state NMR and ^12^C/^13^C feedstock led to the proposal of a “triple cycle” mechanism. Here, carbonylation reactions via cyclopentenyl species is suggested as the main route to convert alkenes to arenes in the dual cycle mechanism, hence replacing the traditional methanol dehydrogenation route via formaldehyde. Together, this novel insight emphasizes the complexity and fine balance required between various parameters of the tandem process to ensure stable formation of the desired product mixture over time. It also opens an exciting new playground for fundamental studies of site–structure–conditions–function correlations in this new process.

A special case of the methanol-mediated tandem reaction occurs when C_2_ products are formed on the CO_x_ hydrogenation catalyst alongside methanol. Ketene, acetyl, acetate, acetic acid, and ethene (or its hydrated analogue ethanol) are all constituents of the initiation mechanism of the hydrocarbon pool in traditional MTH chemistry. When those C_2_ compounds are formed externally to the zeolite/zeotype, the slow step of the autocatalytic MTH reaction, initial C–C bond formation, may become redundant, potentially yielding higher product formation rates. When such acceleration is not observed in tandem studies, it may be due to the high H_2_O/methanol ratio induced by the thermodynamic limitation of methanol formation under tandem conditions and further enhanced by the competitive adsorption of these two products on the BAS of the zeolite/zeotype. Another reason may be the slow diffusion or low reactivity of C_2_ products formed externally, especially in small-pore (8-ring) zeolites/zeotypes: the high ethene selectivity observed in the studies referred to above, where ethanol or ketene was produced externally, suggests that they were poorly integrated in the hydrocarbon pool. Nevertheless, the growing number of materials capable of catalyzing C_2_ compound formation from CO_2_/H_2_, some of them referred to in this Review, pave the way for new directions within materials development for the oxygenate-mediated conversion of CO_2_/H_2_ to hydrocarbons: Brønsted and Lewis acid sites and their surrounding matrix may be tuned for optimal performance, inside and outside the microporous window/pore/cavity structure of the zeolite/zeotype.

In the Introduction, we promised a comparison between methanol versus DME and ketene as mediators in the oxygenate-mediated conversion of CO_2_/H_2_ to hydrocarbons. As an initial statement, recent studies clearly established that both DME and ketene are formed from the methanol feed in the zeolite/zeotype-catalyzed MTH process. Hence, the core of this comparison must be whether it is advantageous to add to the reactor another catalyst component that promotes formation of DME or ketene, beyond the lattice components of the zeolite/zeotype crystals.

Starting with a comparison between methanol and DME, it is clear that in a dual reactor concept, higher yields of DME than methanol are attainable in the first reactor (CO_2_/H_2_ to oxygenate) due to thermodynamic limitations. The introduction of a methanol dehydration catalyst in this first reactor will reduce the heat release in the second reactor and enable separation of more H_2_O from the effluent of the first reactor compared to having methanol as the oxygenate mediator. Potential water separation between the reactors will reduce competitive water sorption on the Brønsted acid sites in the second reactor, thereby enhancing conversion rates and mitigating catalyst degradation. When adding the established role of methanol as a hydrogen transfer agent in aromatics and coke formation during MTH operation (to be reconsidered under CO-rich conditions, vide ultra), the advantages of DME-mediated dual-reactor operation seem overwhelming. The fact that DME production from synthesis gas is already an integral part of several industrial processes for hydrocarbons production (MTP, TIGAS, and DMTO) adds to this picture. However, the influence of H_2_, CO_2_, and CO cofeed with DME in the second reactor is yet to be studied in detail and could need further attention.

Proceeding to the single-reactor approach, where CO_2_ hydrogenation and oxygenate conversion catalysts are mixed, the effect of introducing a methanol dehydration catalyst to establish methanol–DME equilibrium is less straightforward. The slower diffusion of DME versus methanol into 8-ring window-cavity structures might require the dehydration function to be installed inside the cavities, which is where product formation takes place. In medium- to large-pore zeolites and zeotypes, such as ZSM-5, promotion of methanol dehydration has been shown to yield positive effects on catalyst selectivity toward effluent products instead of coke under MTH conditions. Still, detailed studies of H_2_, CO_2_, and CO cofeeds with DME (suggested above), as well as the effect of DME/water versus methanol on the dual catalyst functions, are yet to be pursued.

Proceeding next to ketene-mediated routes to hydrocarbons, individual studies of zeolites/zeotypes and metal oxides, respectively, showed that a far higher CO/methanol ratio is needed to carbonylate methanol to ketene in the Brønsted acid-based zeolites/zeotypes compared to dense metal oxides, which have mainly Lewis acid character. Hence, there are clear advantages to external ketene production. As pointed out above, ketene may “kick-start” the MTH reaction in the zeolite/zeotype, provided that the zeolite/zeotype pores are big enough to enable ketene to enter and react. Otherwise, ketene may be a source of a high ethene selectivity. On the opposite side, too high ketene concentrations could be a source of coke formation, so a right balance of conditions leading to a suitable steady-state concentration is important.

Overall, the wealth of literature studies referred to in the individual chapters and subchapters of this Review provide design rules for dense as well as microporous catalysts leading to the formation of either methanol, DME, or ketene in the presence of the molecular precursors to each product. The subchapters devoted to the use of those products as mediators in the tandem reaction reveal further design options for catalyst, process, and reaction conditions. Although some critical issues remain to be solved, we are confident that the current state-of-the-art knowledge represents a stepping-stone for further important discoveries and developments in the years to come, paving the way for a successful transition to the postfossil society.

Among future research opportunities, we highlight:

(1) Catalyst optimization:

- A wealth of zeolite/zeotype structures, compositions, and BAS/LAS siting are yet to be explored for the tandem reaction. Inspiration for catalyst design may be found from prior studies cited herein.
- Catalyst morphology may be altered to facilitate product diffusion.
- Rapid/selective DME or C_2_ formation before the zeolite/zeotype function may accelerate hydrocarbons formation in the zeolite/zeotype compared to methanol-mediated routes and mitigate catalyst deactivation, and should be further explored.
- Long-term catalyst performance, in particular the effect of repeated activation-reaction-regeneration cycles, is underexplored and needs further attention.
- Potential element migration within and between catalyst functions during synthesis, activation, testing, and regeneration calls for advanced catalyst characterization studies before, during, and after test cycles.
- Organo-catalysis by guest species in zeolite/zeotype pores and cavities (beyond conventional reaction intermediates and products) may promote desired reactions.^315^

(2) Process optimization:

- Continuous water removal by membrane- or sorbent-based reactor design may enhance hydrocarbon formation rates, potentially at the expense of TON. Studies of the effect of CO_2_, CO, H_2_, and H_2_O concentration on process thermodynamics and kinetics is recommended, in particular for the less-explored oxygenate intermediates (DME and C_2_).
- Two temperature zones in a single reactor with a first temperature zone for DME synthesis and a second temperature zone for DME conversion to hydrocarbons is an interesting opportunity. The dual- versus single-reactor tandem options should be compared.
- Alternative heating methods may be worthy of consideration.^316−318^

As a final remark, the purpose of using CO_2_ as carbon feedstock to the chemical industry is to mitigate the negative impact of human activity on our living environment. In this context, the final selection of catalyst, process, and feedstock for industrial implementation of the oxygenate-mediated conversion of CO_2_ and H_2_ to hydrocarbons should not rely only on economic and energy considerations of the individual process but include cradle-to-grave Life Cycle Analysis of all involved elements.
